# Annealed leap-point sampler for multimodal target distributions

**DOI:** 10.1007/s11222-026-10988-5

**Published:** 2026-10-17

**Authors:** Nicholas G. Tawn, Matthew T. Moores, Hugo Queniat, Gareth O. Roberts

**Affiliations:** 1 Department of Statistics, University of Warwick, Coventry, CV4 7AL UK; 2 School of Engineering Science, Lappeenranta-Lahti University of Technology, Yliopistonkatu 34, Lappeenranta, 53850 Finland

**Keywords:** Parallel tempering, Mode-jumping proposals, Curved exponential family, Seemingly-unrelated regression, Panel data, Raman spectroscopy

## Abstract

In Bayesian statistics, exploring high-dimensional multimodal posterior distributions poses major challenges for existing MCMC approaches. This paper introduces the annealed leap-point sampler (ALPS), which augments the target distribution state space with modified annealed (cooled) distributions, in contrast to traditional tempering approaches. The coldest state is chosen such that its annealed density is well-approximated locally by a Laplace approximation. This allows for automated setup of a scalable mode-leaping independence sampler. ALPS requires an exploration component to search for the mode locations, which can either be run adaptively in parallel to improve these mode-jumping proposals, or else as a pre-computation step. A theoretical analysis shows that for a d-dimensional problem the coolest temperature level required only needs to be linear in dimension, $\mathcal {O}\left( d\right)$, implying that the number of iterations needed for ALPS to converge is $\mathcal {O}\left( d\right)$ (typically leading to overall complexity $\mathcal {O}\left( d^3\right)$ when computational cost per iteration is taken into account). ALPS is illustrated on several complex, multimodal distributions that arise from real-world applications. This includes a seemingly-unrelated regression (SUR) model of longitudinal data from U.S. manufacturing firms, as well as a spectral density model that is used in analytical chemistry for identification of molecular biomarkers.

**Supplementary Information:**

The online version contains supplementary material available at https://doi.org/10.1007/s11222-026-10988-5.

## Introduction

The standard approach to sampling from a multivariate Bayesian posterior density is Markov Chain Monte Carlo (MCMC). There is a wealth of established literature and methodology enabling the construction of a Markov chain with prescribed invariant distribution (e.g., Robert and Casella (2004)). Arguably the easiest way to construct such a chain is by using a localised proposal mechanism e.g., random walk Metropolis (Metropolis et al. 1953), Metropolis-adjusted Langevin (Rossky et al. 1978; Roberts and Tweedie 1996), or hybrid methods formed from concatenating local moves such as Hamiltonian Monte Carlo (Duane et al. 1987). Such proposals can then be accepted or rejected according to the Metropolis-Hastings acceptance probability (Hastings 1970), ensuring reversibility of the chain.

When the target exhibits multimodality, the majority of MCMC algorithms that use localised proposals will fail to explore the state space, leading to biased estimation of posterior expectations from the sample output (Roberts and Rosenthal 2001; Mangoubi et al. 2018). Indeed, when the target distribution is high-dimensional or the posterior arises from a complex Bayesian modeling context then it becomes very hard to know whether the posterior is multimodal. Therefore it is important to develop methods that are robust but also scale well to high-dimensional settings.

The most popular MCMC methods designed to explore multimodal distributions have traditionally been based on *tempering* approaches and building on the seminal contributions of Geyer (1991); Marinari and Parisi (1992); Geyer and Thompson (1995). However, despite the success of these methods, tempering approaches often fail in high-dimensional settings, and where individual modes have different variances and/or display asymmetries (Woodard et al. 2009a).

This paper introduces a new algorithm, the Annealed Leap-Point Sampler (ALPS), that overcomes many of these disadvantages. It is designed to sample from a class of smooth densities on $\mathbb {R}^d$ that may exhibit multimodality. The ALPS construction uses an annealing approach, where contrary to traditional approaches it relies on raising the density to powers greater than one. It also adapts ideas from established methods in the literature designed to mitigate the worst effects of multimodality in multi-dimensional settings, such as mode-jumping proposals (Tjelmeland and Hegstad 2001) and weight-stabilised tempering (Tawn and Roberts 2018; Tawn et al. 2019) that incorporate non-local information about the target.

As well as presenting novel methodology, this paper introduces accompanying theoretical results that describe its computational complexity (under suitable regularity) and which underpin practical guidance on temperature selection for the algorithm. The conclusion is that the largest power $\beta _{\text {max}}$ in the sequence of annealing distributions should be selected so that it scales as $\mathcal {O}(d)$. Therefore, building on the theoretical results in Roberts et al. (2022) we are able to deduce that the number of iterations required should scale as $\mathcal {O}(d)$, typically leading to an overall computational cost complexity of $\mathcal {O}\left( d^{3}\right)$ for ALPS when taking into account computational expense per iteration. See further discussion in Sect. 6. It should be noted that these complexity calculations do not include the cost of searching for the mode locations, an essential problem for all multi-modal MCMC approaches, and which is a challenging problem in its own right. We incorporate an exploration component in ALPS that searches for new modes, as well as maintaining a list of modes that it has already found. This can either be run as one or more parallel chains to adaptively improve the mode-jumping proposal distribution, or else as a pre-computation step separate from ALPS.

The merits of the ALPS algorithm are demonstrated empirically, firstly on a synthetic example where the modes are known, then on a Seemingly-Unrelated Regression (SUR) model for manufacturing investment data (Grunfeld 1958), and lastly on a peak decomposition model for Raman spectroscopy (Moores et al. 2026). Bayesian analysis of spectroscopy was pioneered by Ritter (1994) and van Dyk et al. (2001). It is a proven approach in analytical chemistry for many different types of spectroscopic data (e.g., Wang et al. (2008); Zhong et al. (2011); Härkönen et al. (2023)). As with SUR, this is a popular model for applications but its problems with multimodality have seldom been acknowledged or addressed.

SUR models were introduced by Zellner (1962) and are widely used for panel data in econometrics. Many examples are provided by Srivastava and Giles (1987) and Fiebig (2003). It has only relatively recently been established that the SUR model likelihood exhibits multimodality and that the current methodology for inference is not robust. Our paper is the first time that multimodality has been demonstrated for the Grunfeld (1958) data. We show that ALPS can successfully overcome the difficulties in both the synthetic problem as well as the complex real-data applications of SUR and spectroscopy.

The paper is structured as follows: after giving background and motivation in Sect. 2, Sect. 3 provides a detailed description of each component of ALPS; Sect. 4 covers their assembly into the complete method; Sect. 5 presents the main theoretical contribution of the paper that is crucial to understanding the computational cost of the procedure; Sect. 6 discusses the computational cost and how it scales with the dimension of the problem; Sect. 7 contains empirical studies; and Sect. 8 presents our conclusions and considerations for further work. Detailed proofs, along with full R source code for the applications, are contained in the online supplementary material.

## Background and motivation

Consider the problem of sampling from a *d*-dimensional smooth density function $\pi (\cdot )$, where it is either known or suspected that the target distribution is multimodal. Such problems arise frequently across many fields e.g., astrophysics (Feroz et al. 2009); biology (Ballnus et al. 2017) or signal processing (Fong et al. 2019). Additionally, multimodality can arise due to non-identifiability in statistical models, as commonly seen in neural networks where symmetries in parametrization induce multiple equivalent modes (Papamarkou et al. 2022). These kinds of multimodal scenarios are well known to present significant challenges for even the most advanced MCMC algorithms, often resulting in poor mixing or incomplete exploration of the posterior distribution (e.g., Mangoubi et al. (2018)).

There is a significant volume of existing research focused on overcoming the multimodal sampling problem, which can be roughly designated into one of two approaches. The most direct approach uses “mode jumping” MCMC proposals, e.g. Tjelmeland and Hegstad (2001), Liu et al. (2000), Tjelmeland and Eidsvik (2004), Ibrahim (2009), Zhou (2011a, 2011b) and Pompe et al. (2020).

However the most successful and widely adopted approach to the problem are based on state-space augmentation techniques. These stem from two seminal, closely related but independently discovered algorithms: the parallel tempering (PT) algorithm (Geyer 1991) and its closely-related cousin, the simulated tempering algorithm (Marinari and Parisi 1992). The principle behind these methods is that a Markov chain can achieve global exploration when targeting a tempered version of the density ($\pi ^\beta$ for some 0<β<1). Therefore the strategy is to construct an MCMC algorithm on an augmented space, ${\mathbb {R}^d \times \Delta }$ where $\Delta =\{\beta _j, j=0, \ldots n\} \subset (0,1]$, with $\beta _0=1$ denoting the temperature of interest. Since π is assumed to be multi-modal, direct Markov chain transitions between its modes are likely to be very difficult. However, indirect paths between these modes can be achieved by moving to *hotter* temperatures (smaller β) where inter-mode transitions are feasible, and then moving temperature back to $\beta _0=1$.

The success of this simple strategy has inspired generalisations to a more general class of augmentation methods, see for example Wang and Swendsen (1990), Geyer (1991), Marinari and Parisi (1992), Neal (1996), Kou et al. (2006), Atchadé and Liu (2010), Nemeth et al. (2019), Tawn and Roberts (2019) and Syed et al. (2021).

Both classes of approaches have their strengths and weaknesses. Part of the motivation for the novel methodology presented in this paper is to design an algorithm that combines their strengths while mitigating as many of their weaknesses as possible.

**Fig. 1:**
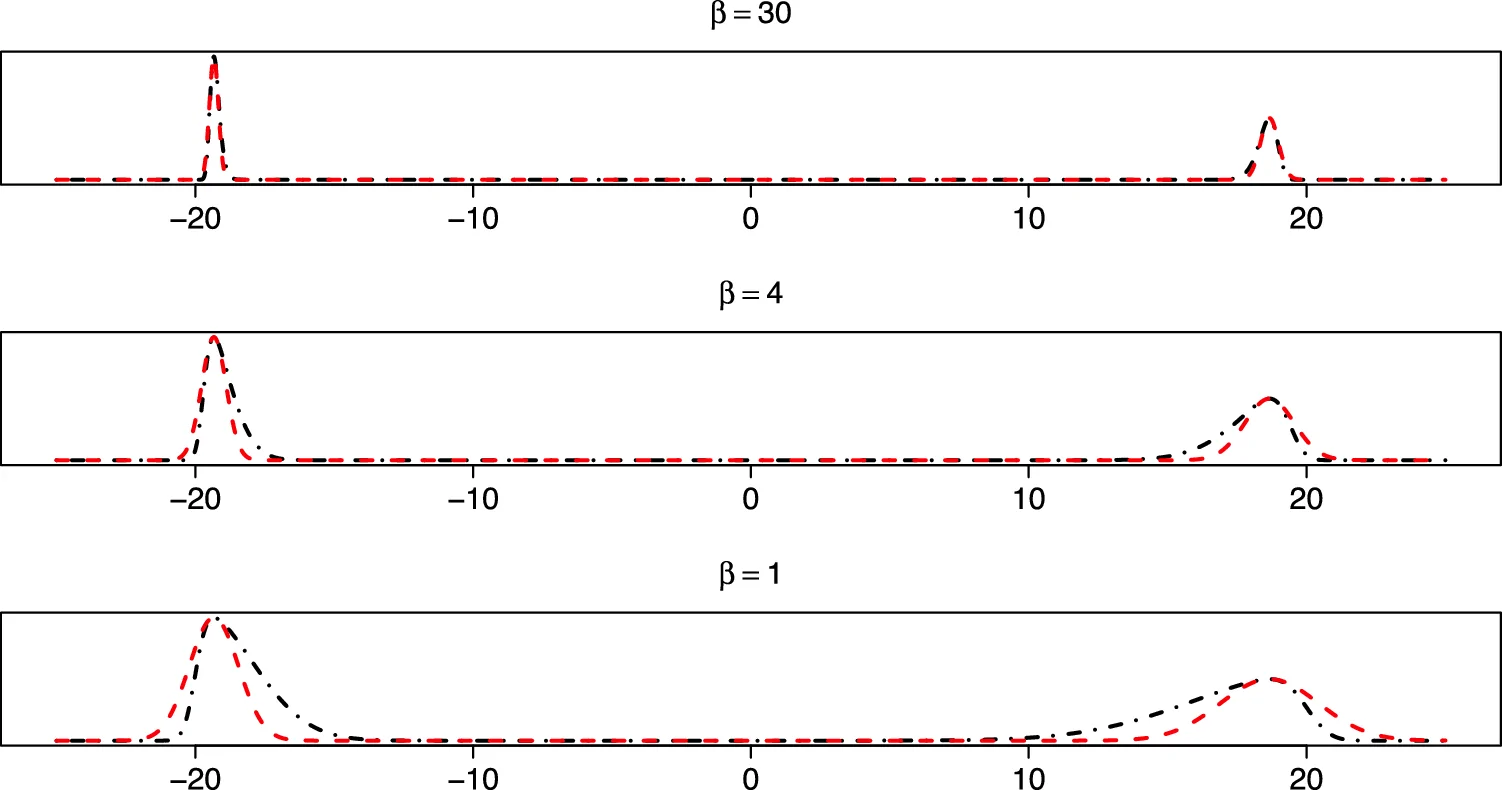
Annealing reduces the effect of skew. The dot-dashed line in the bottom panel shows a bimodal density with appreciable skewness; overlayed by the red dashed line representing the Gaussian mixture approximation derived through a local Laplace approximation. The two figures above this show annealed versions of the density and its respective approximation (at annealings β=4 and β=30 respectively); demonstrating that as the modes are annealed the deviation from local Gaussianity reduces

Despite their many successes, there are significant negative aspects regarding the performance of tempering-based MCMC approaches which afflict these algorithms particulary in moderate to high-dimensional settings, e.g. Woodard et al. (2009b), Woodard et al. (2009a) and Bhatnagar and Randall (2016). These problems are caused by weight degeneracy, whereby a mode which may have significant probability mass in the temperature of interest could become negligible at a different temperature. There have been recent developments in Tawn and Roberts (2018, 2019) that mitigate these problems and thus accelerate the performance of PT. However, these approaches lack robustness when the modal components of the target distribution exhibit significant asymmetry and heterogeneity of scale as illustrated in Fig. 1. Tawn et al. (2019) attempts to address the issues of heterogeneous scale, by applying tempering in a localised way. However, there remain questions over the high-dimensional practicality of the approach, especially regarding the Markov chain’s convergence properties for the tempered target distributions that are essential to ensuring successful global exploration.

Alternative approaches, e.g. Tjelmeland and Hegstad (2001) and Ibrahim (2009), attempt to approximate the target distribution via local Laplace approximations in order to design appropriate inter-modal MCMC moves. However, their mixing times increase exponentially in dimension (Liu 1996). Performance is particularly problematic where the modal components exhibit skewness. Even in one dimension, the best Gaussian mixture approximation to the target distribution might not be a good fit, see for example Fig. 1 at β=1.

Overcoming these problems and developing a practically applicable algorithm is the motivation behind this work. The most significant novelty in this work is that an annealing approach (i.e. powers β>1) is adopted to reduce the skew of any particular local component, making it appear approximately Gaussian. From the upper two plots in Fig. 1 we can clearly see the effect of reducing skew by annealing. This greatly facilitates the design of a Markov chain that has efficient inter-modal mixing properties (in practice we use a mode-hopping independence Metropolis-Hastings sampler).

We shall show that this ambitious plan can be accomplished by giving detailed methodology and supporting theory that demonstrates its robustness in high dimensional contexts. In particular, we demonstrate that the proposed annealing strategy achieves linear mixing-time scaling in dimension, addressing regimes in which standard tempering methods may exhibit exponentially slow convergence, e.g. Woodard et al. (2009a).

This linear behaviour persists even for well-separated, skewed modes and heterogeneous covariance structures, ensuring efficient mixing as dimension increases. The resulting gain in Markov chain convergence shifts the complexity from exponential to polynomial, at the expense of a modest increase in per-iteration computational cost, preserving polynomial-time scalability.

## Components of ALPS

ALPS consists of two components, *Parallel Annealing* (ALPS-PA) which is an annealing “parallel tempering” scheme and the *Exploration Component* (ALPS-EC) which is a procedure aimed at exploring the whole state space to identify the locations of modes. These two processes can be run in parallel essentially creating an adaptive MCMC algorithm (Roberts and Rosenthal 2009).

As with any parallel tempering method, ALPS-PA constructs a Markov chain on the space $\mathbb {R}^{d(n+1)}=\prod _{j=0}^n\mathcal{X}_{\beta _{j}}$, where each $\mathcal {X}_{\beta _j}$ is an independent copy of $\mathbb {R}^{d}$ We denote the value of the chain at iteration *t* by $X^t:= ( x_0^t,\ldots ,x_n^t)$, which is targeting an invariant distribution

1 $$\begin{aligned} \pi _{ALPS}(X) \propto \prod _{j=0}^n \pi _{\beta _j}(x_j), \end{aligned}$$

where $\pi _{\beta _j}$ are an appropriate sequence of densities. Crucial to the success of ALPS-PA though will be

(3.1) the choice of the auxiliary densities $\pi _{\beta _j}$;
(3.2) the tempering schedule $\Delta = \{\beta _0, \beta _1, \ldots , \beta _{n} \}$, which determines a sequence of auxiliary probability densities;
(3.3) the choice of temperature swap moves that have accelerated mixing properties through the aid of a deterministic transformation between $\mathcal{X}_{\beta _j}$ and $\mathcal{X}_{\beta _{j\pm 1}}$;
(3.4) the designing of $P_{\beta _j}(\cdot ,\cdot )$, the Markov chain transition mechanism for moves conditional on $\beta _j$, facilitating *within-mode* mixing;
(3.5) the collection of transition kernels $\{ Q_{\beta _0}(\cdot ,\cdot ),Q_{\beta _1}(\cdot ,\cdot ),\ldots ,Q_{\beta _n} (\cdot ,\cdot ) \}$ designed to allow *inter-modal* jumps;
(3.6) the mode finding exploration component of ALPS to discover the local maxima of the target distribution.

The coming subsections will be devoted to describing and justifying the choices made in (3.1)–(3.6).

Tjelmeland and Hegstad (2001) and Pompe et al. (2020) showed that if the modes can be well-approximated by Gaussian distributions (typically derived from a Laplace approximation) then an independence sampler-type proposal based on a Gaussian or Gaussian mixture is very effective for mode-jumping.

However, in high dimensional settings the challenge is that even small deviations (marginally) from Gaussianity lead to degenerate acceptance rates of such moves. Pompe et al. (2020) overcome this by fitting a more involved Gaussian mixture to the often complex modal structures. ALPS takes a different approach, it seeks to naturally and automatically adapt the target distribution into something that is increasingly well-approximated by a Gaussian mixture distribution with well-separated components i.e., a target where all modes appear locally Gaussian.

A central component in the design of the algorithm is the following Gaussian mixture distribution. Given a list of vectors $M=\{\mu _j,\ 1\le j\le m\}$, a list of symmetric positive-definite matrices $S=\{\Sigma _j,\ 1\le j\le m\}$, and a list of positive weights summing to unity $W=\{w_1, \ldots , w_m\}$, we set

2 $$\begin{aligned} \pi _{M,S,W}(x) = \sum _{j=1}^m w_j \phi \left( x|\mu _j,{\Sigma _j}\right) \end{aligned}$$

where $\phi (\cdot |\mu _j,\Sigma _j)$ denotes the pdf of a multivariate Gaussian with mean $\mu _j$ and covariance matrix $\Sigma _j$.

If the target distribution has modes that locally resemble Gaussian distributions, it can naturally be approximated effectively by a Gaussian mixture like the one in (2), where *m* would correspond to the number of local maxima. In such cases, provided the Gaussian mixture parameters can be derived, the problem simplifies considerably. Indeed, by combining localized MCMC moves with independence sampler moves drawn from the proposal distribution given in (2), one can design a Markov chain that would yield very rapid convergence.

ALPS aims to exploit this construction with its very rapid convergence properties even in cases where the target has modes that deviate substantially from appearing Gaussian.

### Hessian-adjusted tempered (HAT) distributions

Traditionally, tempering algorithms rely on augmentation strategies using powered densities where the entire density is raised to some power β<1. This has the effect of merging modes and thus facilitating transitions between different modal domains of attraction.

Woodard et al. (2009a) shows that when modes of a multimodal target exhibit heterogeneity, particularly with regards to their scales, then these power-based target distributions are a poor choice as they lead to torpid mixing of PT when the dimensionality of the problem grows. Unless the distributions are altered, annealing is subject to similar challenges of torpid mixing. The chains targeting the most annealed distributions become increasingly restricted to the dominant modes corresponding to global maxima, as in simulated annealing (Aarts and Korst 1989).

The target distributions used in the PA component build upon on recent work in Tawn et al. (2019) which aims to (approximately) preserve mass that one may associate with each mode across different temperature levels.

First, we introduce an approximate partition of the state space into domains (or basins) of attraction, in the sense of Tjelmeland and Hegstad (2001); Zhou (2011a). In this context, the domain of attraction $\mathcal {D}_j \subseteq \mathcal {X}$ associated with mode $\mu _j$ is defined as the set of points from which the gradient flow dynamics (i.e., the solutions to the gradient-flow ODE) converge to $\mu _j$, as introduced in Zhou (2011a). Given the Gaussian mixture distribution $\pi _{M,S,W}$, we derive an approximation of these domains, based on a modified Mahalanobis distance criterion (dependent on *M*, *S*, *W*, although we omit this explicit dependence for notational convenience).

Formally, the partitioning is provided by the following *mode assignment function* at inverse temperature β,

3 $$\begin{aligned} A_{x, \beta }:= \mathop {\mathrm {arg\,max}}\limits _{j \in \{1,\dots ,m\}} \left\{ w_j\phi \left( x\mid \mu _j,\frac{\Sigma _j}{\beta }\right) \right\} \end{aligned}$$

where $\phi (\cdot |\mu _j,\Sigma _j)$ denotes the pdf of a multivariate Gaussian with mean $\mu _j$ and covariance matrix $\Sigma _j$.

This assignment produces a partition consistent with the gradient-flow definition of domains of attraction when the target distribution is a Gaussian mixture. Through annealing, the modes approach local Gaussianity, and the approximation of these domains becomes increasingly accurate. Using this partition, we follow Tawn et al. (2019, Def. 2) and restate the Hessian Adjusted Tempered (HAT) distributions, which we adopt as our auxiliary densities $\pi _{\beta _j}$.

#### Definition 3.1

*(Hessian Adjusted Tempered (HAT) distributions)* Given an inverse-temperature level β and its associated *mode assignment function* $A_{x, \beta }$, the HAT distribution at inverse temperature β is defined as

4 $$\begin{aligned} \pi _{\beta }(x) \propto \pi (x)^{\beta } \pi (\mu _{A_{x, \beta }})^{1-\beta }. \end{aligned}$$

Theoretical justification for these distributions can be found in the supplementary material in Appendix B.1. In short, this form of the target distribution can mitigate some of the most undesirable weight stabilisation issues exhibited by traditional tempering approaches.

### Choice of inverse temperatures

ALPS-PA will carry out an MCMC algorithm on the density

$$ \Pi (x_0, \ldots ,x_n) \propto \prod _{i=0}^n \pi _{\beta _i} (x_i) $$

where $\Delta = \{\beta _0, \beta _1, \ldots , \beta _n\}$ is a user-defined *temperature schedule*. By convention $\beta _0$ is taken to be 1, the temperature of interest. Unlike other tempering approaches, the other temperatures, $\Delta -\{\beta _0\}$, are taken to be a collection of colder temperatures, so that $\beta _0<\beta _1< \ldots < \beta _n$.

There are two important aspects to the choice of Δ, the choice of $\beta _n$ and the spacing of the intermediate temperatures. For the former problem, we will derive theory in Sect. 5 which shows that we need to take $\beta _n=\mathcal {O}(d)$.

The spacings between consecutive temperatures in Δ must be chosen carefully. Appropriate choices maximize the round-trip rate, which measures how frequently complete cycles occur–beginning and ending at $\beta _0$, with an intermediate visit to $\beta _n$ (Syed et al. 2021). Optimizing this rate ensures rapid transfer of mixing information from the most annealed state to improve mixing at the target state. If spacings are too large, the acceptance rates for temperature swaps become excessively low. Conversely, excessively small spacings lead to slow mixing (and increased computational complexity) since there are too many intermediate temperature levels and it takes a long time to pass the information through the temperature schedule.

Appealing to the theoretical contributions of Tawn and Roberts (2018), Tawn and Roberts (2019) and Tawn et al. (2019), it is approximately optimal to select the spacings consecutively with $\beta _{j+1}=\beta _{j} +\epsilon _j$ where $\epsilon _j = \mathcal {O}\left( d^{-1/2}\right)$. Crucially, the optimal choice for the spacings corresponds to temperature swap acceptance rates between consecutive temperatures of 0.234. A practitioner can use this to tune the setup of the algorithm; even pursuing an adaptive strategy similar to Miasojedow et al. (2013).

Implementing this scheme robustly would usually start with a geometrically-spaced schedule (optimal for all power densities as shown in Atchadé et al. (2011)), and then fine-tuned to approach the optimal acceptance rate at all temperatures. Details can be found in Tawn and Roberts (2018, 2019).

### Design of temperature swap moves

Within ALPS-PA we shall make extensive use of the QuanTA transformation (as originally introduced in Tawn and Roberts (2019)).

#### Definition 3.2

*(QuanTA Transformation)* Given a tempering schedule Δ with $\beta _0<\ldots < \beta _n$ and lists *M*, *S*, *W*, then define $T^+_{M,S,W}:\mathbb {R}^d \times \{\beta _0, \ldots , \beta _{n-1}\} \rightarrow \mathbb {R} ^d\times \{\beta _1, \ldots , \beta _{n}\}$ and $T^-_{M,S,W}:\mathbb {R}^d \times \{\beta _1, \ldots , \beta _{n}\} \rightarrow \mathbb {R} ^d\times \{\beta _0, \ldots , \beta _{n-1}\}$ by

5a $$\begin{aligned} T^+_{M,S,W}(x,\beta _i)&= \left( \left( \frac{\beta _i}{\beta _{i+1}} \right) ^{1/2} (x-\mu _{A_{x,\beta _i}}) + \mu _{A_{x,\beta _i}}, \ \beta _{i+1} \right) , \end{aligned}$$

5b $$\begin{aligned} T^-_{M,S,W}(x,\beta _i)&= \left( \left( \frac{\beta _i}{\beta _{i-1}} \right) ^{1/2} (x-\mu _{A_{x,\beta _i}}) + \mu _{A_{x,\beta _i}}, \ \beta _{i-1} \right) . \end{aligned}$$

Note that, restricted to points for which the transformation remains in the same basin of attraction, the maps $T^+_{M,S,W}$ and $T^-_{M,S,W}$ are injective (though not surjective). Hence their inverses are well defined on their respective images within this reversibility-preserving region. If a proposed transformed point crosses into a different basin, this restriction fails and such proposals are immediately rejected in Step 3. below.

As in the standard framework of parallel tempering, pairs of adjacent temperature level components are chosen at random and for a swap move. Traditionally this simply involves proposing a swapping of the locations of the adjacent temperature components. Tawn and Roberts (2019) introduced a novel swap move that simultaneously proposes a deterministic transformation based on (5) with the motivation of accelerating the mixing through the temperature space.

At time *t*, the procedure for a temperature swap move in ALPS follows a very similar strategy to that in Tawn and Roberts (2019):

1. Select a pair of temperatures $\beta _i,\beta _{i+1}$ uniformly at random
2. Compute the transformations
  $$\begin{aligned} y_{i+1}=\left( T^+_{M,S,W}(x_i^t,\beta _i)\right) _1~~~\text{ and }~~~y_{i}=\left( T^-_{M,S,W}(x_{i+1}^t,\beta _{i+1})\right) _1 \end{aligned}$$
3. Check whether the transformations preserve basin membership which is required for reversibility. Reject the proposed swap if
  $$ A_{x^t_i,\beta _i }\ne A_{y_{i+1},\beta _{i+1} }~~~\text{ or }~~~A_{x^t_{i+1},\beta _{i+1} }\ne A_{y_{i},\beta _i } $$
  and set $x_{i+1}^{t+1}=x_{i+1}^t$ and $x_{i}^{t+1}=x_{i}^t$.
4. If 3. does not lead to rejection then compute
  $$ \alpha = \min \left\{ 1, \frac{\pi _{\beta _{i}}(y_i)\pi _{\beta _{i+1}}(y_{i+1})}{\pi _{\beta _{i}}(x_i^{t})\pi _{\beta _{i+1}}(x_{i+1}^{t})} \right\} . $$
  With probability α we accept a transformation-aided swap move and set
  $$ x_{i+1}^{t+1}=y_{i+1}~~~ ~~x_{i}^{t+1}=y_{i}$$
  else set
  $$ x_{i+1}^{t+1}=x_{i+1}^t~~~ ~~x_{i}^{t+1}=x_{i}^t.$$

Tawn and Roberts (2019) show that, under mild smoothness conditions, for large inverse temperatures β the traditional parallel tempering scheme is optimal with spacings that are $\mathcal {O}(\beta )$, while this transformation-enhanced swapping scheme can achieve spacings that are up to $\mathcal {O}(\beta ^{3/2})$ and $\mathcal {O}(\beta ^{5/4})$ when the modes are symmetric and asymmetric respectively in a neighbourhood of the mode point.

The transformation is more effective when the modes exhibit approximately Gaussian structure. For robustness in highly degenerate cases where QuanTA swaps may start to fail or, for instance, when modes are highly non-Gaussian, occasional traditional swap moves may optionally be included near the target temperature. In practice, we did not observe such behaviour, and empirical evidence suggests that this safeguard is not necessary.

### Choice of within-mode transitions

Algorithm moves that reposition particles within each temperature level are crucial for the algorithm’s success. The purpose of these moves is not to transition between modes–this is exclusively attempted at the highest inverse temperature $\beta _n$, see Roberts et al. (2022). At all other temperatures, we only require that the sampler mixes adequately within each mode. Although this paper does not specify a particular within-temperature Markov chain proposal for ALPS, careful tuning of these localized moves remains crucial and will certainly be a function of the temperature level such that tuning would be needed for all temperature levels, as in Parallel Tempering.

To increase robustness, we recommend employing sampling schemes that explicitly exploit local geometric properties which can differ significantly between modes, e.g. pre-conditioned Random Walk Metropolis. There are many established results for tuning the within-temperature marginal updates to the chain performed in PA component, e.g. Roberts and Rosenthal (2001).

For our purposes, we have found a suitable approach to be the pre-conditioned variant of the multi-dimensional Random Walk Metropolis algorithm, in which the proposal covariance is determined by the negative inverse Hessian of the log-target evaluated at each mode. Specifically, at position *x* and inverse temperature β, the Gaussian increment will have

$$\begin{aligned} {\Sigma } = -\left[ \nabla ^2 \log \pi ({\mu }_{A_{x,\beta }})\right] ^{-1}, \end{aligned}$$

as the proposal covariance. This strategy leverages local curvature information to enhance sampling efficiency, while avoiding the tuning challenges associated with more sophisticated algorithms such as Hamiltonian Monte Carlo (Neal 2011).

### Choice of mode-hopping mechanism

Using the collections *M*, *S*, and *W*, we can construct a Gaussian mixture target distribution indexed by a temperature β given by:

6 $$\begin{aligned} q_{\beta }(x| M,S,W):= \sum _{j=1}^m {w}_j \phi \left( x ~\Big |~\mu _{j},\frac{\Sigma _j}{\beta }\right) . \end{aligned}$$

The transition kernel $Q_\beta$ will thus be constructed for inverse temperature β as an independence sampler with proposal density $q_{\beta }(\cdot | M,S,W)$ and target distribution $\pi _{\beta }$. Given current state *x* and proposed new state *y* drawn from this proposal density, the move to *y* is accepted with probability

7 $$\begin{aligned} A_{IS}(x,y| M,S,W):= \text{ min }\left( 1,\frac{\pi _\beta (y) q_{\beta }(x | M,S,W)}{\pi _\beta (x) q_{\beta }(y | M,S,W)}. \right) \nonumber \\ \end{aligned}$$

This move will predominantly only be attempted at the highest inverse temperature $\beta _n$, as depicted in Roberts et al. (2022).

The collections *M*, *S* and *W* will be estimated via Laplace approximations as detailed in Sect. 3.6. If adequate annealing has taken place so that the Laplace approximations for the local modes are reasonably accurate then the weight approximations will closely match the regional masses in the $\beta _{\text {max}}$-level HAT target. In that case, the independence sampler constructed above should be able to achieve high acceptance rates.

Of course, any finite amount of annealing leaves some modal asymmetry and thus prevents acceptance rates being equal to 1. In general, one must anneal more as the dimensionality of the problem increases before the Laplace approximation becomes accurate. The question of how large $\beta _{\text {max}}$ should be with respect to the dimensionality of the problem to achieve sufficiently good performance is addressed in Sect. 5. In practice, to choose such a value of $\beta _{\text {max}}$, any target acceptance rate a∈(0,1) at the cold level achieves the corresponding complexity guarantees described in Sect. 6. A natural choice is therefore a=0.5, which can be increased if the resulting hopping rate is not sufficiently high for the problem at hand. Our empirical results do not indicate a clear preference for a particular value of a; indeed, Sect. 7 presents experiments covering acceptance rates ranging from 0.25 to 0.9. Nevertheless, for robustness, a practitioner may choose a higher target acceptance rate, such as 0.9, to ensure frequent hopping. This does not incur computational burden under the chosen temperature schedule in Sect. 3.3.

### Design of mode-finding procedure

Each of the previously described mechanisms relies on the collections *M*, *S*, and *W*, which represent respectively the locations, covariance structures, and weights of the modes. To determine this information for each mode, one could employ a range of approaches. In ALPS, this task is achieved by the *Exploration Component* (ALPS-EC) with an approach that appears to work adequately in the empirical studies.

ALPS-EC constructs a Markov chain that has an invariant distribution given by $[\pi (\cdot )]^{\beta _{\text {hot}}}$. If the inverse-temperature level, $\beta _{\text {hot}}$, is sufficiently low then, as in the standard PT algorithm, the Markov chain can explore the entire state space rapidly. After a pre-specified number of steps of the Markov chain, one performs a local optimisation (this work uses Quasi-Newton optimisation) initialised from the current location of the chain. If the optimum attained is a new mode point not previously encountered, one adds it to the collection of discovered modes.

In high-dimensional settings, or when modes are exceptionally close together, numerical instabilities can result in either false mode identification or redundant detection of modes. Both phenomena can degrade the algorithm’s performance. To mitigate this, we require that any newly identified mode be sufficiently separated from all previously detected modes.

To assess this separation, we compare a proposed new mode $\mu ^\star$ with associated local covariance estimate $\Sigma ^\star$, to each previously discovered mode $\mu _k$ and its associated covariance estimate $\Sigma _k$ through the following pseudo-distance:

8 $$\begin{aligned} D(\mu _k,\mu ^{*}):= d^{-1}\max \left\{ (\mu _k-\mu ^{*})^T \Sigma _k^{-1}(\mu _k-\mu ^{*}), \right. \nonumber \\ \quad \left. \mu _k-\mu ^{*})^T (\Sigma ^{*})^{-1}(\mu _k-\mu ^{*}) \right\} . \end{aligned}$$

This pseudo-distance is the maximum of two squared Mahalanobis distances, normalized by the dimension *d*, and therefore accounts for the local covariance structure around both the existing mode and the candidate mode, measuring the extent to which the two modes remain distinct once this local covariance structure is taken into account. We then enforce that this pseudo-distance between the candidate mode and all existing modes exceeds a prescribed threshold, chosen according to a pre-specified quantile of the chi-squared distribution with *d* degrees of freedom, with the normalization by *d* applied correspondingly. This criterion helps prevent the algorithm from registering numerically indistinguishable or excessively similar modes as distinct.

Using the pre-defined tolerance, we check if the pseudo-distances from the proposed new mode $\mu ^*$ to existing modes surpass this threshold. If so, we augment the estimated sets $\hat{M}$, $\hat{S}$, and $\hat{W}$ with the new mode $\mu ^*$; these sets correspond to the current estimates for *M*, *S*, and *W*, as outlined below:

- $\hat{M}:=\{ \hat{\mu }_1, \ldots , \hat{\mu }_{\hat{m}} \}$, the collection of mode points of the target distribution π, with $\hat{m}$ the number of unique mode points discovered so far;
- $$\hat{S}:=\{ \hat{\Sigma }_1, \ldots , \hat{\Sigma }_{\hat{m}} \}$$, where $$\hat{\Sigma }_j= -\left[ \nabla ^2 \log (\pi (\hat{\mu }_j))\right] ^{-1},~~j=1,\ldots , \hat{m}$$;
- $\hat{W}=\{\hat{w}_1,\ldots , \hat{w}_{\hat{m}} \}$, the collection of approximated weights
  $$\begin{aligned} \hat{w_j}= \frac{\pi (\hat{\mu }_j) |\hat{\Sigma }_j|^{1/2}}{\sum _{k=1}^{\hat{m}} \pi (\hat{\mu }_k) |\hat{\Sigma }_k|^{1/2}}, ~~j=1,\ldots ,\hat{m} \end{aligned}$$

The hot-state mode finder outlined above provides a straightforward implementation of mode detection, and determining optimal EC temperature levels remains a nuanced challenge. Specifically, there is a trade off between using temperature levels that are too hot therefore the chain drifts away from the modes, and too cold, leading to a chain that remains stuck in the local mode.

To go further and increase robustness, there are many ad-hoc modifications that can be considered: running a population of chains at different temperature levels; repeatedly refreshing the chain from already discovered modes; use different optimisation strategies; perform optimisation based on the curvature of the target; to only name a few.

We do not claim that our approach to mode finding is optimal, nor do we provide theoretical guarantees. The process of identifying modes is computationally intensive and highly problem-dependent; no single approach can be expected to work best in all scenarios. Improving the performance of the mode-finding procedure is a focus of further work beyond the scope of this paper. In practice, it may be beneficial to adapt ALPS–EC to the characteristics of the problem at hand. For instance, in machine-learning settings, where objective functions are often evaluated using minibatches and are therefore noisy, stochastic gradient-based methods such as SGD or Adam (Kingma and Ba 2015) are natural candidates for identifying regions of high density.

## ALPS: the procedure

Bringing together the ingredients of Sect. 3, we shall summarize the methodology underlying a complete iteration of the ALPS algorithm.

Starting from the current sample $X^t = (x_0^t, \dots , x_n^t)$ at iteration t, we illustrate how the tools previously introduced in Sects. (3.1)–(3.6) can be combined systematically to produce the subsequent sample $X^{t+1}$.

Given a user-defined temperature schedule Δ, see (3.2), and current collections $\{\hat{W},\hat{M},\hat{C}\}$, obtained through an online or prior running of ALPS-EC, see (3.6), ALPS-PA engages in the following:

1. Conduct the within-temperature updates for all inverse temperatures $\beta _j$;
  - For $0 \le j \le n-1$, draw $x_j^{t+1} \sim P_{\beta _j}(x_j^t,.)$ for local exploration, see (3.1) and (3.4);
  - For j=n, run the mode-hopping independence sampler, $x_n^{t+1} \sim Q(x_n^t,.)$, for global exploration, see (3.5).
2. Perform a predetermined number of s temperature swaps, through QuanTA-aided moves, as explained in (3.3);
3. Store the subsequent sample $X^{t+1} = (x_0^{t+1}, \ldots , x_n^{t+1})$, go to step 1.

### Remark 1

A comprehensive pseudocode version of ALPS, suitable for direct implementation, is presented in Appendix C of the online supplementary material.

## Theoretical underpinnings of ALPS

This section introduces theoretical results addressing how the highest inverse temperature, $\beta _{\text {max}}$, must scale with dimension. The choice of $\beta _{\text {max}}$ is crucial, as the effectiveness of the ALPS algorithm fundamentally relies on ensuring the target distribution at the PA component’s highest annealed level is well approximated by a Gaussian mixture distribution.

In general, as the dimensionality of the problem increases, $\beta _{\text {max}}$ needs to get larger to ensure that the local mode Laplace approximation is accurate. Hence choosing $\beta _{\text {max}}$ too small will mean that the PA component mixes poorly in the coldest temperature level.

Choosing $\beta _{\text {max}}$ extremely large to overcome this has a few issues. On a practical level, it may result in computational instability as modes collapse towards point masses. Furthermore, the HAT targets provide only approximate mass preservation, and care is therefore required when considering extremely large values of the inverse temperature; see Tawn et al. (2019). Finally, there is extra computational burden as one would need more (unnecessary) intermediate temperature levels in the PA component. Hence, one would like to choose $\beta _{\text {max}}$ sufficiently large to achieve non-degenerate acceptance rates but not so large that it makes round trips infrequent.

### Optimal scaling of the coldest temperature level

Roberts et al. (2022) demonstrates that, at least under stylised conditions, if we can ensure that jumping between modes is possible at the coldest temperature, then the entire algorithm (suitably time-scaled) converges to a process (Walsh Brownian Motion) which itself has a mixing time which is independent of *d*. (While the exact result (Theorem 4) in that paper assumes that jumping between modes at the cold temperature always occurs, it is easy to see from the proof that it extends routinely to just requiring that jumps happen with probability which are bounded away from 0.) Therefore this section will be dedicated to demonstrating that at a sufficiently cold inverse temperature (we need $\beta = \mathcal {O}(d)$) then the probability of accepting a move to a different mode in the cold temperature is bounded away from 0.

For the sake of tractability the theoretical analysis will be focused on an approximation of the ALPS procedure. Suppose that the target is a *d*-dimensional mixture distribution where the components are comprised of location and scale transformations of a common distribution.

Then, the approximation of the ALPS procedure that we analyse is a distribution on $\left\{ 1,2,\ldots ,m \right\} \times \mathbb {R}^{d}$ where the extra dimension is acting as an augmentation that identifies which mixture component the location is originating from. This is an extremely good approximation when the components are well separated which is the case at the coldest temperature levels that are being analysed here.

#### Assumptions on the target density

Assuming a d+1-dimensional state space $\{ 1,2,\ldots , m \} \times \mathbb {R}^d$, the tempered target distributions at inverse temperature β will be given by the iid product form

9 $$\begin{aligned} \pi _\beta (k,x) \propto \sum _{j=1}^m w_j &  \left( \prod _{i=1}^d \left[ \frac{1}{\sigma _{ij}} f\left( \frac{x_i -\mu _{ij}}{\sigma _{ij}}\right) \right] ^\beta \right. \nonumber \\ &  \qquad \left. \left[ \frac{1}{\sigma _{ij}} f\left( 0\right) \right] ^{1-\beta } \right) \mathbb {1}_{\{k=j\}}. \end{aligned}$$

where $\sigma _{ij} \in \mathbb {R}_+$, $\mu _{ij}\in \mathbb {R}$ and *f* is an un-normalised univariate density function such that:

1. Assume that
  10 $$\begin{aligned} f\in \mathcal {C}^5, ~f(0)=1, ~f'(0)=0 ~\text{ and }~\mathop {\mathrm {arg\,max}}\limits _x f(x) =0\nonumber \\ \end{aligned}$$
  with 0 being the unique global maximum.
2. Defining h(x):=logf(x), assume
  11 $$\begin{aligned} H:= h''(0)<0 \end{aligned}$$
  and that there exists $L\in \mathbb {R}_{+}$ such that
  12 $$\begin{aligned} |h'''''(x)|<L. \end{aligned}$$
3. Polynomial tails such that there exist $\gamma , M \ge 1$ and $K \in \mathbb {R}_{+}$ such that
  13 $$\begin{aligned} \sup _{|x|>M} \frac{f(x)}{|x|^{-\gamma }} < K. \end{aligned}$$
4. *Dutchman’s Cap (Bac Mòr) assumption*: (11) implies that there exists $\delta _1>0$ such that for all $x \in B_{\delta _1}(0)$ then $f(x) \le \exp \left( - x^2 \frac{|H|}{4} \right)$; it is assumed that there exists a $\delta _1>\delta _2>0$ such that for all $x \in [-M,M]\backslash B_{\delta _2}(0)$ (where *M* is specified in (13)) then $f(x)\le \exp \left( - \delta _2^2 \frac{|H|}{4} \right)$. Hence, for x∈[-M,M]
  14 $$\begin{aligned} f(x) \le {\left\{ \begin{array}{ll} \exp \left( - x^2 \frac{|H|}{4} \right) , & \text{ if } |x|<\delta _2.\\ \exp \left( - \delta _2^2 \frac{|H|}{4} \right) , & \text{ if } \delta _2\le |x| \le M. \end{array}\right. } \end{aligned}$$

Notice that the tempered distribution in (9) is well approximated by the HAT distributions when the modes become well separated, a regime that naturally arises as the inverse temperature β increases. These assumptions are therefore natural and well suited to the asymptotic regime of interest.

Furthermore, the equivalent procedure in this theoretical analysis setting to the leap point independence sampler proposals is to propose from the Gaussian mixture distribution given by

15 $$\begin{aligned} q_\beta (k,x) = \sum _{j=1}^m w_j \left( \prod _{i=1}^d \left[ \frac{1}{\sigma _{ij}} \phi _\beta \left( \frac{x_i -\mu _{ij}}{\sigma _{ij}}\right) \right] \right) \mathbb {1}_{\{k=j\}}. \nonumber \\ \end{aligned}$$

with $\phi _\beta$ denoting the pdf of a Gaussian of the form $N\left( 0, \left( \beta |H|\right) ^{-1} \right)$.

Moreover, we denote the expected acceptance probability of a Mode Leap Point Independence sampler proposal at the coldest temperature level in this *d*-dimensional setting as

16 $$\begin{aligned} a(d) = \mathbb {E}(A_{IS}(x,y)), \end{aligned}$$

where the independence sampler acceptance probability, $A_{IS}$, is defined in (7) and the expectation is taken with respect to $x\sim \pi _{\beta _{max}}$ as defined in (9) and $y \sim q_{\beta _{max}}$ as defined in (15).

Under the above conditions, we now state the following result about the temperature required to ensure that effective inter-modal mixing can occur.

##### Theorem 5.1

(Dimensionality-scaling for the Coldest Temperature Level)

Assume that the Mode Leap Point Independence sampler with proposal distribution given in (15) is used to target the (d+1)-dimensional target distribution specified by (9), where the marginal iid components *f* satisfy (10),(11), (12), (13) and (14).

If

17 $$\begin{aligned} \beta _{\text {max}} = \ell d. \end{aligned}$$

for some $\ell \in \mathbb {R}_+$ then

$$\begin{aligned} \lim _{d \rightarrow \infty } a(d) = 2 \Phi \left( -\frac{1}{\sqrt{2}} \sqrt{\frac{5h'''(0)^2}{12 \ell (-h''(0))^3}} \right) >0 \end{aligned}$$

where h(x)=logf(x) and Φ is the CDF of a standard Gaussian.

##### Proof

See Appendix A.1. □

##### Remark 2

Theorem 5.1 has been stated within the regime where the asymptotic acceptance probability is positive so that the algorithm can successfully move between modes. However its analysis as seen in the proofs also illustrate why simpler approaches which do not use annealed temperatures are flawed. Suppose we attempt independence sampler moves at the temperature of interest, then

$$\begin{aligned} a(d)\approx 2 \Phi \left( -\sqrt{\frac{d}{2} }\sqrt{\frac{5 h'''(0)^2}{12 (-h''(0))^3}} \right) \end{aligned}$$

which is exponentially small in *d* and therefore in even moderate dimensional problems, this approach won’t work, even in this simplified IID target context.

The limiting acceptance probability in Theorem 5.1 depends explicitly on the local asymmetry through the third derivative of the log-density at the mode. Specifically, the quantity

18 $$\begin{aligned} \gamma \;:=\; -\,\frac{h'''(0)}{\bigl (-h''(0)\bigr )^{3/2}} \end{aligned}$$

represents the *local skewness* of the target density around its mode. This standardized third derivative arises naturally in third-order Laplace and saddlepoint approximations and quantifies departures from local Gaussian behaviour; see, for example, Daniels (1954). From a Bayesian perspective, such skewness occurs naturally in posteriors arising from asymmetric or non-Gaussian priors, for instance a Gamma prior combined with a Poisson likelihood. More generally, local skewness reflects an intrinsic asymmetry of the distribution in a neighbourhood of its mode, whereby probability mass accumulates unevenly on either side of the mode.

Theorem 5.1 shows that, even as the magnitude |γ| increases and the local asymmetry becomes more pronounced, the Mode Leap Point Independence sampler retains a strictly positive asymptotic acceptance probability under linear scaling of the coldest inverse temperature level with dimension. Moreover, any target acceptance probability in (0, 1) can be achieved, under linear scaling $\beta _{\max } = \ell d$, by selecting a sufficiently large constant ℓ, with higher skewness requiring a larger value of this constant while preserving the same linear scaling with dimension. This shows that ALPS can accommodate strong skewness without altering its mixing capabilities.

### Relaxing the regularity conditions

The previous section established the primary result under certain smoothness and regularity conditions on the target density. Yet, it remains an open question whether these assumptions can be loosened. While $C^2$ smoothness at the mode is necessary to ensure weak convergence to a Gaussian distribution, Theorem 5.1 highlights the central role played by the local skewness coefficient (18) at the mode, in determining the performance of the algorithm. In the favourable case where this coefficient vanishes, the local behaviour of the target is effectively symmetric, and the $\beta _{\max }=\mathcal {O}(d)$ scaling becomes more than sufficient, yielding an asymptotic acceptance probability satisfying $\lim _{d\rightarrow \infty } a(d)=1$. This suggests that in the absence of local skewness, a substantially milder annealing regime–potentially sublinear in *d*–may already suffice to maintain a non-vanishing acceptance rate for the Mode Leap Point Independence sampler. This naturally raises the question of what happens when skewness arises not from a smooth third derivative, but instead from piecewise-smooth behaviour, leading to a discontinuity of the third derivative at the mode.

To explore the consequences of relaxing the continuity of the third derivative, we turn once again to a tempered target $\pi _\beta$ in the form (9), relying on the original assumptions for *f* except for the following relaxed ones.

1. Assume that
  19 $$\begin{aligned} \begin{aligned} f \in C^2(\mathbb {R}),\quad f&\in C^5((-\infty ,0)) \cap C^5((0,\infty )), \\ f(0) = 1,\quad f'(0)&=0,\quad \mathop {\mathrm {arg\,max}}\limits f(x)=0, \end{aligned} \end{aligned}$$
  with 0 being the unique global maximum.
2. Furthermore, assume that^1^
  20 $$\begin{aligned}&\forall k \in \{3,4,5\}, \, f^{\left( k \right) }\text { extends continuously both on }\nonumber \\ &\quad (-\infty ,0]\text { and on }[0,\infty ). \end{aligned}$$

We now state the following result showing that, even under these relaxed conditions, the scaling of the inverse temperature required to ensure inter-modal mixing remains unchanged.

#### Theorem 5.2

(Updated dimensionality-scaling for the Coldest Temperature Level) Assume that the Mode Leap Point Independence sampler with proposal distribution given in (15) is used to target the (d+1)-dimensional target distribution specified by (9), where the marginal iid components *f* satisfy (19), (20) (11), (12), (13) and (14).

If

21 $$\begin{aligned} \beta _{\text {max}} = \ell d. \end{aligned}$$

for some $\ell \in \mathbb {R}_+$ then

$$\begin{aligned}&\lim _{d \rightarrow \infty } a(d) \\&= 2 \Phi {\left( - \frac{1}{\sqrt{2}} \sqrt{\frac{15 \left( h^{\prime \prime \prime }{\left( 0_+ \right) }^2 + h^{\prime \prime \prime }{\left( 0_- \right) }^2 \right) - \frac{4}{\pi } \left( h^{\prime \prime \prime }{\left( 0_+ \right) }- h^{\prime \prime \prime }{\left( 0_- \right) } \right) ^2}{72 \ell \left( -h^{\prime \prime }{\left( 0 \right) } \right) ^3}} \right) } > 0 \end{aligned}$$

where h(x)=logf(x) and Φ is the CDF of a standard Gaussian.

#### Proof

See Appendix A.2. □

Theorem 5.2 establishes that discontinuities in the third derivative of the target distribution at its modes do not prevent the Mode Leap Point Independence Sampler from achieving a positive acceptance rate in the asymptotic regime, provided $\beta _{\text {max}}$ scales as $\mathcal {O}(d)$. In this piecewise-smooth setting, the asymptotic acceptance probability decomposes into a symmetric contribution, corresponding to the overall magnitude of local skewness on either side of the mode, and an asymmetric contribution induced by the jump in the third derivative, which captures the mismatch between the left and right local geometries. In particular, when the third derivative is continuous at the mode, the skewness jump vanishes and the result reduces to the smooth-case acceptance of Theorem 5.1.

Such piecewise-smooth behaviour arises in Bayesian settings where the log-posterior is constructed from components that are smooth on each side of the mode but differ in higher-order behaviour, for example due to asymmetric or piecewise-defined priors, truncated or constrained parameter spaces. For a more in-depth investigation of ALPS under less restrictive smoothness and regularity assumptions, readers are referred to Queniat (2024, Sections 3 and 4).

## Computational complexity of ALPS

In the forthcoming Sect. 7, we empirically compare the performance of ALPS with versions of the PT and mode leaping approaches on some challenging multimodal examples. Comparisons of computational efficiency are made between the algorithm’s performances and the results are highly complimentary to ALPS.

With the scaling results presented in Sect. 5 it is possible to theoretically gauge the scalability of ALPS as the dimensionality of the problem grows, at least in stylised problems where explicit calculation and limits are accessible. To be transparent, the problem of finding modes in the full generality of a *d*-dimensional multimodal distribution is well-known to scale exponentially with *d*. This is since modes can, relatively, have exponentially small (in *d*) basins of attraction (e.g., Fong et al. (2019)). However the aim of ALPS is to achieve a robust and efficient method that explores the target distribution highly effectively, conditional upon finding the mode points. So we might hope to improve on this.

Indeed, the question of how the PA component scales with *d* has been addressed in the accompanying paper (Roberts et al. 2022). The approach in Roberts et al. (2022) is to analyse the inverse-temperature process which converges within certain stylised sequences of target density as $d\rightarrow \infty$ to a limiting skew-Brownian motion process. This is attained through appropriate time re-scaling of the process as the dimension grows in the setting where the modes are from an exponential power family. The resulting complexity can be read off from the time re-scaling required to reach an ergodic non-trivial limit.

We refer the reader to Section 5 and in particular Corollaries 2 and 3 of Roberts et al. (2022) for a detailed description of these results (though note the typo in Corollary 3 which should be clear on reading the previous paragraph). For our purposes here, we note that these results suggest that the full version of ALPS has a mixing time that scales as $\mathcal {O}(d)$ and that if the QuanTA transformation-aided swap moves are not deployed (in favour of standard parallel tempering swap moves) then the mixing time scales as $\mathcal {O}(d[\log (d)]^2)$.

It is noteworthy that the scalability of the full version of ALPS is identical in order to that of the random walk Metropolis algorithm in a uni-modal problem. As such, the inter-modal mixing is coming at no extra mixing time cost to now be able to achieve global exploration of the Markov chain. Of course additional cost is incurred computationally and this is now made explicit.

In this setting (and approximately outside it) the optimal temperature schedule is geometrically spaced (Tawn and Roberts 2018). More precisely, for the exponential power family setting analysed in Tawn and Roberts (2018), the optimally spaced inverse-temperatures satisfy

$$ \beta _i = \beta _{i-1}\left( 1+\sqrt{c/d}\right) , $$

for some constant c>0. Thus adjacent inverse-temperatures are separated on a relative scale of order $d^{-1/2}$. Consequently, the number of temperature levels required to reach a cold state, whose inverse-temperature is $\mathcal {O}(d)$ by Theorem 5.1, using optimally spaced inverse-temperatures is $\mathcal {O}(d^{1/2})$. Using the arguments from Roberts et al. (2022), this implies that the mixing time of the inverse-temperature is $\mathcal {O}(d)$.

The leading cost for updating each temperature level per iteration of the algorithm comes from a d×d-dimensional matrix multiplication with a *d*-dimensional column vector in the computation of the mode allocation; this has cost $\mathcal {O}(d^2)$.

Combining the cost per iteration with the mixing time cost results from Roberts et al. (2022) suggest that the scalability of ALPS with dimension, *d* is given by:

$$\begin{aligned} \mathcal {O}(d^3). \end{aligned}$$

This complexity is a substantial improvement on that of standard simulated and parallel tempering, which are demonstrated in Woodard et al. (2009b) and Woodard et al. (2009a) to be no better than exponential in *d*. Of course, this cost for ALPS can only be achieved once the mode points are at least approximately known, and in full generality this is a hard problem in itself.

## Applications of ALPS

This section contains three empirical studies for ALPS. The first is a synthetic example involving a complex, twenty-dimensional target distribution featuring a mixture of highly-skewed components that have differing scales. Due to its synthetic nature we can compare the performance of ALPS against benchmark algorithms, since the number of modes and their locations are known. The second example demonstrates the practicality of ALPS on a real-world data application from an SUR model in economics (Zellner 1962). It is only relatively recently that SUR models have been identified as having issues with multimodality (Drton and Richardson 2004; Sundberg 2010). We show that ALPS performs well on the bimodal example from Drton and Richardson (2004) as well as on a fifteen-dimensional financial dataset (Grunfeld 1958) that was not previously known to exhibit multimodality. Finally, the third example illustrates another application of ALPS on a real-world data problem, with a spectral density model for analytical chemistry (Ritter 1994; Moores et al. 2026). Full R source code for all applications is included in the web-based supporting materials.

### A synthetic 13-modal benchmark distribution

#### Example 1: the 20-dimensional case

The target distribution is given by a mixture of thirteen well-separated, twenty-dimensional skew-normal distributions with heterogeneous scaling of the modes and homogeneous skew. Its density function corresponds to the convex combination

$$\begin{aligned} \pi (x) \propto \sum _{k=1}^{13} \omega _k \prod _{j=1}^{20} \phi \left( \frac{x_j - (\mu _{k})_j }{\sigma _k}\right) \Phi \left( \alpha \frac{x_j - (\mu _{k})_j }{\sigma _k}\right) \end{aligned}$$

where the mixing weights $\omega _k > 0\ \forall k \in \{1,\ldots ,13\}$, skewness parameter α=10, and where $\phi (\cdot )$ and $\Phi (\cdot )$ denote the density and CDF of a standard normal distribution, respectively. The mode locations $\mathbf {\mu }_k$ are distributed in a similar manner to the 13-mode example in Tjelmeland and Hegstad (2001), except in a 20-dimensional hypercube instead of only 2 dimensions. This example has been designed to illustrate the problem with torpid mixing of parallel tempering (Woodard et al. 2009b) due to the heterogeneity of the scales $\sigma _k$ of the mixture components.

We compare the performance of ALPS against the gold standard of optimised parallel tempering and also the version of ALPS which doesn’t anneal, i.e. discovers modes with the hot state mode finder and uses an independence sampler based on the Laplace approximation to the modes. In this section we use the shorthand LAIS (Laplace approximation independence sampler) to refer to this third algorithm. Although the LAIS employs mode-jumping proposals as in Tjelmeland and Hegstad (2001), the latter algorithm performs two optimisation steps at each iteration to find the nearest modes, rather than running an Exploration Component as a parallel chain. This reduces the computational cost of LAIS considerably, without changing its convergence properties. The strong skewness of the mixture components will prove to be critical for the acceptance rate of LAIS, since the error in the Laplace approximation increases with dimension, as explained by Remark 2 in Sect. 5.1.

The basic configurations of the three algorithms, the different levels of tuning required, the sample output from the chains, and the comparison of elapsed run times are described as follows.

The LAIS only uses a single temperature level which is the target level. For ALPS, 7 temperature levels were used for the annealing part of the algorithm with a coldest inverse temperature $\beta _{\text {max}}= 4096$ and a cooling schedule given by powers in the vector (1, 4, 16, 64, 256, 1024, 4096). The maximum level of cooling was excessively cold as non-degenerate acceptances for mode jumping moves were achieved at warmer temperatures (in agreement with the results of Sect. 5). However, because mixing across temperatures proves extremely quick at colder temperatures and evaluating the synthetic target is inexpensive, it is possible to use much lower temperatures to get higher acceptance rates for the mode-jumping moves. In this example the mode jumping acceptance rates were around 0.85.

In contrast, PT required 23 temperature levels which were geometrically spaced with common ratio of 0.6. This gave the suggested optimal temperature swap rates of 0.234 between consecutive temperature level pairs along with a temperature that appeared hot enough to explore the entire state space. Note that the minimum power $\beta _{min} = 0.6^{22} \approx 1.3 \times 10^{-5}$ used for PT is slightly hotter than the inverse temperature used for mode-finding in ALPS, $\beta _{\text {hot}} = 2.5 \times 10^{-5}$, in order to make a fair comparison between the two algorithms. Even so, the PT algorithm fails to find all 13 modes even after 500,000 iterations. This is due to the differences in scale $\sigma _k$ between the mixture components, as predicted by Woodard et al. (2009a). Due to torpid mixing, it could take several million more iterations for PT to converge.

In all three algorithms random walk Metropolis was utilised for the within temperature moves; in the case of ALPS and LAIS the random walk Metropolis moves used pre-conditioned covariance structure informed by the covariance matrix estimated at the local mode point by the Laplace approximation. In all cases the within temperature moves were tuned to have the suggested optimal acceptance rate of 0.234.

The most difficult aspect of tuning ALPS was with respect to the Exploration Component for mode-finding. This was also highlighted in the real-data example in the following section. As mentioned previously, finding “narrow modes” is never guaranteed in the finite run of the algorithm if their location is unknown (Fong et al. 2019). In both LAIS and ALPS where mode-finding is required, it was found that an inverse temperature of $\beta _{\text {hot}} = 0.000025$ worked very well and rapidly found the modes; typically within a few thousand iterations of the algorithm. However, in this example the number of modes was known and so the mode finding could be optimised for ALPS and LAIS; something not possible in a real data example where one may continue mode searching throughout the duration or even doing some pre-computation dedicated to mode searches (as was the case in the following real-data example in Sect. 7.2).

Each of the three algorithms was run long enough to generate 500,000 samples at the target temperature (including the “burn-in” samples from the transient phase). For the purpose of comparison, the chains were all initiated from the first mode location, $\mu _1$ in each instance. The EC took less than 3,000 iterations to find all 13 modes. Since this chain only searches for modes at every fourth iteration, this corresponds to 12,000 iterations of ALPS or LAIS. Based on the trace plots, we discarded the first 70,000 iterations as burn-in. However, note that ALPS is the only algorithm that has actually converged to the target distribution, $\pi (\cdot )$. This means that the burn-in required for LAIS or PT is at least 500,000 iterations and possibly much more. The elapsed run time for ALPS was 3.2 h, while it took 2.5 h for LAIS and 2.3 h for PT. The run time of LAIS was dominated by the computational cost of the EC chain, even though this was a small proportion of the total number of iterations. This is due to the large number of likelihood evaluations required for each optimisation step. The difference between ALPS and LAIS is due to the multiple cold temperature levels in ALPS. Although PT has the fastest run time of the 3 algorithms for a fixed number of iterations, it should again be stressed that PT has not yet converged at that point.

**Fig. 2:**
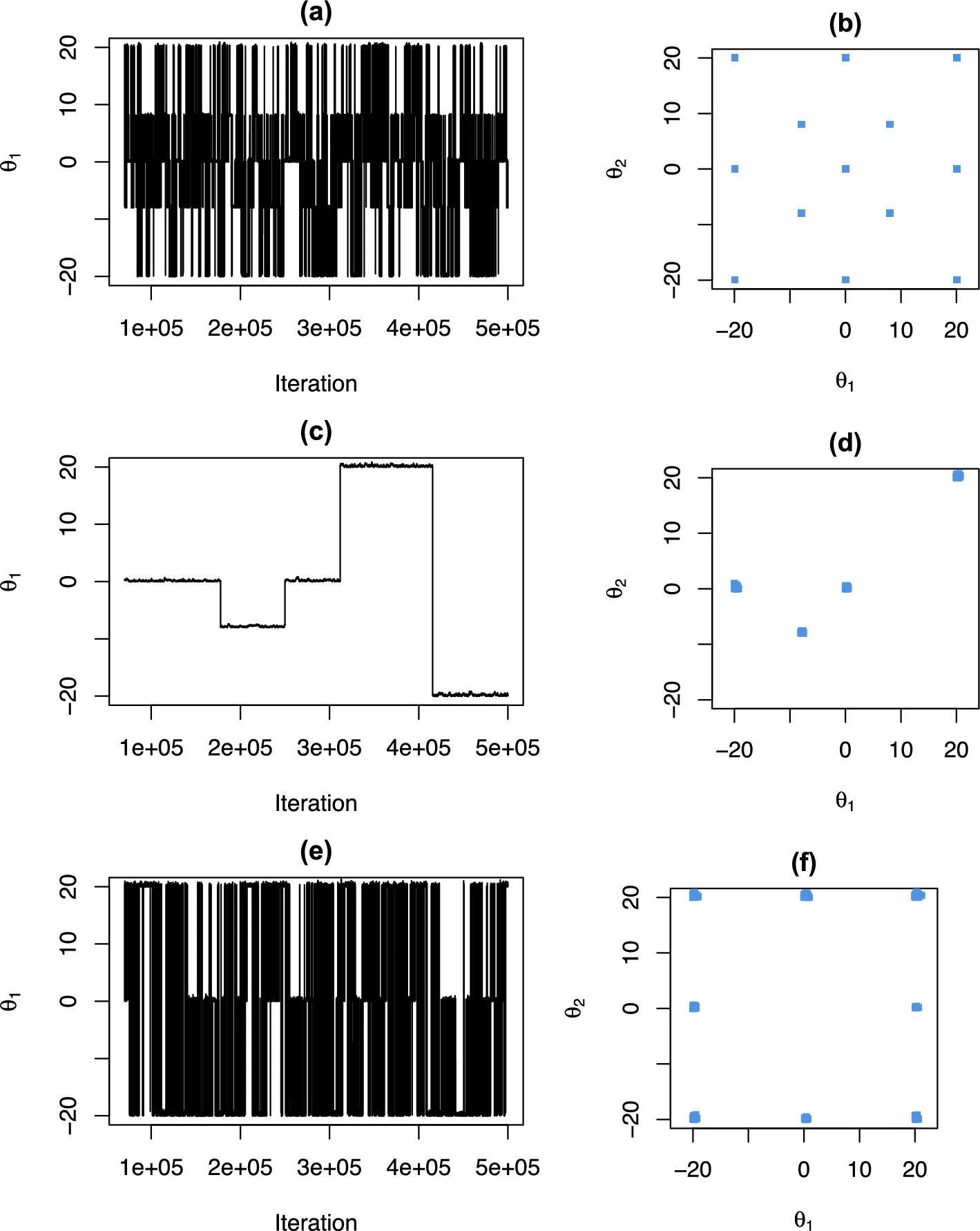
For the algorithms ALPS (top), LAIS (middle) and PT (bottom) respectively, the trace plots (left) of the first component of the Markov chain as well as marginal density plots (right) of the first 2 components at the target temperature, β=1. It can be seen from the trace plot (**a**) that ALPS mixes well and (**b**) shows that it visits all 13 modes. In contrast, (**c**) shows that LAIS very rarely jumps between modes and (**d**) only visits 4 of the modes. From (**e**) PT appears to be mixing well, but (f) shows that it has only visited 8 modes

Figure 2 shows trace plots (on the left side) for the first component of the Markov chain at β=1 for each of the three algorithms after a burn-in is removed. Corresponding marginal density plots for the first two components are shown alongside. A successful outcome would be a plot where the trace line jumps between the modes of the marginal component, centred at values -20, -8, 0, 8 and 20, as shown in (a) for ALPS. Although d=20, the first two coordinates of the 13 modes are unique, thus the mode locations can be distinguished in the marginal plots shown on the right side of Fig. 2. This enables us to verify whether the chain is actually visiting all 13 modes. For example, (b) shows ALPS was the only algorithm to successfully jump between all of the mode points.

Even though LAIS could propose jumps between all 13 modes, the empirical acceptance rate at the target temperature β=1 is around $1.4 \times 10^{-5}$, which means only 5 proposals are accepted out of 350,000 attempts (one of these 5 jumps is during the burn-in phase). Due to the skewness of the mixture components, the error in the Laplace approximation is too large for LAIS to work effectively. This is evident in (c), while (d) shows that LAIS has only visited 4 of the modes. In contrast, the acceptance rate for mode-jumping proposals in ALPS was 0.85 at the coldest temperature level.

The trace plot for PT (e) appears to show that it is mixing well, but actually it is only visiting modes at -20, 0 or 20, not at -8 or 8. This demonstrates the danger of relying on one-dimensional trace plots to assess convergence in high-dimensional problems. In a real data example where the true modes were unknown, we might erroneously conclude that PT has successfully converged. We have artificially constructed this example so that we can see all of the modes in the two-dimensional marginal plot, (f). Here it is evident that PT has only visited the 8 modes with $\sigma _k = 0.3$ and has been unable to find the 5 smaller modes with $\sigma _k = 0.2$. Although the difference in scale might appear small, when d=20 the relative volume of the domain of attraction is approximately $0.2^{20}$ or $10^{-14}$ for the smaller components and $0.3^{20}$ or $3.5 \times 10^{-11}$ for the larger ones, a difference of several orders of magnitude.

In addition to the simulation study presented here, we have also included a performance comparison of ALPS, LAIS and PT across 10 repeated runs of each algorithm in Appendix D. This example has 4 modes instead of 13 and we run each algorithm for 200,000 iterations, discarding the first 20,000 as burn-in.

#### Scaling with dimension at the cold level

We now empirically validate the dimensional scaling result of Theorem 5.1 for the coldest temperature level using the synthetic multimodal example introduced above. The aim of this experiment is to assess whether the asymptotic acceptance rate derived in Theorem 5.1 is verified in high-dimensional settings.

Given a prescribed target acceptance rate a∈(0,1) at the cold level, Theorem 5.1 implies that the linear scaling

22 $$\begin{aligned} \beta _{\text {max}} = \ell (a)\, d \end{aligned}$$

yields a non-degenerate asymptotic acceptance rate. Inverting the limiting expression in Theorem 5.1, the constant ℓ(a) in (22) is given explicitly by

23 $$\begin{aligned} \ell (a) = \frac{5 h'''(0)^2}{24 \, (-h''(0))^3 \, \left[ \Phi ^{-1}(a/2)\right] ^2 }, \end{aligned}$$

where h(x)=logf(x) and Φ denotes the standard Gaussian distribution function. In this synthetic example, the function *f* is fully specified, so that (23) can be computed explicitly.

Using this expression, we compute $\beta _{\text {max}}$ for increasing dimensions and apply the Mode Leaping Independence Sampler to the same synthetic mixture model, keeping the number of modes fixed. In this experiment, the skewness parameter is set to α=5.0. For each dimension, we report the empirical acceptance rate of the mode-jumping proposals.

**Fig. 3:**
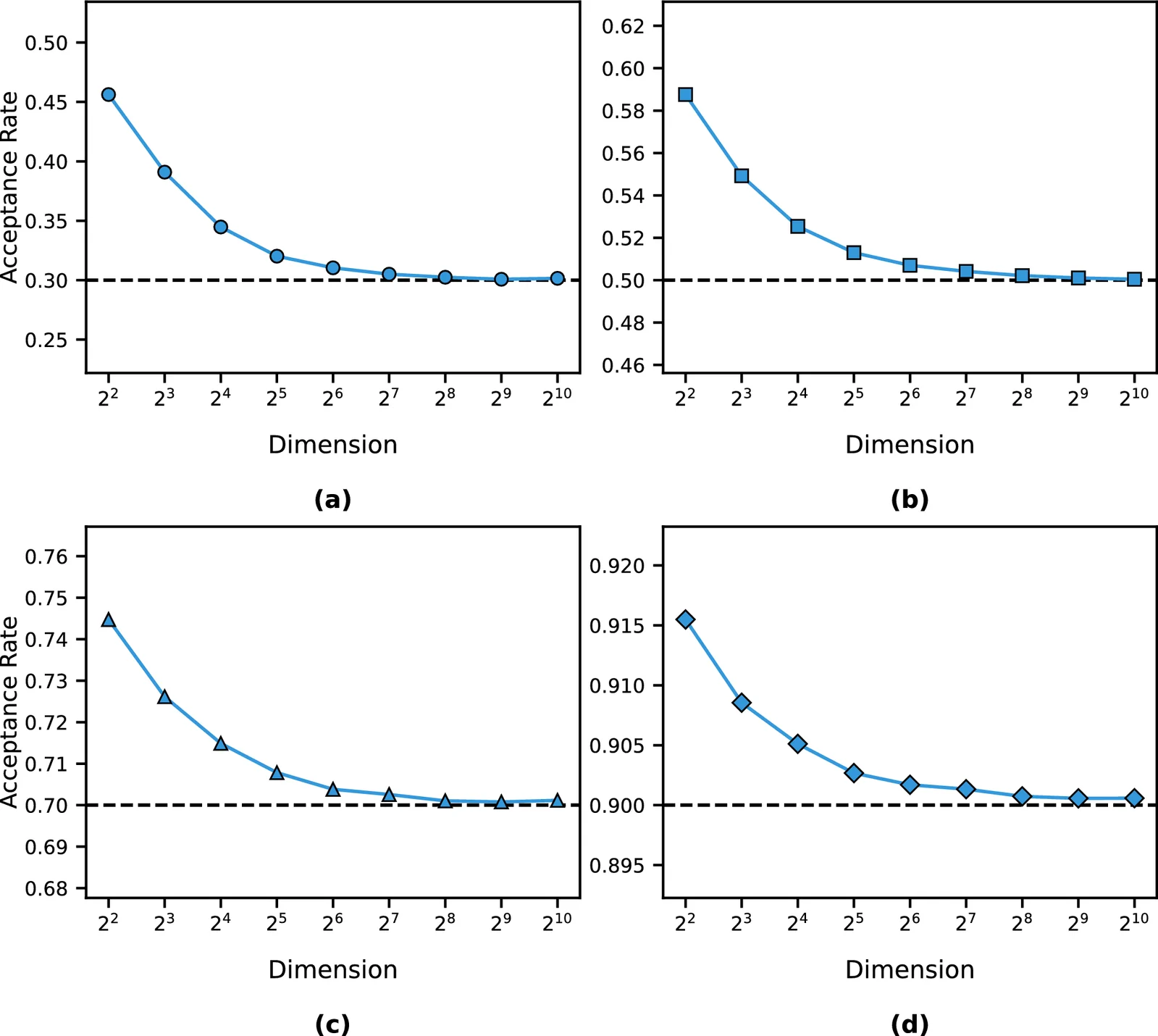
Empirical acceptance rates of the mode-jumping proposals at the coldest temperature level as a function of dimension, averaged over 128 runs of 16384 iterations. For each dimension *d*, the cold inverse temperature is set to $\beta _{\text {max}} = \ell (a)\, d$, where ℓ(a) is computed according to (23). Target acceptance rates *a* are set to (**a**) 0.3, (**b**) 0.5, (**c**) 0.7 and (**d**) 0.9

The results are shown in Fig. 3. As the dimension increases, the empirical acceptance rates approach the prescribed target value, providing empirical support for the linear scaling $\beta _{\text {max}} = \ell (a)\, d$, thereby verifying the asymptotic result stated in Theorem 5.1.

### Example 2: seemingly-unrelated regression model

Multi-response models (Searle and Khuri (2017), ch. 14) arise from datasets that have more than one dependent variable, where these variables are correlated with each other. An important example is Seemingly-Unrelated Regression (SUR or SURE), which was introduced by Zellner (1962). A wide variety of different SUR models are described by Srivastava and Giles (1987) and the review of Fiebig (2003). These models involve a system of *M* linear regression equations, one for each response, m=1,⋯,M:

24 $$\begin{aligned} \vec {y}_m = \textbf{X}_m \mathbf {\theta }_m + \mathbf {\epsilon }_m, \end{aligned}$$

where $\vec {y}_m$ is a column vector of $N_m$ observations for the *m*th response, $\mathbf {\theta }_m$ is a vector of $J_m$ regression coefficients, $\textbf{X}_m$ is an $N_m \times J_m$ design matrix of covariates, and $\mathbf {\epsilon }_m$ is a vector of residual errors. For notational convenience, we assume that $N_1 = N_2 = \dots = N_M = N$ and likewise that $J_1 = J_2 = \dots = J_M = J$, although in full generality that is not always the case. See Appendix E for further introductory information on the SUR model.

Drton and Richardson (2004) provided a specific example of a dataset where the SUR model exhibits two local modes, which we will examine in more detail in the following section.

#### Bimodal example

In the simplest, bivariate case with M=2 responses and only J=1 covariates per response, the model can be written as:

$$\left[ \begin{array}{c} \vec {y}_1 \\ \vec {y}_2 \end{array}\right] = \left[ \begin{array}{cc} \vec {x}_1 & \vec {0} \\ \vec {0} & \vec {x}_2 \end{array}\right] \left[ \begin{array}{c} \theta _1 \\ \theta _2 \end{array}\right] + \left[ \begin{array}{c} \mathbf {\epsilon }_1 \\ \mathbf {\epsilon }_2 \end{array}\right] . $$

**Fig. 4:**
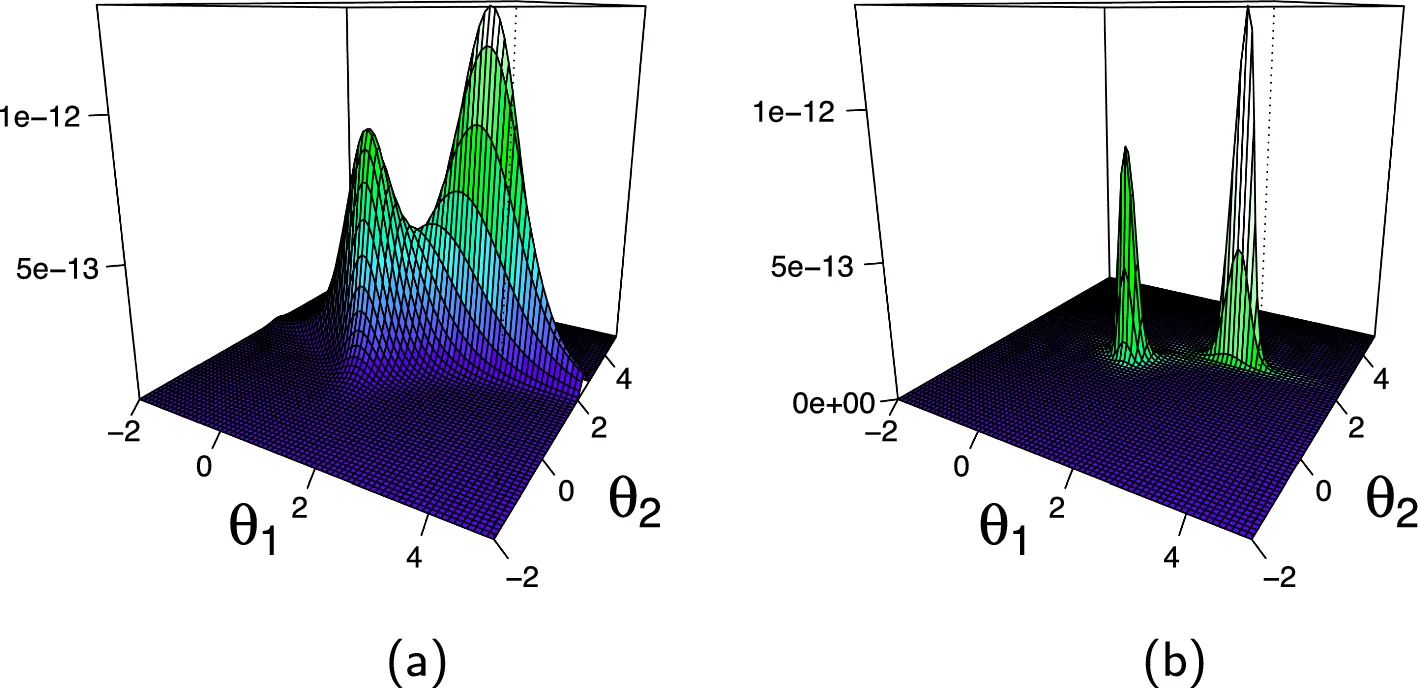
(**a**) 3D perspective plot of the profile likelihood over the covariance matrix for the bivariate SUR model, adapted from Drton and Richardson (2004, Fig. 1); (**b**) Annealed likelihood at the coldest temperature level used in ALPS

Drton and Richardson (2004) featured an example dataset with N=8 observations. The profile likelihood, derived in Appendix E, is illustrated in Fig. 4. There are two modes at $\boldsymbol{\theta }=$ (0.78, 1.54) and (2.76, 2.50) and a saddle point at (1.62, 2.03). The iterative algorithm is initialised at the ordinary least squares estimate, $\hat{\boldsymbol{\theta }} =$ (1.25, 1.78). Using the R package ‘systemfit’ (Henningsen and Hamann 2007) with a tolerance level of $10^{-6}$, the algorithm of Zellner (1962) converges to the first mode after 25 iterations. However, the profile log-likelihood at this mode is −27.72, whereas at the other mode it is −27.35. This algorithm has therefore failed to find the global maximum.

**Fig. 5:**
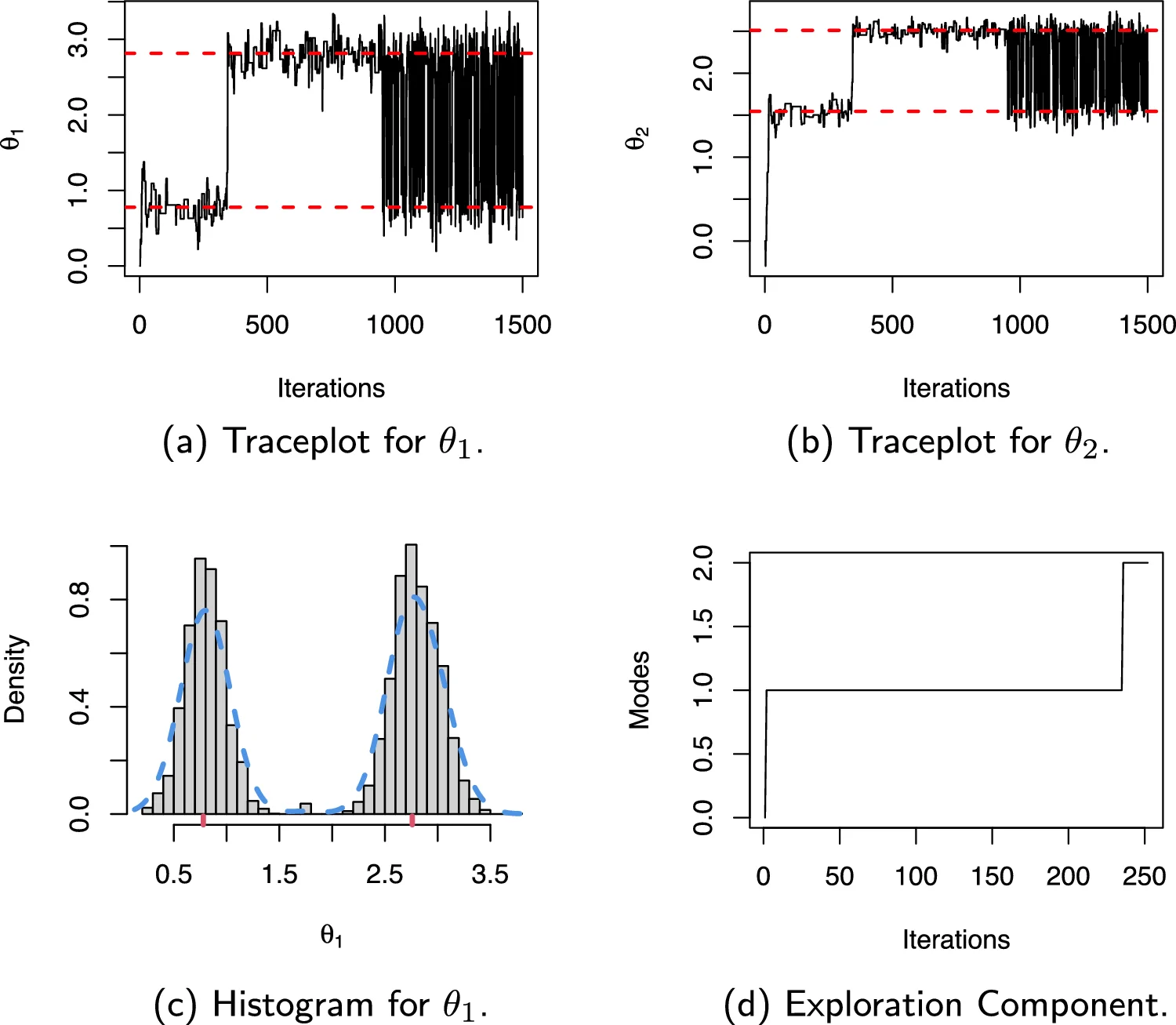
Results for ALPS at the target temperature with the bivariate SUR model from Drton and Richardson (2004). We see that upon discovery of the two modes, the ALPS-PA successfully enables inter-modal exploration between the two modes, whose locations marginally are indicated by the red dashed lines. Note that the ratio of iterations between the hot-state mode finder and the other chains is 1:4, so 236 iterations of the Exploration Component correspond to 944 iterations of the within-temperature chains. This example highlights that before discovery of both the modes the within moves fail to explore the state space successfully

We run ALPS using the same profile log-likelihood function, with $\beta _{\text {max}} = 10$ and a geometric cooling schedule composed of three inverse-temperature levels $(1, \sqrt{10}, 10)$ as well as the hot state 0.5. The Exploration Component discovers both modes after 236 iterations, as shown in Fig. 5 (d), and ALPS takes 2.6 s for 10,000 iterations in total. The MCMC trace plots for the two parameters at the coldest temperature are shown in Figs. 5 (a) and 5 (b), respectively, and a histogram for $\theta _1$ is shown in Fig. 5 (c). The jump rate for mode-hopping proposals was 0.9 at the coldest temperature, with acceptance rates of 0.067 for the EC, 0.265 for within-temperature moves at the intermediate temperature and 0.331 at the target temperature. The temperature-swap acceptance rates between consecutive levels were (0.508, 0.847).

#### Investment demand

Zellner (1962) illustrated his method using an investment equation with J=3 terms,

$$ \vec {y}_m = \vec {x}_{1,m} \theta _{1,m} + \vec {x}_{2,m} \theta _{2,m} + \vec {x}_{3,m} \theta _{3,m} + \mathbf {\epsilon }_m, $$

where $y_{i,m}$ is the gross investment by firm *m* during the *i*th year, $\vec {x}_{1,m}$ is a vector of ones (so that $\theta _{1,m}$ is a firm-specific intercept), $\vec {x}_{2,m}$ is the market value of the firm, and $\vec {x}_{3,m}$ is its capital stock. The dataset of U.S. manufacturing firms was originally published by Grunfeld (1958) and has since received considerable attention in the econometrics literature, as reviewed by Kleiber and Zeileis (2010). We will consider the first N=15 years of data, from 1935 to 1949, for M=5 firms (General Motors, Chrysler, General Electric, Westinghouse, and US Steel), so the parameter space has 15 dimensions in total. These data are available in the R package systemfit (Henningsen and Hamann 2007).

The iterative algorithm of Zellner (1962) took 52 iterations to converge to a local mode, with a sum of squared residuals (SSR) of 216,943 and profile log-likelihood of −263.7. However, Drton and Richardson (2004) suggests that this problem exhibits multimodality and so we attempt to use ALPS to sample from the posterior distribution.

**Fig. 6:**
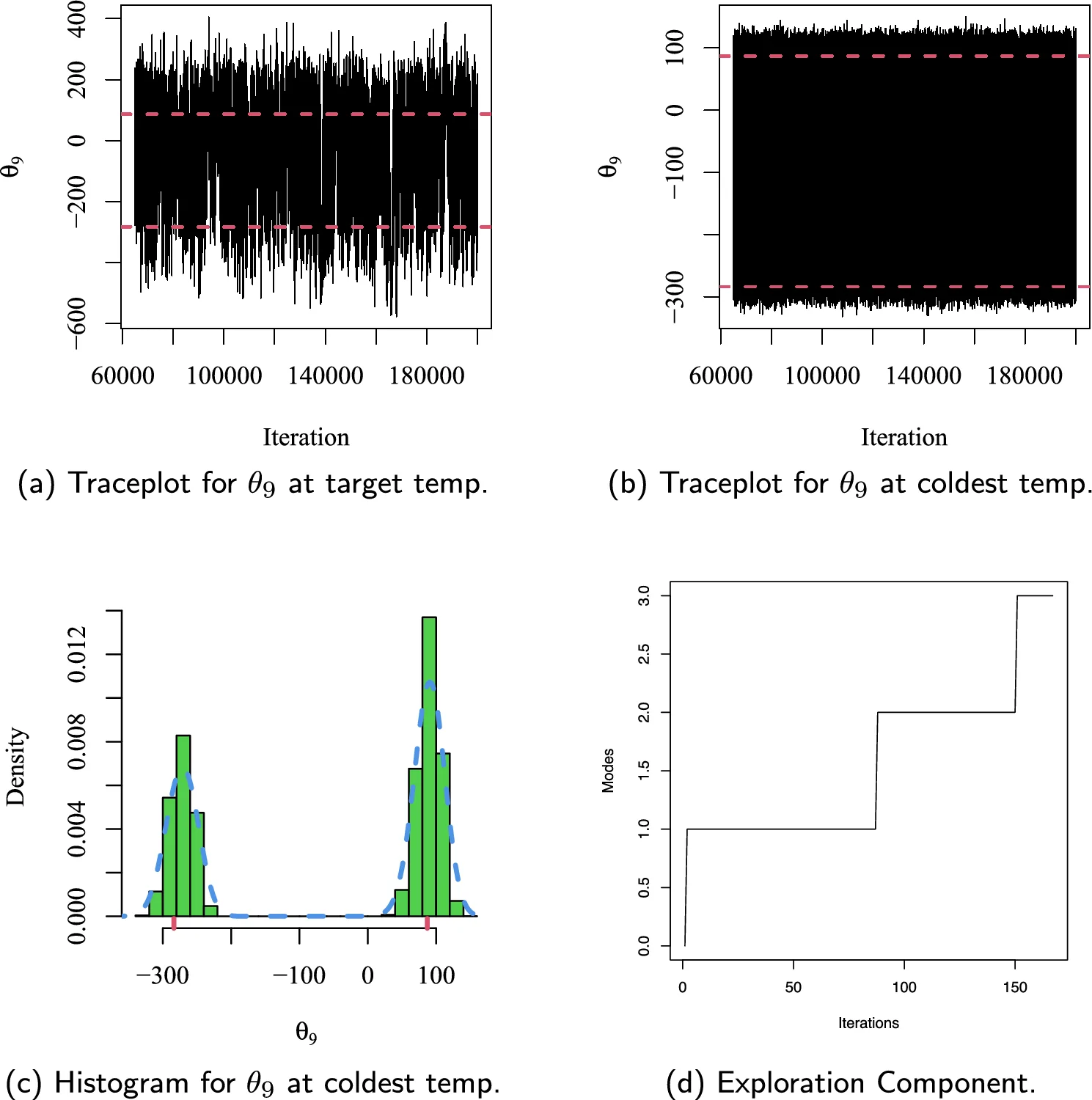
Results for ALPS with the SUR dataset from Grunfeld (1958) with N=15 years of observations for M=5 manufacturing firms, with J=3 covariates per firm. The red, dashed lines show the locations of two of the modes (the third mode has negligible probability mass). The EC takes a little over 150 iterations to find the modes as shown in (**d**). (**b**), (**c**) show that in the one-dimensional marginal ALPS is making successful intermodal jumps at the coldest inverse temperature $\beta _{max}= 32$, while (**a**) confirms that this mixing information is successfully transferred to the target level

Using the EC, three modes were discovered by the EC chain as shown in Fig. 6 (d), although the estimated weight suggests the third mode has negligible probability mass. The first mode discovered by the EC part of ALPS matches the estimate from systemfit, while the other two modes have profile log-likelihoods of −264.9 and −329.1, respectively.

This problem is particularly ill-posed due to parameter unidentifiability (which leads to the multimodality in the posterior). In this problem, despite attempts to reparameterize, there remain issues with the modes of the posterior distribution being located on long, thin ridges that seem to decay with significantly heavier tails than Gaussian. This means that to obtain a good Laplace approximation to the mode the ALPS procedure would need to use very large inverse-temperature values. However, when this was attempted it led to severe computational issues with regards to machine precision, likely due to the condition numbers of the Hessians at the mode points, illustrating the ill-posed nature of the problem. To overcome this a truncated version of the annealed HAT targets was utilised for annealed temperatures with β>1. These are a small modification to the HAT targets and they are defined and discussed in detail in Appendix B.2.

We ran ALPS with four inverse temperature-levels (1, 4, 16, 32). The MCMC trace plot for the ninth parameter (General Electric capital stock) at the coldest temperature is shown in Fig. 6 (b). Traceplots for the marginal chains 15 parameters at the target temperature β=1 and the coldest inverse temperature $\beta _{max}= 32$ are provided in Appendix E.1. The jump rate for mode-hopping proposals was 0.517. The temperature-swap acceptance rates between consecutive levels were (0.264, 0.560, 0.681). For robustness of within-temperature moves, we used a mixture of pre-conditioned and standard random walk moves with acceptance probabilities between 0.37 and 0.42. The ALPS-PA component generating 200,000 samples took under 40 s.

ALPS is not ideally suited to this example. The modes are not especially well-separated and the modes lie on narrow ridges which lead to computational instability. Additional Hessian regularization, for example using the SoftAbs map of Betancourt (2013), could improve numerical stability in this setting. Nevertheless, the fact that the algorithm still draws samples from multiple modes suggests that the approach remains robust in settings where reliable local Laplace approximations are challenging to obtain.

### Example 3: spectral density model

Ritter (1994) was the first to introduce a Bayesian approach for peak decomposition of electron spectroscopy. This has become a popular method in analytical chemistry for many different applications, including X-ray and γ-ray spectroscopy (van Dyk et al. 2001) and mass spectrometry (Wang et al. 2008). In this example, we will use ALPS to fit a peak decomposition model for Raman spectroscopy (Moores et al. 2026). The model represents spectroscopic data $\vec {y} \in \mathbb {R}^n$ as a conical combination of *M* basis functions:

25 $$\begin{aligned} \vec {y} = \sum _{m=1}^M \alpha _m f_m(\vec {x};\, \ell _m, \psi _m) + \mathbf {\epsilon }, \end{aligned}$$

where the observed spectrum has been discretised at wavenumbers $x_1,\dots ,x_n \in \mathcal {X} \subset \mathbb {R}$. In this example $\mathcal {X}$ is the interval from 100 to 600 $\hbox {cm}^{-1}$ and the n=101 wavenumbers are evenly spaced, 5 $\hbox {cm}^{-1}$ apart. We assume additive *i.i.d.* Gaussian noise, $\epsilon _i \sim \mathcal {N}(0, \sigma ^2)$. Each of the peaks is described by three parameters: its amplitude $\alpha _m > 0$, location $\ell _m \in \mathcal {X}$, and scale $\psi _m > 0$. The dimension of the parameter space is therefore 3*M*. We assume that each spectral density function $f_m(\cdot )$ is either Lorentzian,

26 $$\begin{aligned} f_L(x;\, \ell _m, \psi _m) \propto \frac{\psi _m^2}{(x - \ell _m)^2 + \psi _m^2}, \end{aligned}$$

or Gaussian,

27 $$\begin{aligned} f_G(x;\, \ell _m, \psi _m) \propto \exp \left\{ - \frac{(x - \ell _m)^2}{2 \psi _m^2} \right\} . \end{aligned}$$

Our choice of basis functions $f_L(\cdot )$ and $f_G(\cdot )$ is motivated by the physical data-generating process of Raman spectroscopy, as described by Diem (2015) and Moores et al. (2026).

**Fig. 7:**
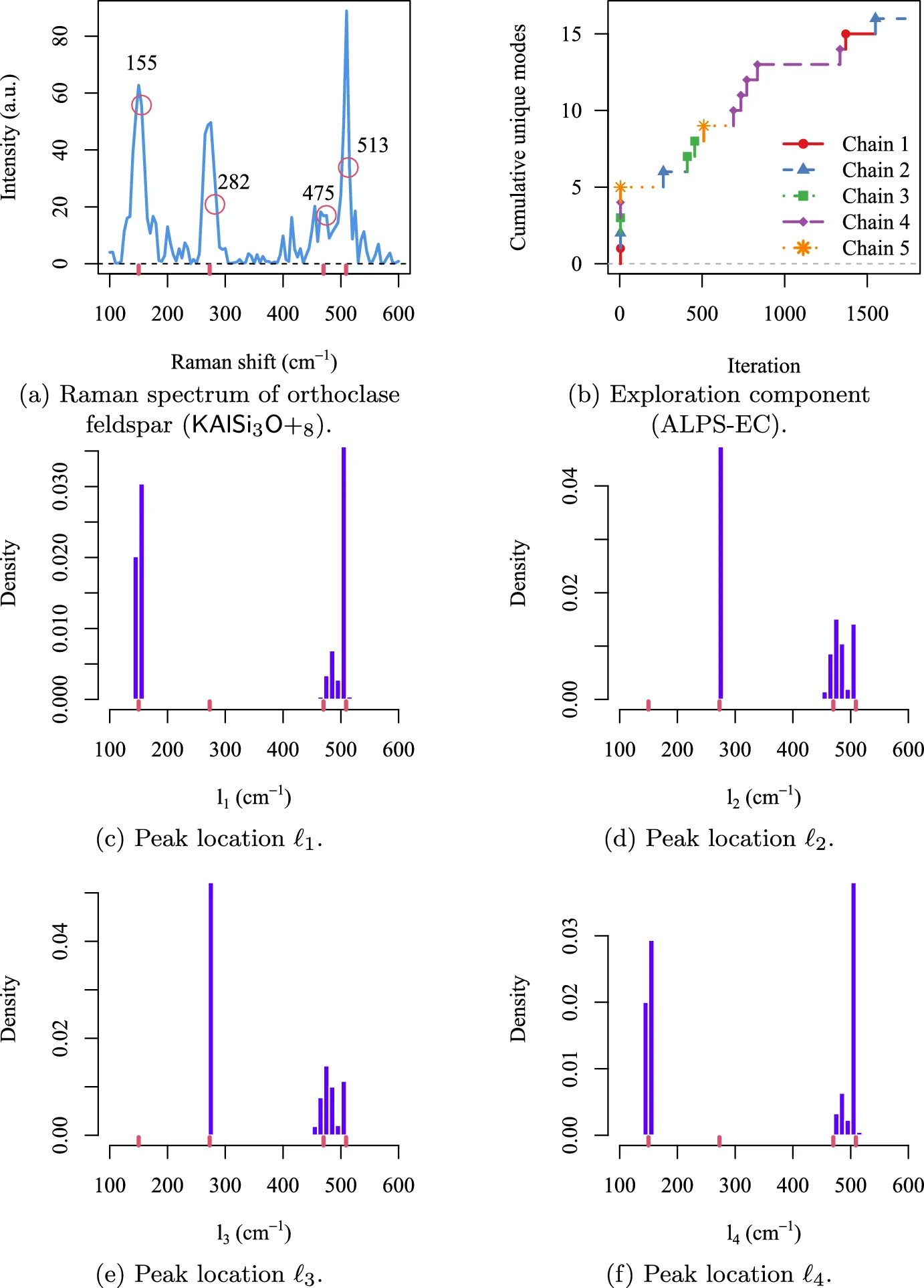
Raman spectrum of orthoclase (**a**) showing the 4 peak locations reported by Freeman et al. (2008; **b**) the 16 modes found by the parallel ALPS-EC; and (**c**)–(**f**) histograms of the ALPS-PA output for the peak location parameters at the target temperature β=1

For this example, $\vec {y}$ is a Raman spectrum of orthoclase feldspar ($\hbox {KAlSi}_3$ $\hbox {O}_8$) shown in Fig. 7 (a). There are M=4 peaks in this spectrum, two Gaussian and two Lorentzian. The peak locations $\ell _1,\dots ,\ell _4$ are slightly different to those reported by Freeman et al. (2008) and therefore need to be inferred from the data. The scale and amplitude parameters of these peaks are also unknown. The other peaks reported by Freeman et al. (2008) are below the limit of detection in this sample.

To fit the model in Eq. (25) with ALPS, we transform the parameters to the unconstrained space $\mathbb {R}^d$ using bijective functions: $a_m = \log \{\alpha _m\}$; $s_m = \log \{\psi _m\}$; and

28 $$\begin{aligned} l_m = \text {logit}\left\{ \frac{\ell _m - x_1}{x_n - x_1}\right\} , \end{aligned}$$

where in this example the minimum wavenumber $x_1 = 100$ $\hbox {cm}^{-1}$ and the maximum $x_n = 600$ $\hbox {cm}^{-1}$. This results in a parameter vector $\mathbf {\theta }= (l_1, s_1, a_1, \dots , l_M, s_M, a_M)^\top \in \mathbb {R}^{12}$.

**Table 1:** Modes found by ALPS-EC for the spectral density model. Weight is a Laplace approximation to the relative weight of each mode, which involves the log-likelihood $\log (\mathcal {L})$ and the log-determinant $\log (|\Sigma |))$. $\ell _1,\dots ,\ell _4$ are the location parameters of the four peaks

	Weight	$\log (\mathcal {L})$	$\log (|\Sigma |)$	$\ell _1$	$\ell _2$	$\ell _3$	$\ell _4$
1	0.319	−285.2	−74.54	150	272	477	509
2	0.120	−285.2	−76.48	150	272	509	484
3	0.001	−290.0	−76.48	150	486	509	272
4	0.001	−290.0	−76.48	150	509	486	272
5	0.319	−285.2	−74.54	150	477	272	509
6	0.120	−285.2	−76.48	484	272	509	150
7	0.120	−285.2	−76.48	150	509	272	484

We ran 8 parallel independent chains of ALPS-EC, using the R package future (Bengtsson 2021) to run each chain in its own concurrent thread. These chains took 1550 iterations to find 16 unique modes, as shown in Fig. 7 (b). Running on a computer with 8 CPU cores, it took less than 110 s.

However, only 7 of these modes appear to have non-negligible mass and these are presented in Table 1 along with their estimated wights via Laplace approximation.

In a typical label-switching regime such as a mixture model (Jasra et al. 2005) we would expect the likelihood to be invariant to permutation of the labels, but for this model it is not necessarily the case. That is because of the different spectral density functions $f_L(\cdot )$ (26) and $f_G(\cdot )$ (27). For example, $\ell _1$ and $\ell _4$ are both Lorentzian peaks, so those peak locations can be swapped without affecting the likelihood, as in modes 6 and 7. However, if $\ell _3$ and $\ell _4$ are swapped, as in modes 1 and 2, that does change the relative weight.

To aid mode finding in this setting one could further exploit permutation proposals (Frühwirth-Schnatter 2001) to jump between modes, but this would be problem-specific: the user would need to encode the symmetric structure of the parameter space, which might not be known in advance.

ALPS-PA took 3.6 min for 80,000 iterations with six inverse-temperature levels (1, 1.6, 2.56, 4.096, 6.55, 10.45). The jump rate for mode-hopping proposals was 0.885 at the coldest inverse temperature $\beta _{\max } = 10.45$. For robustness, we used a mixture of pre-conditioned and standard random walk moves with acceptance probabilities between 0.22 and 0.36. The temperature-swapping acceptance rates were 0.412 between the first two temperatures and 0.969 between the coldest temperatures.

Marginal histograms of the location parameters $\ell _1,\dots ,\ell _4$ at the target temperature are shown in Figs. 7(c)–(f). The long-range dependence in the peak at 150 $\hbox {cm}^{-1}$, characteristic of Lorentzian line shape, is evident from the high probability of $\ell _1$ and $\ell _4$. Similarly, the Gaussian line shape of the peak at 273 $\hbox {cm}^{-1}$ is clearly identified by the model. These first two peaks are well-separated from the others, whereas the peaks at 470 $\hbox {cm}^{-1}$ and 509 $\hbox {cm}^{-1}$ are overlapping with each other. In analytical chemistry, the peak at 470 $\hbox {cm}^{-1}$ is known as a shoulder. Reliable inference for peaks like this, which are common in Raman spectroscopy, is a difficult problem. All four location parameters assign some probability to this peak, since the data are too noisy to clearly identify whether it is Gaussian or Lorentzian. There is also some ambiguity in the line shape of the peak at 509 $\hbox {cm}^{-1}$, but the model assigns higher probability for Lorentzian than Gaussian.

## Conclusion and further work

This paper introduces a novel algorithm, ALPS, that is designed to sample effectively and scalably from multimodal distributions that have support on $\mathbb {R}^d$ and are $C^2$ smooth. The main methodological contribution of this paper is that this problem can be solved by (localised) annealing rather than tempering and that this new approach mitigates many of the issues due to mode heterogeneity that plague traditional methods for these problems. Also, ALPS exploits recent advances in the literature that both enable the algorithm to be stable in high dimensions as well as providing substantially accelerated performance.

The accompanying theoretical contribution of the paper shows that the coldest temperature required for the procedure will be $\mathcal {O}(d)$ under suitable regularity conditions. This gives both guidance on tuning the algorithm and demonstrates polynomial computational complexity cost of $\mathcal {O}(d^3)$.

We have applied ALPS to two real-world examples of multimodal distributions, a Seemingly-Unrelated Regression (SUR) model of investment demand (Zellner 1962) and a peak decomposition model of Raman spectroscopy (Moores et al. 2026). There are many more potential applications for this algorithm to sample from continuous distributions where multimodality is known or suspected to be present, such as dynamical models of biological systems (Ballnus et al. 2017), differential equation models (O’brien et al. 2026), and other curved exponential families (Sundberg 2010).

There are a number of further considerations regarding adding robustness and practicality to the ALPS procedure. Many of the ideas for exploration are already alluded to in the paper: improving the effectiveness of the EC part by running more than one exploration chain and having multiple temperature levels; parallel implementation of the PA and EC parts of ALPS; and introducing weightings to regions to stabilise the inter-modal masses when the HAT targets don’t sufficiently preserve the weights. Another possibility would be to incorporate the cold state level into a simulated tempering framework, interpolating between the cold Laplace approximation and the target distribution, and serving, per se, as a prior or reference distribution as in Surjanovic et al. (2022). This would allow perfect regeneration of the Markov chain at each visit to the cold level thanks to the i.i.d. sampling of the Laplace approximation. Adapting the ideas of ALPS to facilitate an automatic and scalable reversible jump MCMC algorithm is the immediate focus for further work.

## Supplementary Information

Below is the link to the electronic supplementary material.

Supplementary file 1 (pdf 9415 KB)

## Data Availability

No datasets were generated or analysed during the current study.

## Footnotes

1 We assume continuity at 0 up to the second derivative. Higher-order derivatives are assumed continuous only on $\mathbb {R}_\pm$; the corresponding one-sided limits at 0 are allowed to differ.

## References

[CR1] Aitken, A.C.: Iv-on least squares and linear combination of observations. Proc. R. Soc. Edinb. **55**, 42–48 (1936). 10.1017/s0370164600014346

[CR2] Aarts, E., Korst, J.: Simulated Annealing and Boltzmann Machines: A Stochastic Approach to Combinatorial Optimization and Neural Computing. Wiley, USA (1989)

[CR3] Atchadé, Y.F., Liu, J.S.: The Wang-Landau algorithm in general state spaces: Applications and convergence analysis. Statistica Sinica **20**(1), 209–233 (2010) arXiv:2430.8988

[CR4] Atchadé, Y.F., Roberts, G.O., Rosenthal, J.S.: Towards optimal scaling of metropolis-coupled Markov chain Monte Carlo. Stat. Comput. **21**(4), 555–568 (2011). 10.1007/s11222-010-9192-1

[CR5] Bengtsson, H.: A unifying framework for parallel and distributed processing in R using futures. R J. **13**(2), 208–227 (2021). 10.32614/RJ-2021-048

[CR6] Betancourt, M.: A general metric for Riemannian manifold Hamiltonian Monte Carlo, pp. 327–334. Springer, Berlin, Germany (2013). 10.1007/978-3-642-40020-9_35

[CR7] Ballnus, B., Hug, S., Hatz, K., Görlitz, L., Hasenauer, J., Theis, F.J.: Comprehensive benchmarking of Markov chain monte Carlo methods for dynamical systems. BMC Syst. Biol. (2017). 10.1186/s12918-017-0433-1 PMC5482939 28646868

[CR8] Bhatnagar, N., Randall, D.: Simulated tempering and swapping on mean-field models. J. Stat. Phys. **164**(3), 495–530 (2016). 10.1007/s10955-016-1526-8

[CR9] Daniels, H.E.: Saddlepoint approximations in statistics. Ann. Math. Stat. **25**(4), 631–650 (1954). 10.1214/aoms/1177728652

[CR10] Diem, M.: Modern Vibrational Spectroscopy and Micro-Spectroscopy: Theory, Instrumentation and Biomedical Applications. Wiley, Chichester, West Sussex, UK (2015). 10.1002/9781118824924

[CR11] Duane, S., Kennedy, A.D., Pendleton, B.J., Roweth, D.: Hybrid monte carlo. Phys. Lett. B **195**(2), 216–222 (1987). 10.1016/0370-2693(87)91197-x

[CR12] Drton, M., Richardson, T.S.: Multimodality of the likelihood in the bivariate seemingly unrelated regressions model. Biometrika **91**(2), 383–392 (2004). 10.1093/biomet/91.2.383

[CR13] Durrett, R.: Probability: Theory and Examples. Cambridge University Press, Cambridge, UK (2010). 10.1017/cbo9780511779398

[CR14] Feroz, F., Hobson, M.P., Bridges, M.: Multinest: an efficient and robust bayesian inference tool for cosmology and particle physics. Mon. Not. R. Astron. Soc. **398**(4), 1601–1614 (2009). 10.1111/j.1365-2966.2009.14548.x

[CR15] Fiebig, D.G.: Seemingly unrelated regression. In: Baltagi, B.H. (ed.) A Companion to Theoretical Econometrics, pp. 101–121. John Wiley & Sons, USA (2003). 10.1002/9780470996249.ch6

[CR16] Fong, E., Lyddon, S., Holmes, C.: Scalable nonparametric sampling from multimodal posteriors with the posterior bootstrap. In: Chaudhuri, K., Salakhutdinov, R. (eds.) Proceedings of the 36th International Conference on Machine Learning. Proceedings of Machine Learning Research, vol. 97, pp. 1952–1962. PMLR, Long Beach, CA, USA (2019). https://proceedings.mlr.press/v97/fong19a.html

[CR17] Frühwirth-Schnatter, S.: Markov chain Monte Carlo estimation of classical and dynamic switching and mixture models. J. Am. Stat. Assoc. **96**(453), 194–209 (2001). 10.1198/016214501750333063

[CR18] Freeman, J.J., Wang, A., Kuebler, K.E., Jolliff, B.L., Haskin, L.A.: Characterization of natural feldspars by Raman spectroscopy for future planetary exploration. Can. Mineral. **46**(6), 1477–1500 (2008). 10.3749/canmin.46.6.1477

[CR19] Geyer, C.J.: Markov Chain Monte Carlo Maximum Likelihood. In: Computing Science and Statistics: Proceedings of the 23rd Symposium on the Interface, pp. 156–163. Interface Foundation of North America, Fairfax Station, VA (1991)

[CR20] Greene, W.H.: Econometric Analysis, 3rd edn. Prentice Hall, Upper Saddle River, NJ (1997)

[CR21] Grunfeld, Y.: The determinants of corporate investment, University of Chicago (1958). (PhD thesis)

[CR22] Geyer, C.J., Thompson, E.A.: Annealing markov chain monte carlo with applications to ancestral inference. J. Am. Stat. Assoc. **90**(431), 909–920 (1995). 10.1080/01621459.1995.10476590

[CR23] Hastings, W.K.: Monte carlo sampling methods using markov chains and their applications. Biometrika **57**(1), 97–109 (1970). 10.1093/biomet/57.1.97

[CR24] Henningsen, A., Hamann, J.D.: systemfit: A package for estimating systems of simultaneous equations inr. J. Stat. Softw. (2007). 10.18637/jss.v023.i04

[CR25] Härkönen, T., Hannula, E., Moores, M.T., Vartiainen, E.M., Roininen, L.: A log-gaussian cox process with sequential monte carlo for line narrowing in spectroscopy. Found. Data Sci. **5**(4), 503–519 (2023). 10.3934/fods.2023008

[CR26] Ibrahim, A.: New methods for mode jumping in Markov chain Monte Carlo algorithms, University of Bath (2009). (PhD thesis)

[CR27] Jasra, A., Holmes, C., Stephens, D.: Markov chain Monte Carlo methods and the label switching problem in Bayesian mixture modeling. Stat. Sci., 50–67 (2005). 10.1214/088342305000000016

[CR28] Kingma, D.P., Ba, J.: Adam: A method for stochastic optimization. In: Bengio, Y., LeCun, Y. (eds.) 3rd International Conference on Learning Representations, ICLR 2015, San Diego, CA, USA, May 7-9, 2015, Conference Track Proceedings (2015). http://arxiv.org/abs/1412.6980

[CR29] Kmenta, J.: Elements of Econometrics, 2nd edn. University of Michigan Press, Ann Arbor, MI (1997)

[CR30] Kleiber, C., Zeileis, A.: The grunfeld data at 50: The grunfeld data at 50. German Econ. Rev. **11**(4), 404–417 (2010). 10.1111/j.1468-0475.2010.00513.x

[CR31] Kou, S.C., Zhou, Q., Wong, W.H.: Equi-energy sampler with applications in statistical inference and statistical mechanics. Ann. Stat. (2006). 10.1214/009053606000000515

[CR32] Liu, J.S.: Metropolized independent sampling with comparisons to rejection sampling and importance sampling. Stat. Comput. **6**(2), 113–119 (1996). 10.1007/bf00162521

[CR33] Liu, J.S., Liang, F., Wong, W.H.: The multiple-try method and local optimization in metropolis sampling. J. Am. Stat. Assoc. **95**(449), 121–134 (2000). 10.1080/01621459.2000.10473908

[CR34] Moores, M., Gracie, K., Carson, J., Faulds, K., Graham, D., Girolami, M.: Bayesian modelling and quantification of Raman spectroscopy. In: Wood, D.R., Etheridge, A.M., de Gier, J., Joshi, N. (eds.) MATRIX Annals, vol. 9. Springer, Cham (2026)

[CR35] Miasojedow, B., Moulines, E., Vihola, M.: An adaptive parallel tempering algorithm. J. Comput. Graph. Stat. **22**(3), 649–664 (2013)

[CR36] Marinari, E., Parisi, G.: Simulated tempering: A new monte carlo scheme. Europhys. Lett. (EPL) **19**(6), 451–458 (1992). 10.1209/0295-5075/19/6/002

[CR37] Mangoubi, O., Pillai, N.S., Smith, A.: Does Hamiltonian Monte Carlo mix faster than a random walk on multimodal densities? (2018). https://arxiv.org/abs/1808.03230

[CR38] Metropolis, N., Rosenbluth, A.W., Rosenbluth, M.N., Teller, A.H., Teller, E.: Equation of state calculations by fast computing machines. J. Chem. Phys. **21**(6), 1087–1092 (1953) 10.1063/5.0309018 41342507

[CR39] Neal, R.M.: Sampling from multimodal distributions using tempered transitions. Stat. Comput. **6**(4), 353–366 (1996). 10.1007/bf00143556

[CR40] Neal, R.M.: Handbook of Markov Chain Monte Carlo, Chapman and Hall/CRC (2011). (Mcmc using hamiltonian dynamics)

[CR41] Nemeth, C., Lindsten, F., Filippone, M., Hensman, J.: Pseudo-extended markov chain monte carlo. In: Proceedings of the 33rd International Conference on Neural Information Processing Systems, pp. 4312–4322. Curran Associates Inc, Red Hook, NY, USA (2019)

[CR42] O’Brien, T., Moores, M.T., Warton, D., Falster, D.: Here Be Dragons: Bimodal posteriors arise from numerical integration error in longitudinal models (2026). https://arxiv.org/abs/2502.11510

[CR43] Papamarkou, T., Hinkle, J., Young, M.T., Womble, D.: Challenges in markov chain monte carlo for bayesian neural networks. Stat. Sci. (2022). 10.1214/21-sts840

[CR44] Pompe, E., Holmes, C., Łatuszyński, K.: A framework for adaptive mcmc targeting multimodal distributions. Ann. Stat. (2020). 10.1214/19-aos1916

[CR45] Queniat, H.: On the limits and extensions of the annealed leap-point sampler, Master’s thesis, Institut Polytechnique de Paris (2024)

[CR46] Robert, C.P., Casella, G.: Monte Carlo Statistical Methods. Springer, New York (2004). 10.1007/978-1-4757-4145-2

[CR47] Rossky, P.J., Doll, J.D., Friedman, H.L.: Brownian dynamics as smart monte carlo simulation. J. Chem. Phys. **69**(10), 4628–4633 (1978). 10.1063/1.436415

[CR48] Roberts, G.O., Gelman, A., Gilks, W.R.: Weak convergence and optimal scaling of random walk metropolis algorithms. Ann. Appl. Probab. (1997). 10.1214/aoap/1034625254

[CR49] Ritter, C.: Statistical analysis of spectra from electron spectroscopy for chemical analysis. The Statistician **43**(1), 111 (1994). 10.2307/2348937

[CR50] Roberts, G.O., Rosenthal, J.S.: Optimal scaling for various metropolis-hastings algorithms. Stat. Sci. (2001). 10.1214/ss/1015346320

[CR51] Roberts, G.O., Rosenthal, J.S.: Examples of adaptive mcmc. J. Comput. Graph. Stat. **18**(2), 349–367 (2009). 10.1198/jcgs.2009.06134

[CR52] Roberts, G.O., Rosenthal, J.S., Tawn, N.G.: Skew brownian motion and complexity of the alps algorithm. J. Appl. Probab. **59**(3), 777–796 (2022). 10.1017/jpr.2021.78

[CR53] Roberts, G.O., Tweedie, R.L.: Exponential convergence of langevin distributions and their discrete approximations. Bernoulli **2**(4), 341 (1996). 10.2307/3318418

[CR54] Syed, S., Bouchard-Côté, A., Deligiannidis, G., Doucet, A.: Non-reversible parallel tempering: A scalable highly parallel mcmc scheme. J. R. Stat. Soc. Ser. B Stat Methodol. **84**(2), 321–350 (2021). 10.1111/rssb.12464

[CR55] Srivastava, V.K., Giles, D.E.A.: Seemingly Unrelated Regression Equation Models: Estimation and Inference. Marcel Dekker, New York (1987). 10.1201/9781003065654

[CR56] Searle, S.R., Khuri, A.I.: Matrix Algebra Useful for Statistics, 2nd edn. Wiley Series in Probability and Statistics. John Wiley & Sons, Nashville, TN (2017)

[CR57] Surjanovic, N., Syed, S., Bouchard-Côté, A., Campbell, T.: Parallel tempering with a variational reference. In: Proceedings of the 36th International Conference on Neural Information Processing Systems, Curran Associates Inc, New Orleans, LA, USA (2022)

[CR58] Sundberg, R.: Flat and multimodal likelihoods and model lack of fit in curved exponential families. Scand. J. Stat. **37**(4), 632–643 (2010). 10.1111/j.1467-9469.2010.00703.x

[CR59] Tjelmeland, H., Eidsvik, J.: On the use of local optimizations within metropolis–hastings updates. J. R. Stat. Soc. Ser. B Stat Methodol. **66**(2), 411–427 (2004). 10.1046/j.1369-7412.2003.05329.x

[CR60] Tjelmeland, H., Hegstad, B.K.: Mode jumping proposals in mcmc. Scand. J. Stat. **28**(1), 205–223 (2001). 10.1111/1467-9469.00232

[CR61] Tawn, N.G., Roberts, G.O.: Optimal Temperature Spacing for Regionally Weight-preserving Tempering. (2018). https://arxiv.org/abs/1810.05845

[CR62] Tawn, N.G., Roberts, G.O.: Accelerating parallel tempering: Quantile tempering algorithm (quanta). Adv. Appl. Probab. **51**(03), 802–834 (2019). 10.1017/apr.2019.35

[CR63] Tawn, N.G., Roberts, G.O., Rosenthal, J.S.: Weight-preserving simulated tempering. Stat. Comput. **30**(1), 27–41 (2019). 10.1007/s11222-019-09863-3

[CR64] van Dyk, D.A., Connors, A., Kashyap, V.L., Siemiginowska, A.: Analysis of energy spectra with low photon counts via Bayesian posterior simulation. Astrophys. J. **548**(1), 224–243 (2001). 10.1086/318656

[CR65] Wang, J.-S., Swendsen, R.H.: Cluster monte carlo algorithms. Physica A **167**(3), 565–579 (1990). 10.1016/0378-4371(90)90275-w

[CR66] Woodard, D., Schmidler, S., Huber, M.: Sufficient conditions for torpid mixing of parallel and simulated tempering. Electron. J. Probab. (2009). 10.1214/ejp.v14-638

[CR67] Woodard, D.B., Schmidler, S.C., Huber, M.: Conditions for rapid mixing of parallel and simulated tempering on multimodal distributions. Ann. Appl. Probab. (2009). 10.1214/08-aap555

[CR68] Wang, Y., Zhou, X., Wang, H., Li, K., Yao, L., Wong, S.T.C.: Reversible jump mcmc approach for peak identification for stroke seldi mass spectrometry using mixture model. Bioinformatics **24**(13), 407–413 (2008). 10.1093/bioinformatics/btn143 PMC2718621 18586741

[CR69] Zellner, A.: An efficient method of estimating seemingly unrelated regressions and tests for aggregation bias. J. Am. Stat. Assoc. **57**(298), 348–368 (1962). 10.1080/01621459.1962.10480664

[CR70] Zhong, M., Girolami, M., Faulds, K., Graham, D.: Bayesian methods to detect dye-labelled dna oligonucleotides in multiplexed raman spectra. J. R. Stat. Soc.: Ser. C: Appl. Stat. **60**(2), 187–206 (2011). 10.1111/j.1467-9876.2010.00744.x

[CR71] Zhou, Q.: Multi-domain sampling with applications to structural inference of bayesian networks. J. Am. Stat. Assoc. **106**(496), 1317–1330 (2011). 10.1198/jasa.2011.ap10346

[CR72] Zhou, Q.: Random walk over basins of attraction to constructising energy landscapes. Phys. Rev. Lett. (2011). 10.1103/physrevlett.106.180602 21635078

