# Supplementary material for: Annealed leap-point sampler for multimodal target distributions
Source: Stat Comput. 2026 Oct 17;36(6):239. doi: 10.1007/s11222-026-10988-5 (PMC13676543; doi:10.1007/s11222-026-10988-5)
Supplement: Supplementary file 1 [file 11222_2026_10988_MOESM1_ESM.pdf]

Web-based supporting materials for

Annealed Leap-Point Sampler for Multimodal Target Distributions

by Nicholas G. Tawn   Matthew T. Moores   Hugo Queniat

Gareth O. Roberts

May 1, 2026

Contents

A Proofs **2**

A.1 Traditional set of assumptions 2

A.1.1 Auxiliary Results 4

A.1.2 Main result 10

A.2 Relaxed assumptions 17

A.2.1 Technical Lemma 17

A.2.2 Asymptotic Behaviour of the Logged Acceptance Ratio 24

A.2.3 Deriving the final result 33

B The HAT distributions **34**

B.1 Weight-Stabilised 34

B.2 Truncated HAT distributions 36

C Pseudo-code implementation of ALPS **39**

D Example 4: 4-Modal 20-Dimensional Skew-Normal **40**

E Seemingly-Unrelated Regression Model **45**

E.1 Further results 47

A Proofs

A.1 Traditional set of assumptions

For all proofs that follow, we shall, for notational convenience, denote $\beta_{\max}$ simply by β as that is the only temperature considered in this analysis.

The added complexity of the setup with the introduction of an augmented allocation component given in (9) allows for significant simplification in the theoretical analysis.

Suppose that $(k, x) \sim \pi_\beta(\cdot, \cdot)$. Suppose that the independence sampler move based on (15) proposes the point $(u, y) \sim q_\beta(\cdot, \cdot)$. Then the acceptance ratio after some basic cancellation of terms would be given by

$$\frac{\pi_\beta(u,y)q_\beta(k,x)}{\pi_\beta(k,x)q_\beta(u,y)} = \prod_{i=1}^{d} \frac{\left[f\left(\frac{y_i-\mu_{iu}}{\sigma_{iu}}\right)\right]^\beta \left[\phi_\beta\left(\frac{x_i-\mu_{ik}}{\sigma_{ik}}\right)\right]}{\left[f\left(\frac{x_i-\mu_{ik}}{\sigma_{ik}}\right)\right]^\beta \left[\phi_\beta\left(\frac{y_i-\mu_{iu}}{\sigma_{iu}}\right)\right]}. \tag{29}$$

Due to the data augmentation component, wlog for the evaluation of the acceptance

ratio we may assume that $\mu_{ij} = 0$ for all $i \in \{1, \ldots, d\}$ and $j \in \{1, \ldots, m\}$, i.e. the modes in all components are centred at $0 \in \mathbb{R}^d$.

Furthermore, by a similar argument, one can see from simple calculations that the acceptance ratio is unchanged by a rescaling of the regions conditional on the components provided the components of the independence sampler proposal undergo identical rescaling. As such we can assume wlog that $\sigma_{ij} = 1$ for all $i \in \{1, \ldots, d\}$ and $j \in \{1, \ldots, m\}$.

This dramatically simplifies the problem. In fact, wlog the problem reduces to studying the behaviour of an independence sampler move on just a d-dimensional target distribution on $\mathbb{R}^d$ (without the component allocation) given by

$$\pi_\beta(x) \propto \prod_{i=1}^{d} f^\beta(x_i) \tag{30}$$

with independence sampler proposals generated by

$$q_\beta(x) = \prod_{i=1}^{d} \phi_\beta(x_i). \tag{31}$$

To this end, suppose that the cold state component of the ALPS Markov chain is at a position x and that a Gaussian independence sampler proposal, y, is made as in the ALPS scheme; then the probability of acceptance for the proposal is given by

$$A_{IS}(x, y) = \min\left\{1, e^{B(d)}\right\} \tag{32}$$

where

$$e^{B(d)} = \prod_{i=1}^{d} \frac{f^\beta(y_i)\phi_\beta(x_i)}{\phi_\beta(y_i) f^\beta(x_i)} \tag{33}$$

where $x_i \sim f^\beta$, $y_i \sim \phi_\beta$ with ϕ_β denoting the pdf of a Gaussian of the form $N\left(0, (\beta|H|)^{-1}\right)$ which is the Gaussian approximation that the ALPS algorithm implements about the mode

in this case.

The proof of Theorem 5.1, deferred to this appendix, relies on an asymptotic analysis of 32 in the high-dimensional regime, as formalized in 16. Prior to the proof, we establish a number of essential preliminary results.

A.1.1 Auxiliary Results

For notational convenience herein denote

$$B_\epsilon(L) := \{x \in \mathbb{R} : |x - L| < \epsilon\}$$

and formally define the normalisation constant.

Definition A.1 (Normalisation at level β). For the un-normalised marginal component of the product target specified in Theorem 5.1, i.e. $f(\cdot)$, then define the normalisation constant

$$N(\beta) := \int [f(x)]^\beta dx \tag{34}$$

Definition A.2 (Asymptotic Gaussian standard deviation). With $f(\cdot)$ as in Theorem 5.1 with $H = h''(0)$, define

$$\sigma(\beta) = \frac{1}{\sqrt{\beta|H|}}. \tag{35}$$

The following few propositions work on integrals that use the un-normalised density. It will be clear when the normalisation constant comes in. In fact, it will be shown that $N(\beta) = \mathcal{O}(\beta^{1/2})$.

Proposition 1 (Exponential decay of tail integrals). With $f(\cdot)$ as in Theorem 5.1 and specifically for the polynomial tail condition given in (13) then for each $k \geq 0$ there exist constants

$C_1 > 0$ and $C_2 \geq 0$ such that

$$\limsup_{\beta\to\infty} \left| e^{C_1\beta} \int_{\mathbb{R}\setminus B_M(0)} z^k f^\beta(z) dz \right| = C_2 < \infty$$

Proof. With K, γ and M all specified in (13) (and wlog $K < M$) and assuming β sufficiently large

$$\begin{aligned} \left| \int_M^\infty z^k f^\beta(z) dz \right| &\leq \int_M^\infty |z|^k |z|^{-\gamma\beta} \frac{f^\beta(z)}{|z|^{-\gamma\beta}} dz \\ &\leq K^\beta \int_M^\infty |z|^{k-\gamma\beta} dz \\ &\leq \left(\frac{K}{M^\gamma} \right)^\beta \frac{M^{k+1}}{(\gamma\beta - (k+1))} \\ &= \frac{M^{k+1}}{(\gamma\beta - (k+1))} e^{-\beta |\log(K/M)|} \end{aligned}$$

and thus the conclusion of the proposition follows trivially. □

Herein, for notational convenience, we denote

$$I_k(a,b) := \int_a^b z^k f^\beta(z) dz$$

Proposition 2 (Intermediate integral exponential decay)*.* For $r \leq 1/3$ and $f(\cdot)$ as in Theorem 5.1, specifically the Dutchman's cap assumption in (14), then for each $k \geq 0$ there exists a constant $D_1 > 0$ such that

$$\limsup_{\beta\to\infty} \left| e^{D_1 \beta^{(1-2r)}} \left(I_k(-M, -M\beta^{-r}) + I_k(M\beta^{-r}, M) \right) \right| = 0 < \infty \qquad (36)$$

Proof. Take δ_2 as specified in the Dutchman's cap assumption in (14) and recall we have

$$f(z) \leq \begin{cases} \exp\left(-z^2 \frac{|H|}{4}\right), & \text{if } |z| < \delta_2. \\ \exp\left(-\delta_2^2 \frac{|H|}{4}\right), & \text{if } \delta_2 \leq |z| \leq M. \end{cases} \qquad (37)$$

Assume that β is sufficiently large so that $M\beta^{-r} < \delta_2$. By splitting the integral

$$I_k(M\beta^{-r}, M) = I_k(M\beta^{-r}, \delta_2) + I_k(\delta_2, M).$$

Focusing on the first term and using (37) and noting that $1 - 2r > 0$

$$\begin{aligned} |I_k(M\beta^{-r}, \delta_2)| &= \int_{M\beta^{-r}}^{\delta_2} z^k f^\beta(z) dz \leq \int_{M\beta^{-r}}^{\delta_2} z^k \exp\left(-z^2 \beta \frac{|H|}{4}\right) dz \\ &\leq \int_{M\beta^{-r}}^{\delta_2} \delta_2^k \exp\left(-M^2 \beta^{(1-2r)} \frac{|H|}{4}\right) dz \\ &= \delta_2^k \left[\delta_2 - M\beta^{-r}\right] \exp\left(-M^2 \beta^{(1-2r)} \frac{|H|}{4}\right). \end{aligned} \tag{38}$$

Then for the second term again using (37) then

$$\begin{aligned} |I_k(\delta_2, M)| &= \int_{\delta_2}^{M} z^k f^\beta(z) dz \leq \int_{\delta_2}^{M} M^k \exp\left(-\delta_2^2 \beta \frac{|H|}{4}\right) dz \\ &\leq (M - \delta_2) M^k \exp\left(-\delta_2^2 \beta \frac{|H|}{4}\right). \end{aligned} \tag{39}$$

Combining (38), (39) and an identical procedural application to the lower integrand, $I_k(-M, -M\beta^{-r})$, then the proposition is proved. □

Lemma A.3 (Limiting integrals)**.** *For $f(\cdot)$ in Theorem 5.1*

$$\lim_{\beta\to\infty} \left\{ \beta^{g(k)} \sqrt{\frac{\beta|H|}{2\pi}} \int_{\mathbb{R}} z^k f^\beta(z) dz \right\} = \begin{cases} \frac{\mathbb{E}(S^{k+3}) h'''(0)}{6|H|^{(k+3)/2}}, & \text{if } k \text{ is odd.} \\ \frac{\mathbb{E}(S^k)}{|H|^{k/2}}, & \text{if } k \text{ is even} \end{cases} \tag{40}$$

where S is distributed according to a standard normal distribution, i.e. $N(0,1)$, and

$$g(k) = \begin{cases} \frac{k+1}{2}, & \text{if } k \text{ is odd} \\ \frac{k}{2}, & \text{if } k \text{ is even.} \end{cases}$$

Proof. By Propositions 1 and 2 then one can derive that for $r = 1/3$

$$I_k(-\infty, \infty) = I_k(-M\beta^{-r}, M\beta^{-r}) + R(\beta) \tag{41}$$

where $R(\beta) = \mathcal{O}\left(\exp\left(-D\beta^{(1-2r)}\right)\right)$ for some constant $D > 0$ i.e. exponentially decaying in β. Consequently, we focus on $I_k(-M\beta^{-r}, M\beta^{-r})$.

By Taylor expansion with Taylor remainder $|\xi_z| < |z|$

$$\begin{aligned} f^\beta(z) &= \exp\left(\beta\left[z^2\frac{H}{2} + z^3\frac{h'''(0)}{6} + z^4\frac{h''''(\xi_z)}{24}\right]\right) \\ &= \sqrt{\frac{2\pi}{\beta|H|}}\phi_\beta(z)\exp\left(\beta z^3\frac{h'''(0)}{6}\right)\exp\left(\beta z^4\frac{h''''(\xi_z)}{24}\right) \end{aligned} \tag{42}$$

where $\phi_\beta(\cdot)$ denotes the pdf of a $N(0, \sigma(\beta)^2)$.

By further Taylor expansion

$$\exp\left(\beta z^3\frac{h'''(0)}{6}\right) = 1 + \beta z^3\frac{h'''(0)}{6} + \beta^2 z^6\frac{h'''(0)^2}{72}e^{\xi^1_{(\beta,z)}} \tag{43}$$

$$\exp\left(\beta z^4\frac{h''''(\xi_z)}{24}\right) = 1 + \beta z^4\frac{h''''(\xi_z)}{24}e^{\xi^2_{(\beta,z)}} \tag{44}$$

where $\xi^1_{(\beta,z)}$ and $\xi^2_{(\beta,z)}$ are Taylor remainders such that

$$|\xi^1_{(\beta,z)}| \leq \left|\beta z^3\frac{h'''(0)}{6}\right| \tag{45}$$

$$|\xi^2_{(\beta,z)}| \leq \left|\beta z^4\frac{h''''(\xi_z)}{24}\right|. \tag{46}$$

Combining (42), (43) and (44)

$$I_k(-M\beta^{-r}, M\beta^{-r}) = \sqrt{\frac{2\pi}{-\beta H}}\sum_{m=1}^{6} T_m(k) \tag{47}$$

where

$$\begin{aligned}
T_1(k) &= \int_{-M\beta^{-r}}^{M\beta^{-r}} z^k \phi_\beta(z) dz \\
T_2(k) &= \int_{-M\beta^{-r}}^{M\beta^{-r}} \beta z^{k+3} \frac{h'''(0)}{6} \phi_\beta(z) dz \\
T_3(k) &= \int_{-M\beta^{-r}}^{M\beta^{-r}} \beta^2 z^{k+6} \left(\frac{h'''(0)^2}{72}\right) e^{\xi^1_{(\beta,z)}} \phi_\beta(z) dz \\
T_4(k) &= \int_{-M\beta^{-r}}^{M\beta^{-r}} \beta z^{k+4} \left(\frac{h''''(\xi_z)}{24}\right) e^{\xi^2_{(\beta,z)}} \phi_\beta(z) dz \\
T_5(k) &= \int_{-M\beta^{-r}}^{M\beta^{-r}} \beta^2 z^{k+7} \left(\frac{h'''(0)}{6}\right) \left(\frac{h''''(\xi_z)}{24}\right) e^{\xi^2_{(\beta,z)}} \phi_\beta(z) dz \\
T_6(k) &= \int_{-M\beta^{-r}}^{M\beta^{-r}} \beta^3 z^{k+10} \left(\frac{h'''(0)^2}{72}\right) \left(\frac{h''''(\xi_z)}{24}\right) e^{\xi^1_{(\beta,z)}} e^{\xi^2_{(\beta,z)}} \phi_\beta(z) dz.
\end{aligned}$$

Consider the case that $k = 3$ and all others follow by identical methodology. For $k = 3$ it is clear that $T_1(3) = 0$ so consider $T_3(3)$ with (45) and using the fact that $e^x \leq e^{|x|}$ and $r = 1/3$

$$\begin{aligned}
|T_3(3)| &\leq \int_{-M\beta^{-r}}^{M\beta^{-r}} \beta^2 |z|^9 \left(\frac{h'''(0)^2}{72}\right) \exp\left[\beta |z|^3 \left|\frac{h'''(0)}{6}\right|\right] \phi_\beta(z) dz \\
&\leq \beta^2 \left(\frac{h'''(0)^2}{72}\right) \exp\left[M^3 \left|\frac{h'''(0)}{6}\right|\right] \int_{-M\beta^{-r}}^{M\beta^{-r}} |z|^9 \phi_\beta(z) dz \\
&\leq \beta^2 \left(\frac{h'''(0)^2}{72}\right) \exp\left[M^3 \left|\frac{h'''(0)}{6}\right|\right] \int_{\mathbb{R}} |z|^9 \phi_\beta(z) dz \\
&= C_3^3 \beta^2 \int_{\mathbb{R}} |z|^9 \phi_\beta(z) dz \qquad (48)
\end{aligned}$$

where $C_3^3 \in \mathbb{R}_+$ is a constant.

Similarly, there exists constants C_4^3, C_5^3, C_6^3 such that

$$|T_4(3)| \leq C_4^3\beta \int_{\mathbb{R}} |z|^7 \phi_\beta(z) dz \tag{49}$$

$$|T_5(3)| \leq C_5^3\beta^2 \int_{\mathbb{R}} |z|^{10} \phi_\beta(z) dz \tag{50}$$

$$|T_6(3)| \leq C_6^3\beta^3 \int_{\mathbb{R}} |z|^{13} \phi_\beta(z) dz. \tag{51}$$

Routine calculations using integration by parts reveals that

$$\int_{\mathbb{R}} |z|^m \phi_\beta(z) dz \leq \begin{cases} \frac{96}{\sqrt{2\pi}(\beta|H|)^{7/2}}, & \text{if } m = 7 \\ \frac{1536}{\sqrt{2\pi}(\beta|H|)^{9/2}}, & \text{if } m = 9 \\ \frac{945}{(\beta|H|)^5}, & \text{if } m = 10 \\ \frac{92160}{\sqrt{2\pi}(\beta|H|)^{13/2}}, & \text{if } m = 13 \end{cases} \tag{52}$$

Combining (48), (49), (50), (51) and (52) shows that for $m \in \{3, 4, 5, 6\}$

$$|T_m(3)| \leq \mathcal{O}\left(\frac{1}{\beta^{5/2}}\right). \tag{53}$$

Hence the crucial term is $T_2(3)$,

$$\begin{aligned} T_2(3) &= \int_{-M\beta^{-r}}^{M\beta^{-r}} \beta z^6 \frac{h'''(0)}{6} \phi_\beta(z) dz \\ &= \frac{h'''(0)}{6} \beta \int_{\mathbb{R}} z^6 \phi_\beta(z) dz + R_2(3) \end{aligned}$$

where using identical methodology to Propositions 1 and 2 one can show that for a constant $F > 0$ then $R_2(3) = \mathcal{O}\left(\beta^{5/2} e^{-F\beta^{2/3}}\right)$ and so trivially $R_2(3) = \mathcal{O}\left(\beta^{-5/2}\right)$. Hence,

$$T_2(3) = \frac{5h'''(0)}{2\beta^2|H|^3} + \mathcal{O}\left(\beta^{-5/2}\right). \tag{54}$$

Thus combining the results of (41), (47), (53) and (54) then

$$\lim_{\beta\to\infty} \left\{\beta^2 \sqrt{\frac{\beta|H|}{2\pi}} \int_{\mathbb{R}} z^k f^\beta(z) dz\right\} = \frac{5h'''(0)}{2|H|^3}. \tag{55}$$

This proves the lemma for k=3 and identical methodology all other values of $k \in \mathbb{N}_0$ follow. □

For the purposes of proving the main result the following corollary concatenates the key results from Lemma A.3.

Corollary 1 (Key moment results). From Lemma A.3 and the details of the proof it can be concluded that:

$$N(\beta) = \sqrt{\frac{2\pi}{-\beta H}} + \mathcal{O}\left(\beta^{-1}\right) \tag{56}$$

$$\mu_\beta := \int_{\mathbb{R}} z^3 \frac{f^\beta(z)}{N(\beta)} dz = \frac{5h'''(0)}{2\beta^2 |H|^3} + \mathcal{O}\left(\beta^{-5/2}\right) \tag{57}$$

$$\sigma_\beta^2 := \int_{\mathbb{R}} z^6 \frac{f^\beta(z)}{N(\beta)} dz = \frac{15}{\beta^3 |H|^3} + \mathcal{O}\left(\beta^{-7/2}\right) \tag{58}$$

Proof. All methodology and details from Lemma A.3 above. □

A.1.2 Main result

Now with the results established in Corollary 1 we are in a position to establish the result of Theorem 5.1.

Proof of Theorem 5.1. Recall the expected acceptance probability of a Mode Leap Point Independence sampler proposal at the coldest temperature level in this d-dimensional setting as

$$a(d) = \mathbb{E}(A_{IS}(x, y)) = \mathbb{E}(\min\left\{1, e^{B(d)}\right\}), \tag{59}$$

where the independence sampler acceptance probability, A_{IS}, is defined in (32) and the expectation is taken with respect to $x \sim \pi_\beta$ as defined in (30) and $y \sim q_\beta$ as defined in

(31).

As stated in (33), the logged acceptance ratio B is given by

$$B(d) = \sum_{i=1}^{d} \left[\beta h(y_i) - \log \phi_\beta(y_i)\right] - \left[\beta h(x_i) - \log \phi_\beta(x_i)\right]. \tag{60}$$

By Taylor expansion about the point 0 (which is the mode of $f(\cdot)$ and so $h'(0) = 0$) there exists ξ_x where $0 < |\xi_x| < |x|$ such that

$$h(x) = h(0) + \frac{x^2}{2}H + \frac{x^3}{6}h'''(0) + \frac{x^4}{24}h''''(0) + \frac{x^5}{120}h'''''(\xi_x). \tag{61}$$

Thus (60) becomes

$$\begin{aligned} B(d) &= \sum_{i=1}^{d} \Bigg[\frac{\beta h'''(0)}{6}(y_i^3 - x_i^3) + \frac{\beta h''''(0)}{24}\left(y_i^4 - x_i^4\right) \\ &\qquad + \frac{\beta}{120}\left(y_i^5 h'''''(\xi_{y_i}) - x_i^5 h'''''(\xi_{x_i})\right) \Bigg]. \end{aligned}$$

Lemma A.4. *Under the setting of Theorem 5.1, then if $\beta = \ell d$ as in (17) then the term*

$$W(d) := \sum_{i=1}^{d} \frac{\beta h'''(0)}{6}(y_i^3 - x_i^3) \tag{62}$$

is such that as $d \to \infty$

$$W(d) \Rightarrow N\left(-\frac{5h'''(0)^2}{12\ell|H|^3}, \frac{5h'''(0)^2}{6\ell|H|^3}\right) \tag{63}$$

where the convergence is weak convergence.

Proof. Since the distribution of the x_i and y_i is dependent on d then we are in the situation of triangular arrays. As such we aim to use the Lindeberg-Feller theorem, see e.g. Durrett (2010). Define for each $d \in \mathbb{N}$ and for $i \in \{1, \ldots, d\}$

$$X_{(d,i)} := \left(\frac{\beta h'''(0)}{6}\right)\left(x_i^3 - \mu_\beta\right).$$

Lindeberg-Feller Conditions: To apply the result of the Lindeberg-Feller theorem from Durrett (2010) to the $X_{(d,i)}$'s, it is sufficient to establish the following two conditions as $d \to \infty$

i) There exists $\sigma^2 \in \mathbb{R}_+$ such that

$$\sum_{i=1}^{d} \mathbb{E}(X_{(d,i)}^2) \to \sigma^2 > 0 \tag{64}$$

ii) For all $\epsilon > 0$ and with $A_\epsilon^d := \{|X_{(d,i)}| > \epsilon\}$

$$\lim_{d\to\infty} \sum_{i=1}^{d} \mathbb{E}(|X_{(d,i)}|^2 \mathbb{1}_{A_\epsilon}) = 0 \tag{65}$$

It is simple to show that L-F condition (i) in (64) holds, since by (57) and (58)

$$\mathbb{E}\left(X_{(d,i)}^2\right) = \frac{5h'''(0)^2}{12\ell d|H|^3} + \mathcal{O}\left(d^{-3/2}\right)$$

and so it is immediate that

$$\sum_{i=1}^{d} \mathbb{E}(X_{(d,i)}^2) \to \frac{5h'''(0)^2}{12\ell|H|^3} > 0 \tag{66}$$

To show that L-F condition (ii) in (65) holds is more involved. To this end let $\epsilon > 0$

$$\begin{aligned} & \mathbb{E}(|X_{(d,i)}|^2 \mathbb{1}_{A_\epsilon^d}) \\ = \; & \left(\frac{\beta h'''(0)}{6}\right)^2 \left[\mathbb{E}\left(x_i^6 \mathbb{1}_{A_\epsilon^d}\right) - 2\mu_\beta \mathbb{E}\left(x_i^3 \mathbb{1}_{A_\epsilon^d}\right) + \mu_\beta^2 \mathbb{P}(A_\epsilon^d)\right] & (67) \\ \leq \; & \left|\frac{\beta h'''(0)}{6}\right|^2 \left[\mathbb{E}\left(x_i^6 \mathbb{1}_{A_\epsilon^d}\right) + 2|\mu_\beta| \mathbb{E}\left(|x_i|^3 \mathbb{1}_{A_\epsilon^d}\right) + \mu_\beta^2 \mathbb{P}(A_\epsilon^d)\right] & (68) \end{aligned}$$

Using identical methodology to that used in proving Lemma A.3 with only a trivial extension one can show that

$$\mathbb{E}\left(|x_i|^3 \mathbb{1}_{A_\epsilon^d}\right) \leq \mathcal{O}\left(d^{-3/2}\right)$$

and so with $\mu_\beta = \mathcal{O}\left(d^{-2}\right)$ given in (57) then

$$\begin{aligned} |\mu_\beta|\mathbb{E}\left(|x_i|^3 \mathbb{1}_{A_\epsilon^d}\right) &\leq \mathcal{O}\left(d^{-7/2}\right) \\ |\mu_\beta^2 \mathbb{P}(A_\epsilon^d)| &\leq |\mu_\beta^2| = \mathcal{O}\left(d^{-4}\right) \end{aligned}$$

and thus we can conclude that

$$\lim_{d\to\infty} \sum_{i=1}^{d} \left|\frac{\beta h'''(0)}{6}\right|^2 \left[2\mu_\beta \mathbb{E}\left(x_i^3 \mathbb{1}_{A_\epsilon^d}\right) + \mu_\beta^2 \mathbb{P}(A_\epsilon^d)\right] = 0. \tag{69}$$

Consequently we must analyse the behaviour of $\mathbb{E}\left(x^6 \mathbb{1}_{A_\epsilon^d}\right)$. We begin by noting that A_ϵ^d can be equivalently written as

$$\begin{aligned} A_\epsilon^d &= \left\{x > \left(\mu_\beta + \frac{6\epsilon}{\beta|h'''(0)|}\right)^{1/3}\right\} \cup \left\{x < \left(\mu_\beta - \frac{6\epsilon}{\beta|h'''(0)|}\right)^{1/3}\right\} \\ &:= F_{+\epsilon}^d \cup F_{-\epsilon}^d. \end{aligned} \tag{70}$$

With this notation,

$$\mathbb{E}\left(x^6 \mathbb{1}_{A_\epsilon^d}\right) = \mathbb{E}\left(x^6 \mathbb{1}_{F_{+\epsilon}^d}\right) + \mathbb{E}\left(x^6 \mathbb{1}_{F_{-\epsilon}^d}\right). \tag{71}$$

By (57) we have that $\mu_\beta = \mathcal{O}(d^{-2})$ and so from the equivalent formulation of A_ϵ^d in (70) and for d sufficiently large there exists a constant $J > 0$ such that

$$\begin{aligned} F_{+\epsilon}^d &= \left\{x > \left(\mu_\beta + \frac{6\epsilon}{\beta|h'''(0)|}\right)^{1/3}\right\} \\ &\subseteq \left\{x > \frac{J}{d^{1/3}}\right\}. \end{aligned} \tag{72}$$

Focusing on the first term on the RHS of (71) and with M from (13)

$$\mathbb{E}\left(x^6 \mathbb{1}_{F_{+\epsilon}^d}\right) \leq \frac{1}{N(\beta)} \left[I_6(Jd^{-1/3}, M) + I_6(M, \infty)\right]. \tag{73}$$

By Propositions 1 and 2 then there exists a constant $W_1 > 0$ such that the RHS of (73) is $\mathcal{O}\left(e^{-W_1 d^{1/3}}\right)$. By identical methodology one can show that there exists $W_2 > 0$ such that

$$\mathbb{E}\left(x^6 \mathbb{1}_{F_{-\epsilon}^d}\right) \leq \mathcal{O}\left(e^{-W_2 d^{1/3}}\right)$$

and so with $W_3 := \min\{W_1, W_2\}$ then

$$\mathbb{E}\left(x^6 \mathbb{1}_{A_\epsilon^d}\right) = \mathcal{O}\left(e^{-W_3 d^{1/3}}\right). \tag{74}$$

Consequently by (74) then

$$\lim_{d\to\infty} \sum_{i=1}^{d} \left|\frac{\beta h'''(0)}{6}\right|^2 \mathbb{E}\left(x_i^6 \mathbb{1}_{A_\epsilon^d}\right) = 0. \tag{75}$$

So combining (69) and (75) proves that the second Lindeberg-Feller condition in (65) holds.

With (64) and (65) holding for the triangular array of $X_{(d,i)}$'s then the Lindeberg-Feller theorem implies that that as $d \to \infty$

$$-\sum_{i=1}^{d} X_{(d,i)} \Rightarrow N\left(0, \frac{15h'''(0)^2}{36\ell|H|^3}\right).$$

However, by using the result of (57) that gives an explicit value to the μ_β and the definition of the $X_{(d,i)}$'s then

$$-\sum_{i=1}^{d} \frac{\beta h'''(0)}{6} x_i^3 \Rightarrow N\left(-\frac{15h'''(0)^2}{36\ell|H|^3}, \frac{5h'''(0)^2}{12\ell|H|^3}\right). \tag{76}$$

For a given d, the $y_i \sim N(0, (\beta|H|)^{-1})$, thus they too fall into a triangular arrays setup with

$$\mathbb{E}(y_i^3) = 0 \tag{77}$$

$$\mathrm{Var}(y_i^3) = \mathbb{E}(y_i^6) = \frac{15}{\beta^3|H|^3} \tag{78}$$

and so setting

$$Y_{(d,i)} = \left(\frac{\beta h'''(0)}{6}\right) y_i^3$$

one can follow a simplified version of the above Lindeberg-Feller procedure for the $X_{(d,i)}$'s to establish that as $d \to \infty$

$$\sum_{i=1}^{d} \frac{\beta h'''(0)}{6} y_i^3 \Rightarrow N\left(0, \frac{5h'''(0)^2}{12\ell(-h''(0))^3}\right) \tag{79}$$

Using the fact that the x_i's and y_i's are independent along with the weak convergences established in (76) and (79) the result of Lemma A.4 is established. □

Lemma A.5. *Under the setting of Theorem 5.1, then if $\beta = \ell d$ as in (17) then as $d \to \infty$*

$$T_1(d) := \sum_{i=1}^{d} \frac{\beta h''''(0)}{24} \left(y_i^4 - x_i^4 \right) \to 0 \quad in \;\; \mathbb{P}. \tag{80}$$

Proof. Fix $\epsilon > 0$. Using identical methodology to that used in Lemma A.3 then it can be shown that

$$\mathbb{E}(x_i^4) = \frac{3}{(\beta|H|)^2} + \mathcal{O}\left(\beta^{-3}\right) \tag{81}$$

$$\mathbb{E}(x_i^8) = \frac{105}{(\beta|H|)^4} + \mathcal{O}\left(\beta^{-5}\right) \tag{82}$$

and from standard calculations using the Gaussianity of the y_i's

$$\mathbb{E}(y_i^4) = \frac{3}{(\beta|H|)^2} \tag{83}$$

$$\mathbb{E}(y_i^8) = \frac{105}{(\beta|H|)^4}. \tag{84}$$

Defining $\mu_{T_1(d)} := \mathbb{E}(T_1(d))$ then by (81) and (83) as $d \to \infty$

$$\mu_{T_1(d)} \to 0. \tag{85}$$

Thus there exists $U > 0$ such that for all $d > U$ then $|\mu_{T_1(d)}| < \frac{\epsilon}{2}$. Furthermore, by (81), (83), (82) and (84) then

$$\mathrm{Var}(T_1(d)) \to 0 \;\; \text{as} \;\; d \to 0. \tag{86}$$

For $d > U$ and using Chebyshev's inequality and utilising (86)

$$\mathbb{P}\left(|T_1(d)| > \epsilon\right) \leq \mathbb{P}\left(|T_1(d) - \mu_{T_1(d)}| \geq \frac{\epsilon}{2}\right) \leq \frac{4\mathrm{Var}(T_1(d))}{\epsilon^2} \to 0 \;\; \text{as} \;\; d \to 0$$

and so the result of Lemma A.5 holds. □

Lemma A.6. *Under the setting of Theorem 5.1, then if $\beta = \ell d$ as in (17) then*

$$T_2(d) := \sum_{i=1}^{d} \frac{\beta}{120} \left(y_i^5 h'''''(\xi_{y_i}) - x_i^5 h'''''(\xi_{x_i}) \right) \tag{87}$$

is such that $T_2(d) \to 0$ in $\mathbb{P}$ as $d \to \infty$.

Proof. Using the assumption given in (12)

$$|T_2(d)| \leq \sum_{i=1}^{d} L \frac{\beta}{120} \left(|y_i|^5 + |x_i|^5 \right) =: T_2^r(d) > 0 \tag{88}$$

and so if one can show that $T_2^r(d) \to 0$ in $\mathbb{P}$ then the required result in (87) follows. Note that

$$\mathbb{E}(|y_i|^5) = \frac{16}{\sqrt{2\pi} \left(\beta |H| \right)^{5/2}} \tag{89}$$

and using identical methodology to the proof of Lemma A.3 then it can be shown that for some constant $E \in \mathbb{R}$

$$\mathbb{E}(|x_i|^5) = \frac{E}{d^{5/2}} + \mathcal{O}\left(d^{-3} \right) \tag{90}$$

Let $\epsilon > 0$, then by Markov inequality and then using (89) and (90)

$$\begin{aligned} \mathbb{P}(T_2^r(d) > \epsilon) &\leq \frac{L\ell d}{24\epsilon} \sum_{i=1}^{d} \left[\mathbb{E}(|y_i|^5) + \mathbb{E}(|x_i|^5) \right] \\ &= \frac{L\ell d^2}{24\epsilon} \left[\mathbb{E}(|y_1|^5) + \mathbb{E}(|x_1|^5) \right] \\ &\to 0 \quad \text{as} \quad d \to \infty. \end{aligned}$$

Thus the result of Lemma A.6 holds. □

Taking the results of Lemmata A.4, A.5 and A.6 and trivial application of Slutsky's Theorem, then we have that as $d \to \infty$ then

$$B(d) = W(d) + T_1(d) + T_2(d) \Rightarrow B \sim N\left(-\frac{5h'''(0)^2}{12\ell(-h''(0))^3}, \frac{5h'''(0)^2}{6\ell(-h''(0))^3} \right) \tag{91}$$

where the convergence is weak convergence.

We are interested in the behaviour of $\mathbb{E}\left[\min\left\{1, e^{B(d)}\right\}\right]$ in the limit as $d \to \infty$. Since the function, $\min\left\{1, e^{B(d)}\right\}$, is a bounded continuous function of $B(d)$ then by weak convergence of $B(d)$ established above in (91), then

$$\lim_{d\to\infty} \mathbb{E}\left[\min\left\{1, e^{B(d)}\right\}\right] = \mathbb{E}\left[\min\left\{1, e^{B}\right\}\right].$$

Proposition 3. Suppose that $G \sim N(-\frac{\sigma^2}{2}, \sigma^2)$,

$$\mathbb{E}\left(1 \wedge e^{G}\right) = 2\Phi\left(-\frac{\sigma}{2}\right).$$

Proof. A routine calculation (see e.g., Roberts et al. (1997)). □

Applying Proposition 3 to the asymptotic form of B in (91) immediately gives the required result and completes the proof of Theorem 5.1. □

A.2 Relaxed assumptions

This section extends the previous proof to a slightly more general setting, obtained by relaxing the assumptions; see Theorem 5.2.

A.2.1 Technical Lemma

First, we define the step functions that will be used when dealing with left and right derivatives.

Definition A.7. (Step functions). We denote by $\mathcal{S}_0$ the class of step functions, such that for $g \in \mathcal{S}_0$,

$$\forall x \neq 0,\ g(x) = g(0_-)\mathbb{1}_{(-\infty,0)} + g(0_+)\mathbb{1}_{(0,+\infty)} \tag{92}$$

Then, the following very useful technical lemma allows us to compute quantities of interest with respect to the density considered in the relaxed scenario:

Lemma A.8 (Limiting integrals)**.** *For $f(\cdot)$ as in Theorem 5.2 and $g \in \mathcal{S}_0$,*

$$\int_{\mathbb{R}} g(z) z^k f(z)^\beta \mathrm{d}z = \sqrt{\frac{2\pi}{\beta|H|}} \Bigg[\frac{g(0_+) + (-1)^k g(0_-)}{2\,(\beta|H|)^{k/2}} \mathbb{E}\left[|S|^k\right] + \frac{g(0_+)h'''(0_+) + (-1)^{k+1} g(0_-)h'''(0_-)}{12\beta^{(k+1)/2}|H|^{(k+3)/2}} \mathbb{E}\left[|S|^{k+3}\right] + O\big(\beta^{-(k+2)/2}\big) \Bigg]. \tag{93}$$

where S is distributed according to a standard normal distribution, i.e. $N(0,1)$.

Proof. For notational convenience, we denote :

$$h_* := \max\left(|h'''(0_-)|, |h'''(0_+)|\right)$$

Proposition 4. (Exponential decay of tail integrals). With $f(\cdot)$ as defined earlier and specifically for the polynomial tail condition given in (13) and for any $g \in \mathcal{S}_0$, then for each $k \geq 0$, there exists constants $C_1 > 0$ and $C_2 \geq 0$ such that

$$\limsup_{\beta \to \infty} \left| e^{C_1 \beta} \int_{\mathbb{R} \setminus B_M(0)} g(z) z^k f^\beta(z) \mathrm{d}z \right| = C_2 < \infty \tag{94}$$

Proof. With K, γ and M all specified in (13) (and wlog $K < M^\gamma$) and assuming β suffi-

ciently large,

$$\begin{aligned}\left|\int_M^\infty g(z)z^k f^\beta(z)\mathrm{d}z\right| &\leq \int_M^\infty |g(z)||z|^k|z|^{-\gamma\beta}\frac{f^\beta(z)}{|z|^{-\gamma\beta}}\mathrm{d}z \\ &\leq K^\beta|g(0_+)|\int_M^\infty |z|^{k-\gamma\beta}\mathrm{d}z \\ &\leq |g(0_+)|\left(\frac{K}{M^\gamma}\right)^\beta \frac{M^{k+1}}{(\gamma\beta-(k+1))} \\ &= \frac{M^{k+1}|g(0_+)|}{(\gamma\beta-(k+1))}e^{-\beta|\log(K/M^\gamma)|}\end{aligned}$$

and thus the conclusion follows trivially. □

Moving further, once again for notational purposes, we denote for any $g \in \mathcal{S}_0$ - the class of step functions, see definition A.7 :

$$I_k^g(a,b) := \int_a^b g(z)z^k f^\beta(z)\mathrm{d}z$$

Proposition 5. (Intermediate integral exponential decay). For $r \leq 1/3$ and $f(\cdot)$ presented earlier, specifically the Dutchman's cap assumption in (14) and any $g \in \mathcal{S}_0$, then for each $k \geq 0$,there exists a constant $D_1 > 0$ such that

$$\limsup_{\beta\to\infty}\left|e^{D_1\beta^{(1-2r)}}\left(I_k^g\left(-M,-M\beta^{-r}\right)+I_k^g\left(M\beta^{-r},M\right)\right)\right| = 0 < \infty \tag{95}$$

Proof. Take δ_2 as specified in the Dutchman's cap assumption in (14).

Assume that β is sufficiently large so that $M\beta^{-r} < \delta_2$. By splitting the integral

$$I_k^p\left(M\beta^{-r},M\right) = I_k^g\left(M\beta^{-r},\delta_2\right) + I_k^g\left(\delta_2,M\right)$$

Focusing on the first term and using (14) and noting that $1-2r>0$

$$\begin{aligned}\left|I_k\left(M\beta^{-r},\delta_2\right)\right| &\leq \int_{M\beta^{-r}}^{\delta_2}|g(z)|z^k f^\beta(z)\mathrm{d}z \leq |g(0_+)|\int_{M\beta^{-r}}^{\delta_2} z^k \exp\left(-z^2\beta\frac{|H|}{4}\right)\mathrm{d}z \\ &\leq |g(0_+)|\int_{M\beta^{-r}}^{\delta_2}\delta_2^k\exp\left(-M^2\beta^{(1-2r)}\frac{|H|}{4}\right)\mathrm{d}z \\ &= |g(0_+)|\delta_2^k\left[\delta_2 - M\beta^{-r}\right]\exp\left(-M^2\beta^{(1-2r)}\frac{|H|}{4}\right)\end{aligned} \tag{96}$$

Then for the second term again using (14), we have

$$\begin{aligned}\left|I_k\left(\delta_2, M\right)\right| &\leq \int_{\delta_2}^{M}|g(z)|z^k f^\beta(z)\mathrm{d}z \leq |g(0_+)|\frac{\sqrt{\beta}}{J}\int_{\delta_2}^{M} M^k\exp\left(-\delta_2^2\beta\frac{|H|}{4}\right)\mathrm{d}z \\ &\leq |g(0_+)|\left(M-\delta_2\right)M^k\exp\left(-\delta_2^2\beta\frac{|H|}{4}\right)\end{aligned} \tag{97}$$

Combining (96), (97) and an identical procedural application to the lower integrand, $I_k^p\left(-M, -M\beta^{-r}\right)$, then the proposition is proved. □

Coming back to Lemma A.8, by propositions 4 and 5 then one can derive that for $r = 1/3$

$$I_k^g(-\infty,\infty) = I_k^g\left(-M\beta^{-r}, M\beta^{-r}\right) + R(\beta) \tag{98}$$

where $R(\beta) = \mathcal{O}\left(\exp\left(-D\beta^{(1-2r)}\right)\right)$ for some constant $D > 0$, i.e. exponentially decaying in β. Consequently, we focus on $I_k^p\left(-M\beta^{-r}, M\beta^{-r}\right)$.

By Taylor expansion with Taylor remainder $|\xi_z| < |z|$

$$
\begin{aligned}
f^\beta(z) &= \exp\left(\beta\left[z^2\frac{h''\big(0_{\mathrm{sgn}(z)}\big)}{2} + z^3\frac{h'''(0_{\mathrm{sgn}(z)})}{6} + z^4\frac{h''''(\xi_z)}{24}\right]\right) \\
&= \sqrt{\frac{2\pi}{\beta|h''\big(0_{\mathrm{sgn}(z)}\big)|}}\phi_{\beta\,\mathrm{sgn}(z)}(z)\exp\left(\beta z^3\frac{h'''(0_{\mathrm{sgn}(z)})}{6}\right)\exp\left(\beta z^4\frac{h''''(\xi_z)}{24}\right) \qquad (99)
\end{aligned}
$$

where $\phi_{\beta\,\mathrm{sgn}(z)}(\cdot)$ denotes the pdf of a $\mathcal{N}\left(0, \left(\beta|h''\big(0_{\mathrm{sgn}(z)}\big)|\right)^{-1}\right)$.

By further Taylor expansion,

$$
\exp\left(\beta z^3\frac{h'''(\xi_z)}{6}\right) = 1 + \beta z^3\frac{h'''(0_{\mathrm{sgn}(z)})}{6} + \beta^2 z^6\frac{h'''(0_{\mathrm{sgn}(z)})^2}{72}e^{\xi^1_{(\beta,z)}} \qquad (100)
$$

$$
\exp\left(\beta z^4\frac{h''''(\xi_z)}{24}\right) = 1 + \beta z^4\frac{h''''(\xi_z)}{24}e^{\xi^2_{(\beta,z)}} \qquad (101)
$$

where $\xi^k_{(\beta,z)}$ are Taylor remainders such that

$$
\left|\xi^1_{(\beta,z)}\right| \le \left|\beta z^3\frac{h'''(0_{\mathrm{sgn}(z)})}{6}\right| \qquad (102)
$$

$$
\left|\xi^2_{(\beta,z)}\right| \le \left|\beta z^4\frac{h''''(\xi_z)}{24}\right| \qquad (103)
$$

Combining (99) and (100)

$$
I_k^g\left(-M\beta^{-r}, M\beta^{-r}\right) = \sum_{m=1}^{6} T_m(k,g) \qquad (104)
$$

where

$$T_1(k,g) = \sqrt{\frac{2\pi}{\beta}} \int_{-M\beta^{-r}}^{M\beta^{-r}} \sqrt{\frac{1}{|h''(0_{\mathrm{sgn}(z)})|}} g(z) z^k \phi_{\beta\,\mathrm{sgn}(z)}(z)\mathrm{d}z$$

$$T_2(k,g) = \sqrt{\frac{2\pi}{\beta}} \int_{-M\beta^{-r}}^{M\beta^{-r}} \sqrt{\frac{1}{|h''(0_{\mathrm{sgn}(z)})|}} g(z)\beta z^{k+3} \frac{h'''(0_{\mathrm{sgn}(z)})}{6} \phi_{\beta\,\mathrm{sgn}(z)}(z)\mathrm{d}z$$

$$T_3(k,g) = \sqrt{\frac{2\pi}{\beta}} \int_{-M\beta^{-r}}^{M\beta^{-r}} \sqrt{\frac{1}{|h''(0_{\mathrm{sgn}(z)})|}} g(z)\beta^2 z^{k+6} \frac{h'''(0_{\mathrm{sgn}(z)})^2}{72} e^{\xi^1_{(\beta,z)}} \phi_{\beta\,\mathrm{sgn}(z)}(z)\mathrm{d}z$$

$$T_4(k,g) = \sqrt{\frac{2\pi}{\beta}} \int_{-M\beta^{-r}}^{M\beta^{-r}} \sqrt{\frac{1}{|h''(0_{\mathrm{sgn}(z)})|}} \beta z^{k+4} \frac{h''''(\xi_z)}{24} e^{\xi^2_{(\beta,z)}} \phi_{\beta\,\mathrm{sgn}(z)}(z)\mathrm{d}z$$

$$T_5(k,g) = \sqrt{\frac{2\pi}{\beta}} \int_{-M\beta^{-r}}^{M\beta^{-r}} \sqrt{\frac{1}{|h''(0_{\mathrm{sgn}(z)})|}} \beta^2 z^{k+7} \frac{h''''(\xi_z)h'''(0_{\mathrm{sgn}(z)})}{48} e^{\xi^2_{(\beta,z)}} \phi_{\beta\,\mathrm{sgn}(z)}(z)\mathrm{d}z$$

$$T_6(k,g) = \sqrt{\frac{2\pi}{\beta}} \int_{-M\beta^{-r}}^{M\beta^{-r}} \sqrt{\frac{1}{|h''(0_{\mathrm{sgn}(z)})|}} \beta^3 z^{k+10} \frac{h''''(\xi_z)h'''(0_{\mathrm{sgn}(z)})^2}{144} e^{\xi^2_{(\beta,z)}} e^{\xi^1_{(\beta,z)}} \phi_{\beta\,\mathrm{sgn}(z)}(z)\mathrm{d}z$$

For the higher order terms, we observe the following, with $g_* = \max(|g(x)|)$:

$$\begin{aligned}
|T_3(k,g)| &= \left| \sqrt{\frac{2\pi}{\beta}} \int_{-M\beta^{-r}}^{M\beta^{-r}} \sqrt{\frac{1}{|h''(0_{\mathrm{sgn}(z)})|}} g(z)\beta^2 z^{k+6} \frac{h'''(0_{\mathrm{sgn}(z)})^2}{72} e^{\xi^1_{(\beta,z)}} \phi_{\beta\,\mathrm{sgn}(z)}(z)\mathrm{d}z \right| \\
&\leq \sqrt{\frac{2\pi}{\beta|H|}} \beta^2 \frac{h_*^2 g_*}{72} \int_{-M\beta^{-r}}^{M\beta^{-r}} |z|^{k+6} \exp\left(\beta z^3 \frac{h_*}{6}\right) \phi_{\beta\,\mathrm{sgn}(z)}(z)\mathrm{d}z \\
&\leq \sqrt{\frac{2\pi}{\beta|H|}} \beta^2 \frac{h_*^2 g_*}{72} \exp\left(M^3 \frac{h_*}{6}\right) \int_{\mathbb{R}} |z|^{k+6} \phi_{\beta\,\mathrm{sgn}(z)}(z)\mathrm{d}z \\
&= C_3^k \beta^{-(k+3)/2} \int_{\mathbb{R}} |z|^{k+6} \mathcal{N}(z;0,1)\mathrm{d}z
\end{aligned} \tag{105}$$

Following analog methodologies, one can show the existence of $C_4^k, C_5^k, C_6^k \geq 0$ such that :

$$|T_4(k,g)| \leq C_4^k \beta^{-(k+3)/2} \int_{\mathbb{R}} |z|^{k+4} \mathcal{N}(z;0,1)\mathrm{d}z \tag{106}$$

$$|T_5(k,g)| \leq C_5^k \beta^{-(k+4)/2} \int_{\mathbb{R}} |z|^{k+7} \mathcal{N}(z;0,1)\mathrm{d}z \tag{107}$$

$$|T_6(k,g)| \leq C_6^k \beta^{-(k+5)/2} \int_{\mathbb{R}} |z|^{k+10} \mathcal{N}(z;0,1)\mathrm{d}z \tag{108}$$

Hence, combining (105), (106), (107) and (108) shows that for any $m \in \{3,4,5,6\}$,

$$|T_m(k,g)| \leq O\left(\frac{1}{\beta^{(k+3)/2}}\right) \tag{109}$$

For the two remaining terms, we have

$$\begin{aligned}
T_1(k,g) &= \sqrt{\frac{2\pi}{\beta}} \int_{-M\beta^{-r}}^{M\beta^{-r}} \sqrt{\frac{1}{|h''(0_{\operatorname{sgn}(z)})|}}\, g(z) z^k \phi_{\beta\,\operatorname{sgn}(z)}(z)\mathrm{d}z \\
&= \sqrt{\frac{\pi}{2\beta}} \left[\frac{g(0_+)}{\sqrt{|h''(0_+)|}} \int_{\mathbb{R}} |z|^k \phi_{\beta+}(z)\mathrm{d}z \right. \\
&\qquad \left. + (-1)^k \frac{g(0_-)}{\sqrt{|h''(0_-)|}} \int_{\mathbb{R}} |z|^k \phi_{\beta-}(z)\mathrm{d}z \right] + R_1(k,g) \\
&= \sqrt{\frac{\pi}{2\beta^{k+1}}} \left[\frac{g(0_+)}{\sqrt{|h''(0_+)|^{k+1}}} + (-1)^k \frac{g(0_-)}{\sqrt{|h''(0_-)|^{k+1}}} \right] \int_{\mathbb{R}} |z|^k \mathcal{N}(z;0,1)\mathrm{d}z + R_1(k,g).
\end{aligned} \tag{110}$$

Then, we have :

$$\begin{aligned}
T_2(k,g) &= \sqrt{\frac{2\pi}{\beta}} \int_{-M\beta^{-r}}^{M\beta^{-r}} \sqrt{\frac{1}{|h''(0_{\operatorname{sgn}(z)})|}}\, g(z)\, \beta z^{k+3} \frac{h'''(0_{\operatorname{sgn}(z)})}{6} \phi_{\beta\,\operatorname{sgn}(z)}(z)\mathrm{d}z \\
&= \sqrt{\frac{2\pi}{\beta}} \left[\frac{g(0_+)h'''(0_+)}{12\sqrt{|h''(0_+)|}} \int_{\mathbb{R}} \beta |z|^{k+3} \phi_{\beta+}(z)\mathrm{d}z \right. \\
&\qquad \left. + (-1)^{k+1} \frac{g(0_-)h'''(0_-)}{12\sqrt{|h''(0_-)|}} \int_{\mathbb{R}} \beta |z|^{k+3} \phi_{\beta-}(z)\mathrm{d}z \right] + R_2(k,g) \\
&= \sqrt{\frac{2\pi}{\beta^{k+2}}} \left[\frac{g(0_+)h'''(0_+)}{12|h''(0_+)|^{(k+4)/2}} + (-1)^{k+1} \frac{g(0_-)h'''(0_-)}{12|h''(0_-)|^{(k+4)/2}} \right] \\
&\qquad \times \int_{\mathbb{R}} |z|^{k+3} \mathcal{N}(z;0,1)\mathrm{d}z + R_2(k,g).
\end{aligned} \tag{111}$$

By analogous reasoning to the proofs for propositions proposition 4 and proposition 5, one can show that there exist $F_1, F_2 > 0$ such that $R_1(k,g) = O\left(\beta^{(k+3)/2} e^{-F_1 \beta^{2/3}}\right)$, $R_2(k,g) = O\left(\beta^{(k+3)/2} e^{-F_2 \beta^{2/3}}\right)$ and trivially $R_1(k,g) + R_2(k,g) = O\left(\beta^{-(k+3)/2}\right)$.

Combining the results from (109), (110),(111), we get the anticipated result. □

A.2.2 Asymptotic Behaviour of the Logged Acceptance Ratio

First, the previous technical lemma allows us to derive some crucial asymptotic quantities for the asymptotic behaviour.

Corollary 2 (Moments)*.* Using the technical Lemma A.8 we derive the following quantities and their asymptotic behaviour,

$$N(\beta) = \sqrt{\frac{2\pi}{\beta|H|}}\left[1 + \frac{h'''(0_+) - h'''(0_-)}{12}\sqrt{\frac{8}{\pi\beta|H|^3}} + O\big(\beta^{-1}\big)\right], \tag{112}$$

$$\begin{aligned}\mu_\beta &:= \int_{\mathbb{R}} h'''\big(0_{\mathrm{sgn}(z)}\big)\, z^3\, \frac{f(z)^\beta}{N(\beta)}\mathrm{d}z \\ &= \sqrt{\frac{8}{\pi}}\,\frac{h'''(0_+) - h'''(0_-)}{2(\beta|H|)^{3/2}} \\ &\quad + \frac{1}{12\beta^2|H|^3}\Bigg[15\Big(h'''(0_+)^2 + h'''(0_-)^2\Big) \\ &\qquad - \frac{4}{\pi}\Big(h'''(0_+) - h'''(0_-)\Big)^2\Bigg] + O\big(\beta^{-5/2}\big),\end{aligned} \tag{113}$$

$$\begin{aligned}\sigma_\beta &:= \int_{\mathbb{R}} h'''\big(0_{\mathrm{sgn}(z)}\big)^2\, z^6\, \frac{f(z)^\beta}{N(\beta)}\mathrm{d}z \\ &= \frac{15}{(\beta|H|)^3}\,\frac{h'''(0_+)^2 + h'''(0_-)^2}{2} + O\big(\beta^{-7/2}\big).\end{aligned} \tag{114}$$

Furthermore, we can derive the Taylor Expansion of h in the neighbourhood of 0 :

$$h(z) = -\frac{|H|}{2}z^2 + \frac{h'''\big(0_{\mathrm{sgn}(z)}\big)}{6}z^3 + \frac{h''''\big(0_{\mathrm{sgn}(z)}\big)}{24}z^4 + \frac{h'''''(\xi_z)}{120}z^5 \tag{115}$$

where $0 < |\xi_z| < |z|$.

The new logged acceptance ratio (60) then gives us :

$$\begin{aligned}B(d) = \sum_{i=1}^{d}\Bigg[&\frac{\beta}{6}\left(y_i^3 h'''\big(0_{\mathrm{sgn}(y_i)}\big) - x_i^3 h'''\big(0_{\mathrm{sgn}(x_i)}\big)\right) + \frac{\beta}{24}\left(y_i^4 h''''\big(0_{\mathrm{sgn}(y_i)}\big) - x_i^4 h''''\big(0_{\mathrm{sgn}(x_i)}\big)\right) \\ &+ \frac{\beta}{120}\left(y_i^5 h'''''(\xi_{y_i}) - x_i^5 h'''''(\xi_{x_i})\right)\Bigg]\end{aligned} \tag{116}$$

We decompose this sum in three distinct terms, according to the magnitude of each component :

$$W(d) := \sum_{i=1}^{d} \frac{\beta}{6} \left(y_i^3 h'''\left(0_{\text{sgn}(y_i)}\right) - x_i^3 h'''\left(0_{\text{sgn}(x_i)}\right)\right) \tag{117}$$

$$T_1(d) := \sum_{i=1}^{d} \frac{\beta}{24} \left(y_i^4 h''''\left(0_{\text{sgn}(y_i)}\right) - x_i^4 h''''\left(0_{\text{sgn}(x_i)}\right)\right) \tag{118}$$

$$T_2(d) := \sum_{i=1}^{d} \frac{\beta}{120} \left(y_i^5 h'''''(\xi_{y_i}) - x_i^5 h'''''(\xi_{x_i})\right) \tag{119}$$

Let's set

$$\sigma^2 = \frac{15\left(h'''(0_+)^2 + h'''(0_-)^2\right) - \frac{4}{\pi}\left(h'''(0_+) - h'''(0_-)\right)^2}{36\ell |H|^3} \tag{120}$$

Then, we have the three following lemmas to characterize the asymptotic behaviour of the terms we just introduced, which we will prove one by one,

Lemma A.9. *With $f(\cdot)$ as in Theorem 5.2,*

$$W(d) \overset{d\to\infty}{\Longrightarrow} \mathcal{N}\left(-\frac{\sigma^2}{2}, \sigma^2\right) \tag{121}$$

Lemma A.10. *With $f(\cdot)$ as in Theorem 5.2,*

$$T_1(d) \xrightarrow{\mathbb{P}} 0 \tag{122}$$

Lemma A.11. *With $f(\cdot)$ as in Theorem 5.2,*

$$T_2(d) \xrightarrow{\mathbb{P}} 0 \tag{123}$$

Proof of lemma A.9. We divide the proof between the current state $\mathbf{x}$ and the proposed state $\mathbf{y}$.

Behaviour of the x_i's

Define the following collection of random variables for $d \in \mathbb{N}$ and $1 \leq i \leq d$:

$$X_{(d,i)} := \frac{\beta}{6}\left(x_i^3 h'''\left(0_{\mathrm{sgn}(x_i)}\right) - \mu_\beta\right)$$

Lindeberg-Feller Conditions: To apply the result of the Lindeberg-Feller theorem, the Central Limit Theorem for triangular arrays, to the $X_{(d,i)}$'s, we need to prove that the following two conditions hold as $d \to \infty$

i) There exists $\sigma^2 \in \mathbb{R}_+$such that

$$\sum_{i=1}^{d} \mathbb{E}\left(X_{(d,i)}^2\right) \to \sigma^2 > 0 \tag{124}$$

ii) For all $\varepsilon > 0$ and with $A_\varepsilon^d := \left\{\left|X_{(d,i)}\right| > \varepsilon\right\}$

$$\lim_{d\to\infty} \sum_{i=1}^{d} \mathbb{E}\left[|X_{(d,i)}|^2 \mathbb{1}_{A_\varepsilon^d}\right] = 0 \tag{125}$$

The first L-F condition (i) is much easier to show as, we have

$$\begin{aligned}
\mathbb{E}\left[X_{(d,i)}^2\right] &= \frac{\beta^2}{36}\left(\sigma_\beta - \mu_\beta^2\right) \\
&= \frac{\beta^2}{72}\frac{1}{(\beta|H|)^3}\left[15\left(h'''(0^+)^2 + h'''(0^-)^2\right) - \frac{4}{\pi}\left(h'''(0^+) - h'''(0^-)\right)^2\right] + O(\beta^{-3/2}) \\
&= \frac{1}{72\beta|H|^3}\left[15\left(h'''(0^+)^2 + h'''(0^-)^2\right) - \frac{4}{\pi}\left(h'''(0^+) - h'''(0^-)\right)^2\right] + O(\beta^{-3/2})
\end{aligned}$$

Now recall that $\beta = ld$, it is immediate that

$$\sum_{i=1}^{d} \mathbb{E}\left[X_{(d,i)}^2\right] \underset{d\to+\infty}{\to} \frac{1}{72l|H|^3}\left[15\left(h'''(0^+)^2 + h'''(0^-)^2\right) - \frac{4}{\pi}\left(h'''(0^+) - h'''(0^-)\right)^2\right] \tag{126}$$

For the second condition, let $\varepsilon > 0$, then we have :

$$\mathbb{E}\left[|X_{(d,i)}|^2 \mathbb{1}_{A_\varepsilon^d}\right] = \frac{\beta^2}{36}\left[\mathbb{E}\left[x_i^6 h'''\big(0_{\mathrm{sgn}(x_i)}\big)^2 \mathbb{1}_{A_\varepsilon^d}\right] - 2\mu_\beta \mathbb{E}\left[x_i^3 h'''\big(0_{\mathrm{sgn}(x_i)}\big) \mathbb{1}_{A_\varepsilon^d}\right] + \mu_\beta^2 \mathbb{P}\big(A_\varepsilon^d\big)\right] \tag{127}$$

First, we note that the set A_ε^d can be divided as

$$\begin{aligned} A_\varepsilon^d &= \left\{x_i^3 h'''\big(0_{\mathrm{sgn}(x_i)}\big) > \mu_\beta + \frac{6\varepsilon}{\beta}\right\} \cup \left\{x_i^3 h'''\big(0_{\mathrm{sgn}(x_i)}\big) < \mu_\beta - \frac{6\varepsilon}{\beta}\right\} \\ &= F_{+\varepsilon}^d \cup F_{-\varepsilon}^d \end{aligned} \tag{128}$$

Knowing that $\mu_\beta = O\big(d^{-3/2}\big)$, for d sufficiently large, there exists constants $J_1, J_2 > 0$ such that :

$$\begin{aligned} F_{+\varepsilon}^d &= \left\{x_i^3 h'''\big(0_{\mathrm{sgn}(x_i)}\big) > \mu_\beta + \frac{6\varepsilon}{\beta}\right\} \\ &\subseteq \left\{x_i^3 h'''\big(0_{\mathrm{sgn}(x_i)}\big) > \frac{J_1}{d}\right\} \\ &\subseteq \left\{|x_i|^3 |h'''\big(0_{\mathrm{sgn}(x_i)}\big)| > \frac{J_1}{d}\right\} \\ &\subseteq \left\{|x_i| > \left(\frac{J_1}{h_* d}\right)^{1/3}\right\} \end{aligned} \tag{129}$$

$$\begin{aligned} F_{-\varepsilon}^d &= \left\{x_i^3 h'''\big(0_{\mathrm{sgn}(x_i)}\big) < \mu_\beta - \frac{6\varepsilon}{\beta}\right\} \\ &\subseteq \left\{x_i^3 h'''\big(0_{\mathrm{sgn}(x_i)}\big) < -\frac{J_2}{d}\right\} \\ &\subseteq \left\{-x_i^3 h'''\big(0_{\mathrm{sgn}(x_i)}\big) > \frac{J_2}{d}\right\} \\ &\subseteq \left\{|x_i|^3 |h'''\big(0_{\mathrm{sgn}(x_i)}\big)| > \frac{J_2}{d}\right\} \\ &\subseteq \left\{|x_i| > \left(\frac{J_2}{h_* d}\right)^{1/3}\right\} \end{aligned} \tag{130}$$

Now, by combining (128), (129) and (130), as well as setting $J = \left(\frac{\min(J_1,J_2)}{h_*}\right)^{1/3}$, we get :

$$A_\varepsilon^d \subseteq \left\{|x_i| > \left(\frac{J_1}{h_* d}\right)^{1/3}\right\} \cup \left\{|x_i| > \left(\frac{J_2}{h_* d}\right)^{1/3}\right\}$$
$$\subseteq \left\{|x_i| > \left(\frac{J}{d^{1/3}}\right)\right\} \tag{131}$$

Hence, we have, with M as defined in (13), $g^p \in \mathcal{S}_0$ such that $g^p(z) = h'''\big(0_{\text{sgn}(z)}\big)^p$, and $p \in \{0, 1, 2\}$

$$\mathbb{E}\left[x_i^k g^p(x_i) \mathbb{1}_{A_\varepsilon^d}\right] \leq \frac{1}{N(\beta)}\Big[I_k^{g^p}\big(Jd^{-1/3}, M\big) + I_k^{g^p}(M, +\infty)$$
$$+ I_k^{g^p}\big(-M, -Jd^{-1/3}\big) + I_k^{g^p}(-\infty, -M)\Big]. \tag{132}$$

Then, by propositions proposition 4 and proposition 5 there exist $W_1, W_2, W_3 > 0$ such that

$$\mathbb{E}\left[x_i^6 h'''\big(0_{\text{sgn}(x_i)}\big)^2 \mathbb{1}_{A_\varepsilon^d}\right] = O\Big(e^{-W_1 d^{1/3}}\Big)$$
$$\mu_\beta \mathbb{E}\left[x_i^3 h'''\big(0_{\text{sgn}(x_i)}\big) \mathbb{1}_{A_\varepsilon^d}\right] = O\Big(e^{-W_2 d^{1/3}}\Big)$$
$$\mu_\beta^2 \mathbb{E}\left[\mathbb{1}_{A_\varepsilon^d}\right] = O\Big(e^{-W_3 d^{1/3}}\Big)$$

Setting $W = \min(W_1, W_2, W_3)$, we have :

$$\mathbb{E}\left[x_i^6 h'''(\xi_{x_i})^2 \mathbb{1}_{A_\varepsilon^d}\right] - 2\mu_\beta \mathbb{E}\left[x_i^3 h'''(\xi_{x_i}) \mathbb{1}_{A_\varepsilon^d}\right] + \mu_\beta^2 \mathbb{P}\big(A_\varepsilon^d\big) = O\Big(e^{-W d^{1/3}}\Big) \tag{133}$$

Consequently, by (127) and (133), we have :

$$\lim_{d\to\infty} \sum_{i=1}^{d} \mathbb{E}\left(\left|X_{(d,i)}\right|^2 \mathbb{1}_{A_\varepsilon^d}\right) = 0 \tag{134}$$

Thus, both Lindeberg-Feller conditions hold and the conclusion follows :

$$-\sum_{i=1}^{d} X_{(d,i)} \Rightarrow \mathcal{N}\left(0, \frac{1}{72l|H|^3}\left[15\left(h'''(0^+)^2 + h'''(0^-)^2\right) - \frac{4}{\pi}\left(h'''(0^+) - h'''(0^-)\right)^2\right]\right) \tag{135}$$

Behaviour of the y_i's

This time, we consider the following collection $\left(Y_{(d,i)}\right)_{1\leq i\leq d}$ of random variables for $d \in \mathbb{N}^*$, such that

$$Y_{(d,i)} := \frac{\beta}{6}\left(y_i^3 h'''\left(0_{\text{sgn}(y_i)}\right) - \mu_y\right)$$

where, from standard computations using the known and standard distribution of the y_i's,

$$\mu_y := \mathbb{E}\left[y_i^3 h'''\left(0_{\text{sgn}(y_i)}\right)\right] = \sqrt{\frac{8}{\pi}}\sqrt{\frac{1}{(\beta|H|)^3}}\frac{h'''(0_+) - h'''(0_-)}{2} \tag{136}$$

$$\sigma_y := \mathbb{E}\left[y_i^6 h'''\left(0_{\text{sgn}(y_i)}\right)^2\right] = 15\frac{1}{(\beta|H|)^3}\frac{h'''(0_+)^2 + h'''(0_-)^2}{2} \tag{137}$$

one can follow an analogous procedure to comply with the Lindeberg-Feller theorem requirements and show that:

$$\sum_{i=1}^{d} Y_{(d,i)} \Rightarrow \mathcal{N}\left(0, \frac{1}{72l|H|^3}\left[15\left(h'''\left(0^+\right)^2 + h'''\left(0^-\right)^2\right) - \frac{4}{\pi}\left(h'''\left(0^+\right) - h'''\left(0^-\right)\right)^2\right]\right) \tag{138}$$

Using the fact that the x_i 's and y_i 's are independent along with the weak convergences established in (135) and (138), we get

$$\sum_{i=1}^{d}\left[Y_{(d,i)} - X_{(d,i)}\right] \Rightarrow \mathcal{N}\left(0, \frac{1}{36l|H|^3}\left[15\left(h'''\left(0^+\right)^2 + h'''\left(0^-\right)^2\right) - \frac{4}{\pi}\left(h'''\left(0^+\right) - h'''\left(0^-\right)\right)^2\right]\right). \tag{139}$$

Then, one can observe that :

$$\sum_{i=1}^{d}\left[Y_{(d,i)}-X_{(d,i)}\right]=\frac{\beta}{6}\sum_{i=1}^{d}\Big[y_i^3h'''\big(0_{\mathrm{sgn}(y_i)}\big)-x_i^3h'''\big(0_{\mathrm{sgn}(x_i)}\big)$$
$$-\mathbb{E}\left[y_i^3h'''\big(0_{\mathrm{sgn}(y_i)}\big)-x_i^3h'''\big(0_{\mathrm{sgn}(x_i)}\big)\right]\Big]$$
$$=\frac{\beta}{6}\sum_{i=1}^{d}\Big[y_i^3h'''\big(0_{\mathrm{sgn}(y_i)}\big)-x_i^3h'''\big(0_{\mathrm{sgn}(x_i)}\big)\Big]$$
$$-\frac{\ell d^2}{6}\mathbb{E}\left[y_i^3h'''\big(0_{\mathrm{sgn}(y_i)}\big)-x_i^3h'''\big(0_{\mathrm{sgn}(x_i)}\big)\right]$$
$$=B(d)-\frac{\ell d^2}{6}\mathbb{E}\left[y_i^3h'''\big(0_{\mathrm{sgn}(y_i)}\big)-x_i^3h'''\big(0_{\mathrm{sgn}(x_i)}\big)\right]. \tag{140}$$

Now, using the asymptotic formula recalled in (113),

$$\mathbb{E}\left[y_i^3h'''\big(0_{\mathrm{sgn}(y_i)}\big)-x_i^3h'''\big(0_{\mathrm{sgn}(x_i)}\big)\right]=-\frac{1}{12\beta^2|H|^3}\Bigg[15\left(h'''(0_+)^2+h'''(0_-)^2\right)$$
$$-\frac{4}{\pi}\left(h'''(0_+)-h'''(0_-)\right)^2\Bigg]+O\big(\beta^{-5/2}\big). \tag{141}$$

Finally, by plugging (141) into (140) and using (139), we show :

$$W(d)\Rightarrow\mathcal{N}\left(-\frac{\sigma^2}{2},\sigma^2\right) \tag{142}$$

with

$$\sigma^2=\frac{15\left(h'''(0_+)^2+h'''(0_-)^2\right)-\frac{4}{\pi}\left(h'''(0_+)-h'''(0_-)\right)^2}{36l|H|^3} \tag{143}$$

□

Proof of lemma A.10. Recall that we want to study the asymptotic behaviour of the following quantity (118):

$$T_1(d):=\sum_{i=1}^{d}\frac{\beta}{24}\left(y_i^4h''''\big(0_{\mathrm{sgn}(y_i)}\big)-x_i^4h''''\big(0_{\mathrm{sgn}(x_i)}\big)\right)$$

Through our findings of lemma A.8, we know:

$$\mathbb{E}\left[x_i^4 h''''\left(0_{\text{sgn}(x_i)}\right)\right] = 3\frac{h''''(0_+) + h''''(0_-)}{2\beta^2|H|^2} + O\left(\beta^{-5/2}\right) \tag{144}$$

$$\mathbb{E}\left[x_i^8 h''''\left(0_{\text{sgn}(x_i)}\right)^2\right] = 105\frac{h''''(0_+)^2 + h''''(0_-)^2}{2\beta^4|H|^4} + O\left(\beta^{-9/2}\right) \tag{145}$$

and using the fact that y_i's follow a Gaussian distribution,

$$\mathbb{E}\left[y_i^4 h''''\left(0_{\text{sgn}(y_i)}\right)\right] = 3\frac{h''''(0_+) + h''''(0_-)}{2\beta^2|H|^2} \tag{146}$$

$$\mathbb{E}\left[y_i^8 h''''\left(0_{\text{sgn}(y_i)}\right)^2\right] = 105\frac{h''''(0_+)^2 + h''''(0_-)^2}{2\beta^4|H|^4} \tag{147}$$

Defining $\mu_{T_1(d)} := \mathbb{E}\left(T_1(d)\right)$ then by (144) and (146), as $d \to \infty$

$$\mu_{T_1(d)} \to 0 \tag{148}$$

Thus there exists $U > 0$ such that for all $d > U$ then $\left|\mu_{T_1(d)}\right| < \frac{\varepsilon}{2}$. Furthermore, by (144), (145), (146) and (147) then

$$\text{Var}\left(T_1(d)\right) \to 0 \text{ as } d \to 0 \tag{149}$$

For $d > U$ and using Chebyshev's inequality and utilising (124)

$$\mathbb{P}\left(|T_1(d)| > \varepsilon\right) \leq \mathbb{P}\left(\left|T_1(d) - \mu_{T_1(d)}\right| \geq \frac{\varepsilon}{2}\right) \leq \frac{4\,\text{Var}\left(T_1(d)\right)}{\varepsilon^2} \to 0 \quad \text{as} \quad d \to 0$$

Hence,

$$T_1(d) \xrightarrow{\mathbb{P}} 0 \tag{150}$$

□

Proof of lemma A.11. Recall that we want to study the asymptotic behaviour of the following quantity (119) :

$$T_2(d) := \sum_{i=1}^{d} \frac{\beta}{120} \left(y_i^5 h'''''(\xi_{y_i}) - x_i^5 h'''''(\xi_{x_i}) \right)$$

To this end, using the assumption given in (20)

$$|T_2(d)| \leq \sum_{i=1}^{d} L \frac{\beta}{120} \left(|y_i|^5 + |x_i|^5 \right) =: T_2^r(d) > 0 \tag{151}$$

Once again using the conclusions of lemma A.8

$$\mathbb{E}\left[|x_i|^5\right] = 8\sqrt{\frac{2}{\pi\left(\beta|H|\right)^5}} + O\big(\beta^{-3}\big) \tag{152}$$

and through the Gaussian distributed y_i's

$$\mathbb{E}\left[|y_i|^5\right] = 8\sqrt{\frac{2}{\pi\left(\beta|H|\right)^5}} \tag{153}$$

Let $\varepsilon > 0$, then by Markov inequality and then using (152) and (153)

$$\begin{aligned}
\mathbb{P}\left(T_r(d) > \varepsilon\right) &\leq \frac{L\ell d}{24\varepsilon} \sum_{i=1}^{d} \left[\mathbb{E}\left(|y_i|^5\right) + \mathbb{E}\left(|x_i|^5\right)\right] \\
&= \frac{L\ell d^2}{24\varepsilon} \left[\mathbb{E}\left(|y_1|^5\right) + \mathbb{E}\left(|x_1|^5\right)\right] \\
&= \frac{L}{3\varepsilon}\sqrt{\frac{2}{\pi\ell^3 d|H|^5}} \\
&\underset{d\to\infty}{\rightarrow} 0
\end{aligned}$$

Then using (151), we have that

$$T_2(d) \xrightarrow{\mathbb{P}} 0 \tag{154}$$

□

A.2.3 Deriving the final result

Proof of Theorem 5.2. Merging the findings from lemmas A.9 to A.11 and applying Slutsky's Theorem gives us asymptotically, when $d \to \infty$,

$$B(d) = W(d) + T_1(d) + T_2(d) \Rightarrow B \sim \mathcal{N}\left(-\frac{\sigma^2}{2}, \sigma^2\right) \tag{155}$$

Leveraging the convergence in distribution and $z \mapsto \min(1, e^z)$ being a bounded continuous function we have,

$$\lim_{d\to\infty} \mathbb{E}\left[\min\left(1, e^{B(d)}\right)\right] = \mathbb{E}\left[\min\left(1, e^{B}\right)\right] \tag{156}$$

Proposition 6. Take $Z \sim \mathcal{N}(\mu, \sigma^2)$, then

$$\mathbb{E}\left[\min\left(1, e^{Z}\right)\right] = \mathbf{\Phi}\left(\frac{\mu}{\sigma}\right) + \exp\left(\mu + \sigma^2/2\right)\mathbf{\Phi}\left(-\sigma - \frac{\mu}{\sigma}\right) \tag{157}$$

Proof. Simple computation (Roberts et al., 1997, see, e.g.,). □

Using proposition 6 with the result established in equation (155) and with σ as in (120), we obtain the final asymptotic result presented in Theorem 5.2. □

B The HAT distributions

B.1 Weight-Stabilised

In Section 3.1 we introduced the HAT target distribution that we use as the target distributions for the annealed densities in the PA component of ALPS. This section explains why (at least to first order) these approximately preserve the modal weight upon annealing.

Much of this follows the motivation and arguments used in Tawn et al. (2019). We start by introducing a specific mixture distribution where all components consist of a scale and location transformation of some common density.

Definition B.1 (Componentwise Rescaled Mixture Distribution (CRMD)). $\forall j \in \{1, \dots, J\}$ let $\mu_j \in \mathbb{R}^d$ and $\Sigma_j \in \mathbb{R}^{d \times d}$ be positive definite matrices.

Define a component-wise rescaled mixture distribution to be a mixture distribution with J components but where the component distributions are homogeneous up to a scale and location reparametrisation. Specifically, for $J \in \mathbb{N}$, $s_j(x) := \Sigma_j^{-1/2}(x - \mu_j)$ and for $g(\cdot)$, a d-dimensional density function with a global maximum at 0, the CRMD is defined as

$$\gamma(x) \propto \sum_{j=1}^{J} a_j(x) = \sum_{j=1}^{J} w_j |\Sigma_j|^{-1/2} g\left(s_j(x)\right)) \tag{158}$$

where $\forall j \in \{1, \dots, J\}$ $\mu_j \in \mathbb{R}^d$ and $\Sigma_j \in \mathbb{R}^{d \times d}$ is a positive definite matrix.

In what follows we make the added assumption that the modes are well-separated which in this context means that there is sufficient distance between the location parameters, the μ_j's, such that there is negligible tail-overlap from the components. This is more reasonable assumption in high dimensional settings or indeed in cases where the distribution becomes annealed.

Upon annealing/tempering, either using some local or global scheme, then due to the assumed large separation the resulting distribution will still resemble a mixture distribution as described in Definition B.2.

Definition B.2 (Componentwise Tempered Rescaled Mixture Distribution (CTRMD)). Under the setting of Definition B.1, the component-wise tempered rescaled mixture distribution at inverse-temperature level β, γ_β, is defined as the CRMD given by

$$\gamma_\beta(x) \propto \sum_{j=1}^{J} b_j(x,\beta) = \sum_{j=1}^{J} W_{(j,\beta)} \frac{\left[g\left(s_j(x)\right)\right]^\beta}{\int \left[g\left(s_j(u)\right)\right]^\beta du}. \tag{159}$$

where $s_j(x) := \Sigma_j^{-1/2}(x-\mu_j)$.

We know that if traditional tempering is used then the effective weights of the components become increasingly inconsistent with the original mixture. The following proposition shows what happens under traditional tempering vs a scheme that re-weights the component according to its (local) maxima.

Proposition 7 (CTRMD Equivalences). Consider the setting of (158) and (159). Then $\forall j \in 1, \ldots, J$

- Standard power-tempering

  If $b_j(x,\beta) = [a_j(x)]^\beta$ then

  $$W_{(j,\beta)} \propto w_j^\beta |\Sigma_j|^{\frac{1-\beta}{2}}.$$

- Weight-preserving tempering

  If

  $$b_j(x,\beta) = a_j(x)^\beta a_j(\mu_j)^{(1-\beta)}$$

  then $W_{(j,\beta)} \propto w_j$.

Proof. The proof of this result is a routine extension of the proof given for the specific case of Gaussian mixture targets given in Tawn et al. (2019). □

The significance of this is that in cases where the target distribution can be approximated by a mixture of the form given in (158) then the HAT densities prevent the severe weight degeneracy issues that arise from traditional power-tempering methods.

B.2 Truncated HAT distributions

In Section 7.2, the ALPS procedure was used to sample from a posterior distribution that resulted from an SUR model. The condition number of the Hessian evaluations showed that the problem was unstable. The issue is that the problem is ill-posed in that the parameters are essentially unidentifiable. The posterior distribution seems to exhibit narrow ridges with tail-structures in those directions appearing to be significantly heavier than Gaussian.

As such there would need to be significant localised annealing before the target distribution along the ridge directions began to appear sufficiently Gaussian so that the independence sampler could provide reasonable inter-modal jump rates. However, this led to issues with machine precision. To overcome this issue, an ad-hoc adjustment to the HAT targets was proposed. The idea was to introduce a truncated version of the annealed HAT targets that would be exactly as described in Definition 3.1 if the location was within a prescribed distance from the mode and would be set to zero outside of that. The idea being to eliminate some of the instability of the heavy tailed ridge directions at the annealed temperature levels so that the algorithm works without using annealed temperature levels that would create computational problems due to machine precision issues.

Definition B.3 (Truncated HAT distributions at quantile q). Consider the definition of the HAT distribution at inverse temperature level β from Definition 3.1. Then using the same notation as Definition 3.1, and letting $A = A_{x,\beta}$ for ease of notation, set

$$Q_{x,\beta} := \mathbb{1}_{\{(x-\mu_A)^T \Sigma_A^{-1}(x-\mu_A)<q\}} \tag{160}$$

The truncated HAT distribution at inverse temperature level β and quantile q is then defined as:

$$\pi_\beta(x) \propto \begin{cases} \pi(x)^\beta \pi(\mu_{A_{x,\beta}})^{1-\beta} Q_{x,\beta} & \text{if } A_{x,\beta} = A_{x,1} \\ \\ G(x,\beta) Q_{x,\beta} & \text{if } A_{x,\beta} \neq A_{x,1} \end{cases} \tag{161}$$

where,

$$G(x,\beta) = \frac{\pi(\mu_A)\left((2\pi)^d |\Sigma_A|\right)^{1/2} \phi\left(x|\mu_A, \frac{\Sigma_A}{\beta}\right)}{\beta^{d/2}}.$$

The user would choose q as some pre-specified quantile of a Chi-squared distribution with degrees of freedom d-the dimensionality of the problem. The intuition is that the term in the indicator for the object $Q_{x,\beta}$ in Definition B.3 would be Chi-squared d distributed if the local mode was Gaussian with mean μ_A and covariance matrix Σ_A. Therefore, the distribution would eliminate the distribution for values inconsistent with such a null hypothesis.

This overcame the issues for the SUR model but there is further work needed to explore if this ad-hoc solution is robust for similarly computationally unstable problems.

C Pseudo-code implementation of ALPS

Algorithm 1 Annealed Leap-Point Sampler (ALPS)

Require: Temperature schedule $\Delta = \{\beta_0, \beta_1, \dots, \beta_n\}$

Require: Initial mode collections $\{\hat{W}, \hat{M}, \hat{C}\}$

Require: Number of temperature swaps s

Require: Initial sample $X^0 = (x_0^0, \dots, x_n^0)$

Require: Total number of samples T

```
for t = 0 to T − 1 do
  Within-temperature moves:
    for j = 0, ..., n − 1 do
      Sample x_j^{t+1} ~ P_{β_j}(x_j^t, ·)
      (local exploration at intermediate temperatures; see (3.1), (3.4))
    end for
    Sample x_n^{t+1} ~ Q(x_n^t, ·)
    (global exploration at the coldest temperature β_n; see (3.5))
  Temperature swap moves:
    for i = 1, ..., s do
      Perform QuanTA-aided temperature swap
      (see (3.3))
    end for
    Set X^{t+1} ← (x_0^{t+1}, x_1^{t+1}, ..., x_n^{t+1})
  (Optional) Update mode collections:
    Update {Ŵ, M̂, Ĉ} through online running of the Exploration Component
    (see (3.6))
end for
```

D Example 4: 4-Modal 20-Dimensional Skew-Normal

The target distribution is given by a mixture of four evenly-weighted and well-separated twenty-dimensional skew-normal distributions with heterogeneous scaling of the modes and homogeneous skew:

$$\pi(x) \propto \sum_{k=1}^{4} \prod_{j=1}^{20} \frac{2}{\omega_k} \phi\left(\frac{x_j - (\mu_k)_j}{\omega_k}\right) \Phi\left(\alpha \frac{x_j - (\mu_k)_j}{\omega_k}\right)$$

where $\alpha = 10$, $\mu_1 = (20, 20, \ldots, 20) = -\mu_2$, $\mu_3 = (-10 - 10, \ldots, -10, 10, 10, \ldots, 10) = -\mu_4$, $\omega_1 = \omega_2 = 1$ and $\omega_3 = \omega_4 = 2$ and where $\phi(\cdot)$ and $\Phi(\cdot)$ denote the density and CDF of a standard normal distribution. This is similar to Example 1 in Section 7.1.1, but with 4 modes instead of 13.

We compare the performance of ALPS against PT and LAIS. LAIS only uses a single temperature level which is the target level. For ALPS, 7 temperature levels were used for the annealing part of the algorithm with a cooling schedule given by powers in the vector $(1, 4, 16, 64, 256, 1024, 4096)$. In contrast, PT required 14 temperature levels which were geometrically spaced with common ratio of 0.6. This gave the suggested optimal temperature swap rates of 0.234 between consecutive temperature level pairs along with a temperature that appeared hot enough to explore the entire state space. Even so, the PT algorithm fails in this example rather spectacularly because the hot state target distribution is significantly inconsistent with the target distribution.

In all three algorithms random walk Metropolis was utilised for the within temperature moves; in the case of ALPS and LAIS then the random walk Metropolis moves used pre-conditioned covariance structure informed by the covariance matrix estimated at the local

mode by the Laplace approximation. In all cases the within temperature moves were tuned to have the suggested optimal acceptance rate of 0.234.

The hardest aspect of tuning ALPS was regarding the hot-state mode finder. As mentioned previously, finding “narrow modes” is never guaranteed in the finite run of the algorithm if their location is unknown (Fong et al., 2019). In both LAIS and ALPS where the hot state mode finder is required, it was found that an inverse temperature of 0.000005 worked very well and rapidly found the modes; typically within a few thousand iterations of the algorithm. As one would expect the performance of the mode finder is sensitive to its tuning and therein the algorithm's success also. The observations made in these empirical studies has suggested that the temperature of the hot state should also move dynamically to increase the robustness of this part of the algorithm. With the tuning of the hot state tuned as described, the hot state mode finder typically discovered all four mode-points within the first 4000 iterations of the algorithm.

Each of the three algorithms was run a total of 10 times, where for fair comparability the chains were all initiated from the first mode, μ_1 in each instance. Furthermore, all chains were run long enough to generate 200,000 samples from the target temperature (including the burn-in samples). Figure 1 shows trace plots for the first component of the Markov chain in each of the three cases after a burn-in is removed. A successful outcome would be a plot where the trace line jumps between the marginal component's modes centred at values -20,-10,10 and 20. Figure 1 shows ALPS was the only algorithm to successfully jump regularly between the different modes whilst in contrast the other two algorithms fail dramatically. LAIS makes only a couple of moves, due to the failure of the Laplace approximation to

Table 1: Comparison of the observed average run times for each of the three algorithms.

Algorithm	ALPS	LAIS	PT
Run Time (sec)	276.7	57.2	529.6

accurately capture the shape of such heavily skewed modes. PT performs even worse, with the chain remaining trapped in the (very spiky) first mode and demonstrating clearly the weakness of PT in high dimensions when modes have heterogeneous scales Woodard et al. (2009b).

The trace plots alone show that ALPS has vastly outperformed LAIS and PT. Figure 2 shows the running estimate of the probability that the first marginal component of the target distribution is less than 1/2 for each of the 10 repetitions for each of the algorithms. The entity being estimated is $\mathbb{P}(X_1 < 1/2)$, which equals almost exactly 0.5. Therefore, for the ith iteration of the Markov chain with burn-in b, then the following functional is being plotted for each repetition

$$f(i) = \frac{1}{i-b-1} \sum_{j=b+1}^{i} \mathbb{1}_{\{x_1^j < 1/2\}} \tag{162}$$

The plot for the runs of ALPS show that all repetitions converge successfully towards the true value of 0.5 whilst PT and LAIS fail to estimate this probability successfully.

All three algorithms have been run to give the same output quantity at the target state. However, it takes different amounts of time to run each algorithm per iteration. Table 1 shows the average run time for each of the three algorithms across each of the 10 repetitions. The results in Table 1 are very complementary to ALPS since there appears

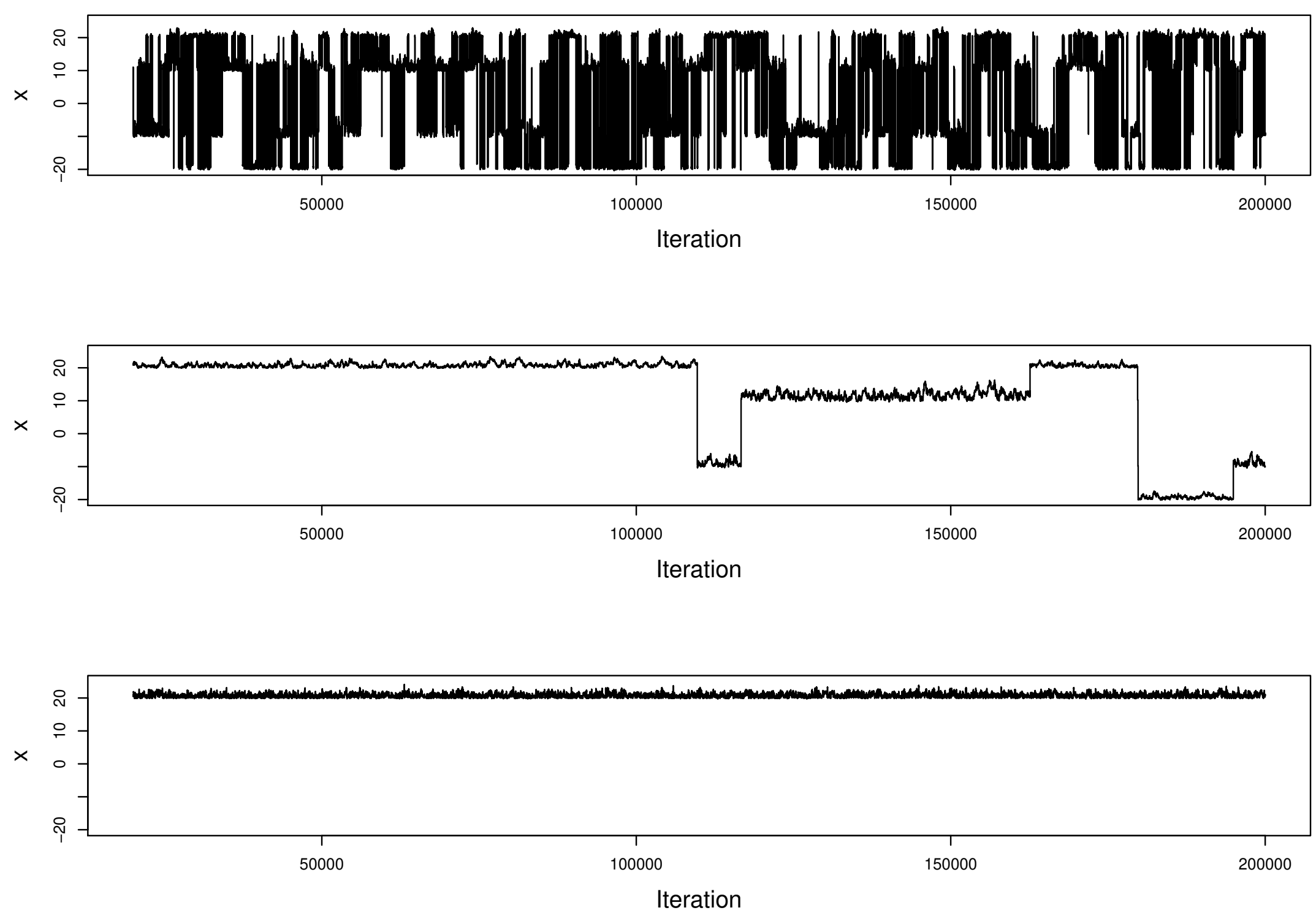

Figure 1: For the algorithms ALPS, LAIS and PT respectively, the trace plots of the first component of the Markov chain in a representative run from each of the 10 repetitions. ALPS (top) successfully explores the 4 modes centred on -20,-10,10 and 20 with rapid inter-modal mixing. In contrast LAIS only manages to make five jumps to another mode whilst the chain in PT remains trapped in the initialisation mode.

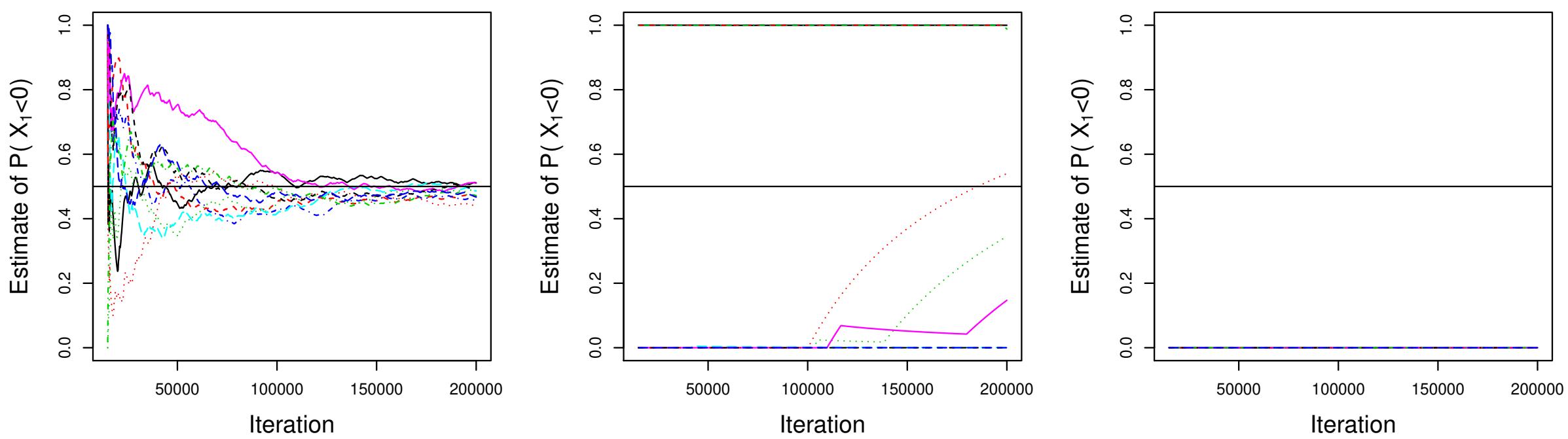

Figure 2: For the algorithms ALPS, LAIS and PT respectively, the function in equation 162 with a burn-in $b = 15,000$ is plotted for each of the 10 repetitions. For ALPS (leftmost plot) all trajectories concentrate around the truth by the end of their runs, whilst LAIS and PT fail dramatically in estimating this basic quantity.

to be little extra cost in running ALPS for what has been shown to be a dramatic gain in sample output. Indeed ALPS appears less expensive than PT and this is simply due to the vast number of extra temperatures that PT is updating per step of the chain in this example. However, in this example the number of modes was known and so the mode finding could be optimised for ALPS and LAIS; something not possible in a real data example where one may continue mode searching throughout the duration or even doing some pre-computation dedicated to mode searches (as was the case in the following real data example in Section 7.2).

E Seemingly-Unrelated Regression Model

SUR models involve a system of M linear regression equations, one for each response, $m = 1, \ldots, M$:

$$\vec{y}_m = \mathbf{X}_m \vec{\theta}_m + \vec{\epsilon}_m, \tag{163}$$

where $\vec{y}_m$ is a column vector of N_m observations for the mth response, $\vec{\theta}_m$ is a vector of J_m regression coefficients, $\mathbf{X}_m$ is an $N_m \times J_m$ design matrix of covariates, and $\vec{\epsilon}_m$ is a vector of residual errors.

For notational convenience, we assume that $N_1 = N_2 = \cdots = N_M = N$ and likewise that $J_1 = J_2 = \cdots = J_M = J$, although in full generality that is not always the case. For example, Kmenta (1997, p. 687) included a SUR model with $J_1 = 2$ and $J_2 = 3$ covariates.

The vectors of observations can be stacked on top of each other to form a single, longer vector $\left(\vec{y}_1^T, \vec{y}_2^T, \ldots, \vec{y}_M^T\right)^T$ and similarly with the regression coefficients $\left(\vec{\theta}_1^T, \vec{\theta}_2^T, \ldots, \vec{\theta}_M^T\right)^T$. The covariates $\mathbf{X}_m$ are combined to form a $MN \times MP$ block-diagonal matrix, resulting in the regression equation:

$$\begin{bmatrix} \vec{y}_1 \\ \vec{y}_2 \\ \vdots \\ \vec{y}_M \end{bmatrix} = \begin{bmatrix} \mathbf{X}_1 & & \ldots & \mathbf{0} \\ & \mathbf{X}_2 & & \vdots \\ \vdots & & \ddots & \\ \mathbf{0} & \ldots & & \mathbf{X}_M \end{bmatrix} \begin{bmatrix} \vec{\theta}_1 \\ \vec{\theta}_2 \\ \vdots \\ \vec{\theta}_M \end{bmatrix} + \begin{bmatrix} \vec{\epsilon}_1 \\ \vec{\epsilon}_2 \\ \vdots \\ \vec{\epsilon}_M \end{bmatrix}. \tag{164}$$

The errors $\vec{\epsilon}_m$ are assumed to be jointly multivariate normal,

$$\boldsymbol{\epsilon} \sim \mathcal{N}\left(\vec{0},\, \boldsymbol{\Sigma}_\epsilon \otimes \mathbf{I}\right),$$

where $\mathbf{I}$ is a $N \times N$ identity matrix and $\boldsymbol{\Sigma}_\epsilon$ is a $M \times M$ variance-covariance matrix with

entries $\sigma^2_{\ell,m}$. Zellner (1962) introduced an asymptotically-unbiased estimator for $\mathbf{\Sigma}_\epsilon$, conditional on estimates $\hat{\boldsymbol{\theta}}$ of the regression coefficients:

$$\hat{\sigma}^2_{\ell,m} = \frac{1}{N}\left(\vec{y}_\ell - \mathbf{X}_\ell \hat{\theta}_\ell\right)^T \left(\vec{y}_m - \mathbf{X}_m \hat{\theta}_m\right).$$

Given an estimate of $\widehat{\mathbf{\Sigma}}_\epsilon$, $\hat{\boldsymbol{\theta}}$ can be estimated using generalised least squares (Aitken, 1936). Let $\widehat{\mathbf{\Omega}} = (\widehat{\mathbf{\Sigma}}_\epsilon \otimes \mathbf{I})^{-1}$, then

$$\hat{\boldsymbol{\theta}} = \left(\mathbf{X}^T \widehat{\mathbf{\Omega}} \mathbf{X}\right)^{-1} \mathbf{X}^T \widehat{\mathbf{\Omega}} \mathbf{y}.$$

Alternating between these two estimators results in an iterative procedure that converges to a solution to the simultaneous regression equations.

The profile log-likelihood of the SUR model is thus:

$$\ell(\mathbf{Y} \mid \hat{\boldsymbol{\theta}}) = -N \log\{2\pi\} - \frac{N}{2} \log\left\{\left|\widehat{\mathbf{\Sigma}}_\epsilon\right|\right\} - N \tag{165}$$

Due to the quadratic form of the SUR model, it was long assumed that its likelihood was log-concave and therefore the iterative estimation procedure of Zellner (1962) was guaranteed to converge to the global maximum. For example, see Srivastava and Giles (1987, p. 157) and Greene (1997, p. 686). However, in fact these models are an example of a curved exponential family (Sundberg, 2010). Drton and Richardson (2004) showed in the bivariate case that maximising (165) is equivalent to finding the real roots of a fifth-degree polynomial. The profile log-likelihood preserves all stationary points of the log-likelihood, so if the likelihood is multi-modal then so will $\ell(\mathbf{Y} \mid \boldsymbol{\theta})$ (Drton and Richardson, 2004, Lemma 1). This means that the likelihood could have one, two, or three local maxima.

E.1 Further results

Additional results for ALPS with the SUR dataset from Grunfeld (1958) with $N = 15$ years of observations for $M = 5$ manufacturing firms and with $J = 3$ covariates per firm, are shown in Figures 3–17. For each parameter, panels (a) and (b) show marginal traceplots at the target inverse temperature $\beta = 1$ and coldest inverse temperature $\beta_{\max} = 32$; panel (c) shows the marginal histogram at the coldest inverse temperature. Red dashed lines indicate the marginal locations of the modes discovered by EC.

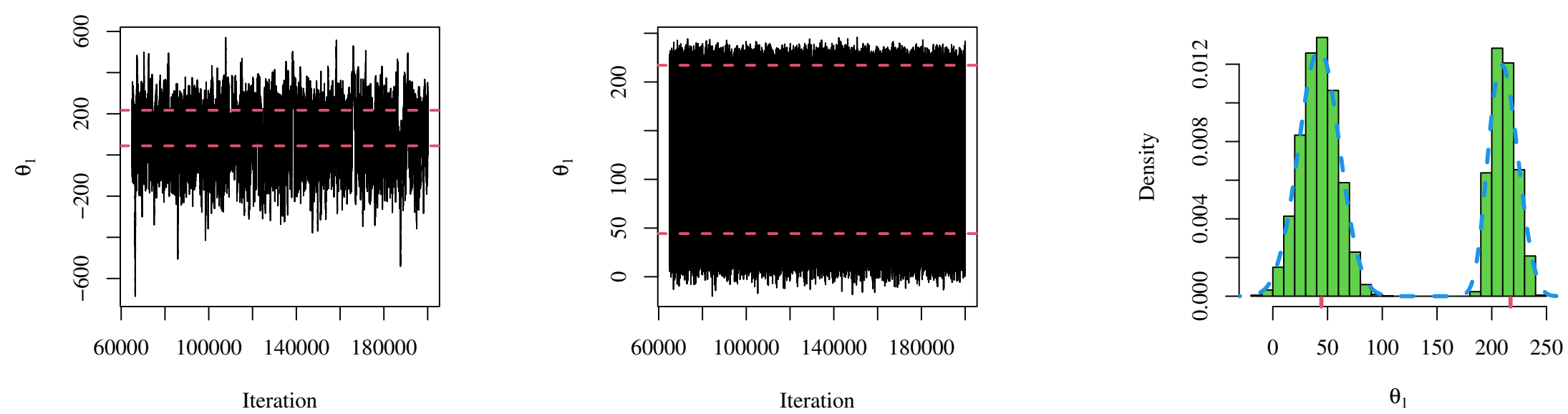

(a) Traceplot, θ_1, $\beta = 1$. (b) Traceplot, θ_1, $\beta_{\max} = 32$. (c) Histogram, θ_1, $\beta_{\max} = 32$.

Figure 3: Results for the marginal θ_1 chain.

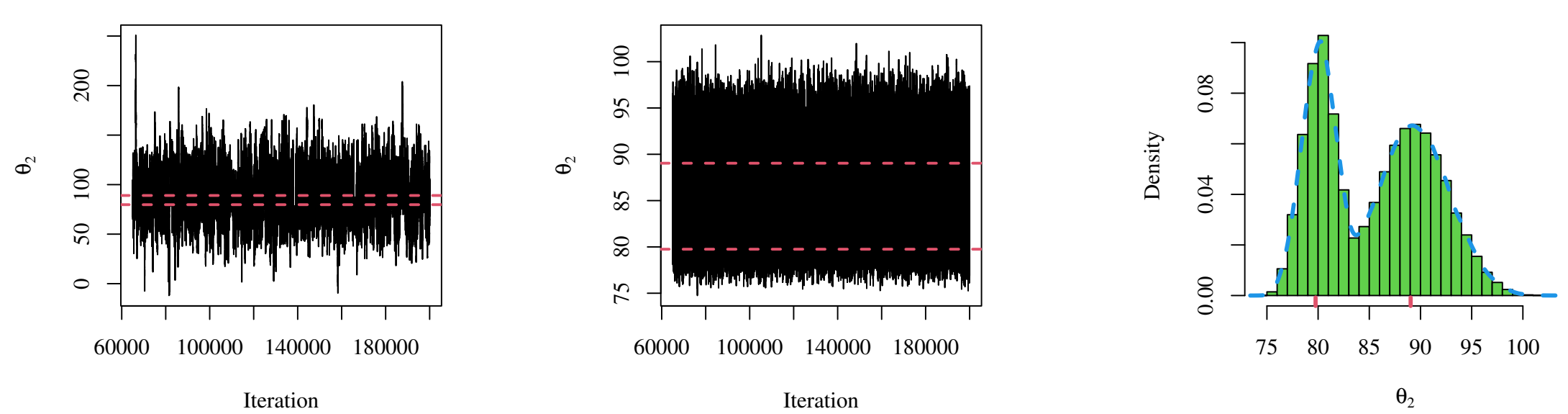

(a) Traceplot, θ_2, $\beta = 1$. (b) Traceplot, θ_2, $\beta_{\max} = 32$. (c) Histogram, θ_2, $\beta_{\max} = 32$.

Figure 4: Results for the marginal θ_2 chain.

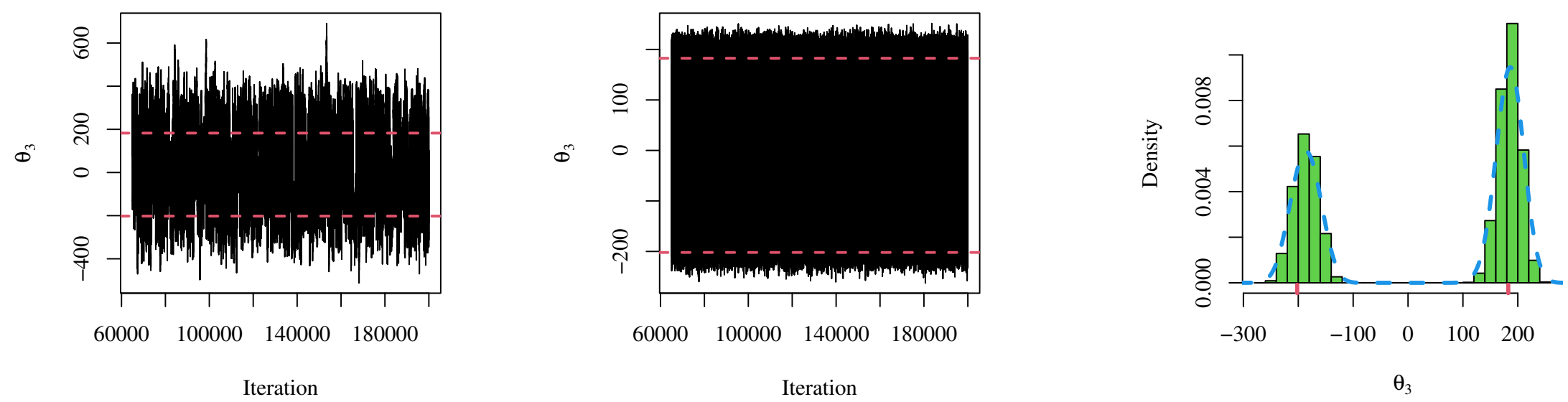

(a) Traceplot, θ_3, $\beta = 1$. (b) Traceplot, θ_3, $\beta_{\text{max}} = 32$. (c) Histogram, θ_3, $\beta_{\text{max}} = 32$.

Figure 5: Results for the marginal θ_3 chain.

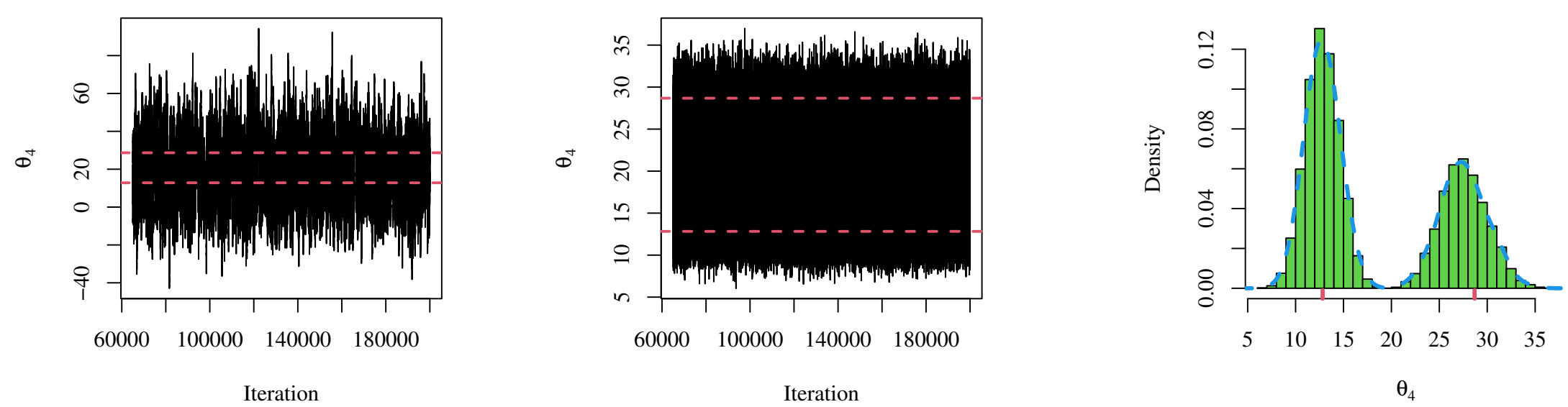

(a) Traceplot, θ_4, $\beta = 1$. (b) Traceplot, θ_4, $\beta_{\text{max}} = 32$. (c) Histogram, θ_4, $\beta_{\text{max}} = 32$.

Figure 6: Results for the marginal θ_4 chain.

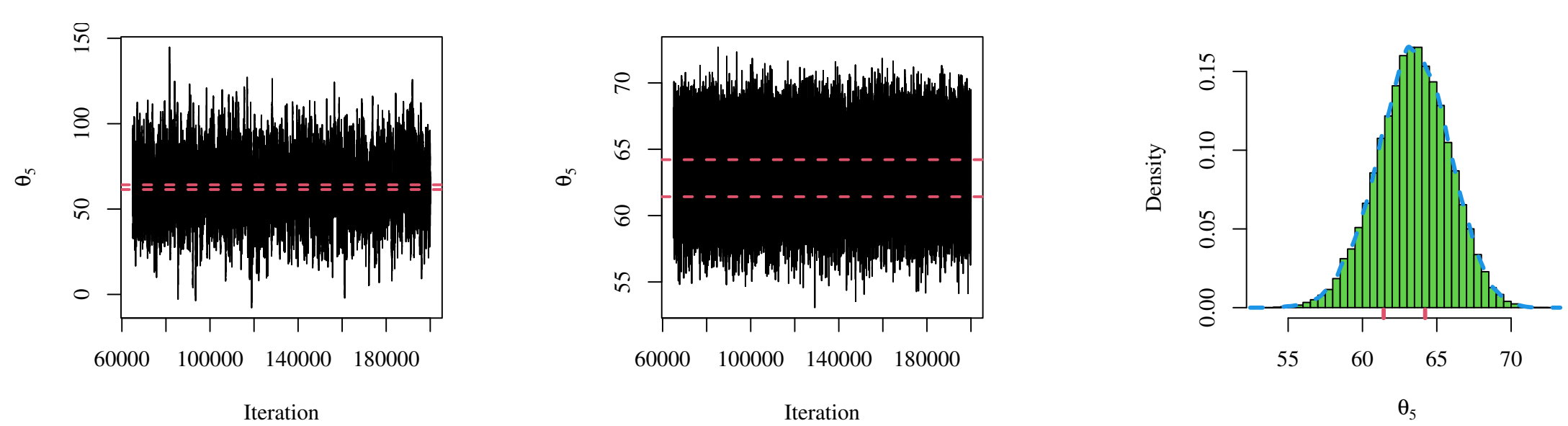

(a) Traceplot, θ_5, $\beta = 1$. (b) Traceplot, θ_5, $\beta_{\text{max}} = 32$. (c) Histogram, θ_5, $\beta_{\text{max}} = 32$.

Figure 7: Results for the marginal θ_5 chain.

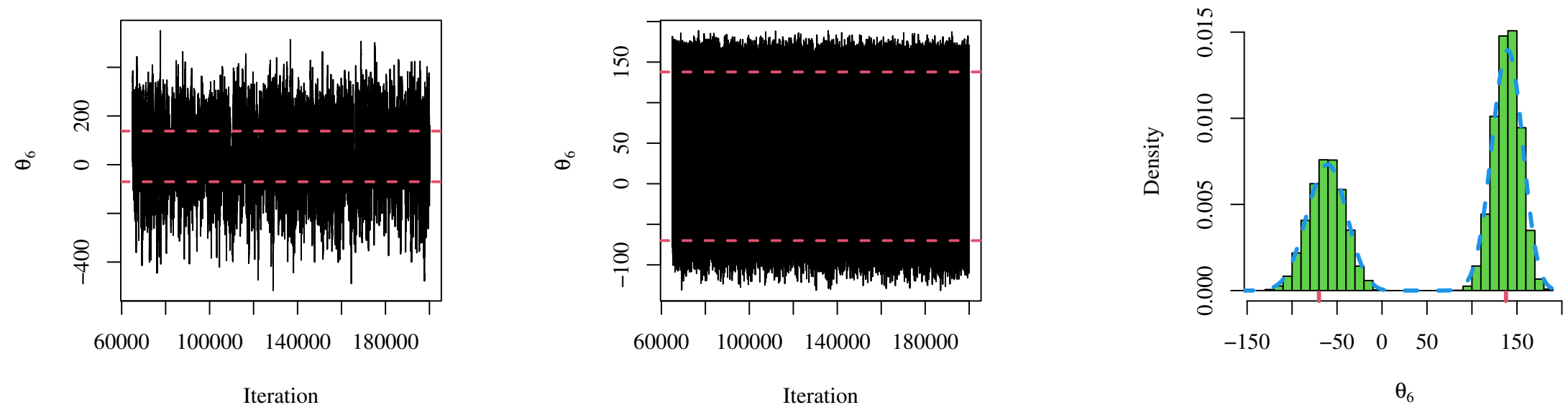

(a) Traceplot, θ_6, $\beta = 1$. (b) Traceplot, θ_6, $\beta_{\max} = 32$. (c) Histogram, θ_6, $\beta_{\max} = 32$.

Figure 8: Results for the marginal θ_6 chain.

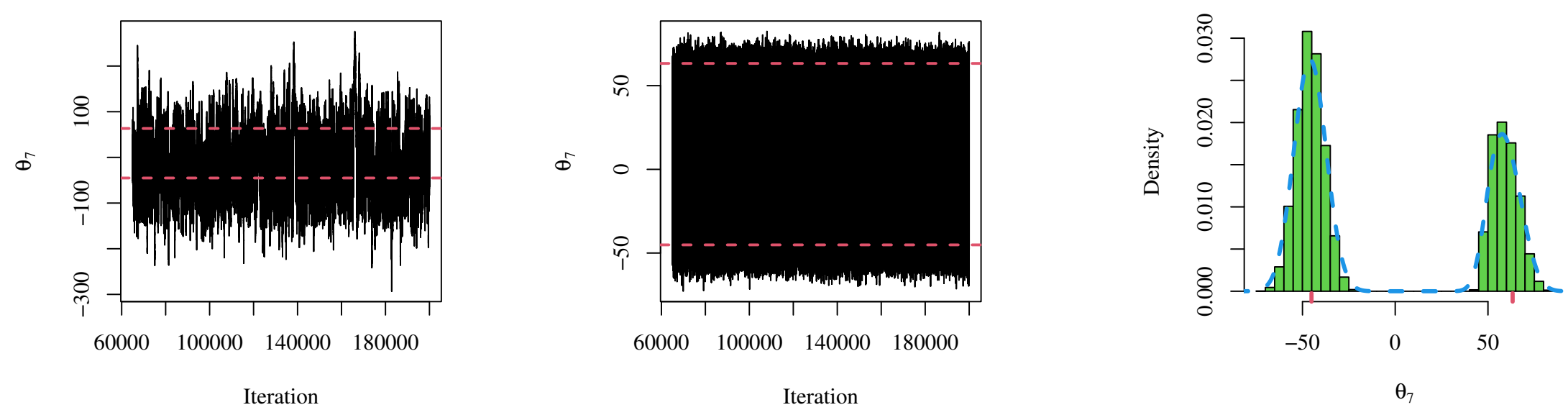

(a) Traceplot, θ_7, $\beta = 1$. (b) Traceplot, θ_7, $\beta_{\max} = 32$. (c) Histogram, θ_7, $\beta_{\max} = 32$.

Figure 9: Results for the marginal θ_7 chain.

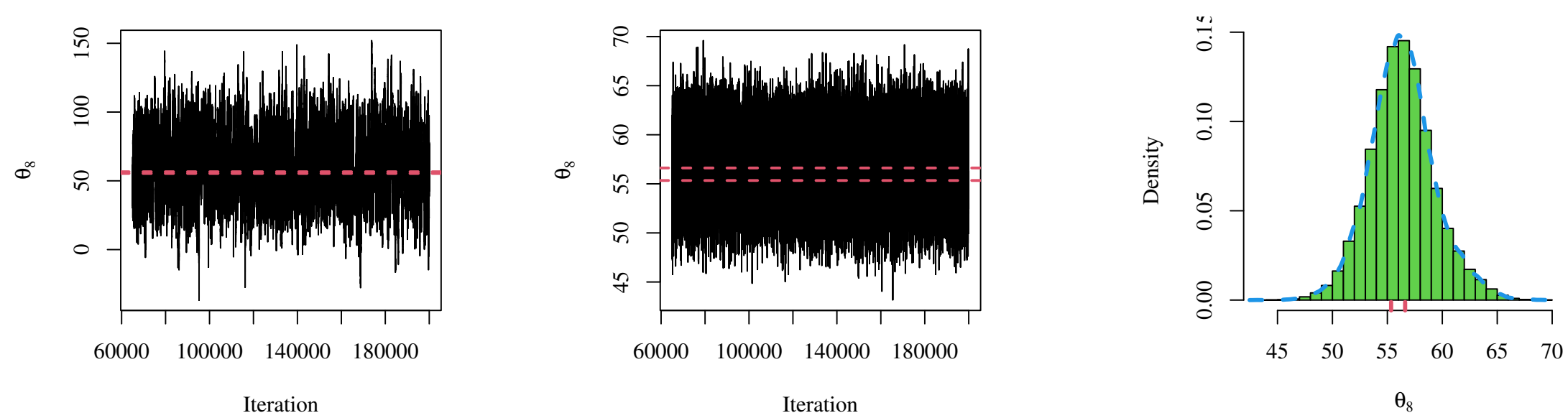

(a) Traceplot, θ_8, $\beta = 1$. (b) Traceplot, θ_8, $\beta_{\max} = 32$. (c) Histogram, θ_8, $\beta_{\max} = 32$.

Figure 10: Results for the marginal θ_8 chain.

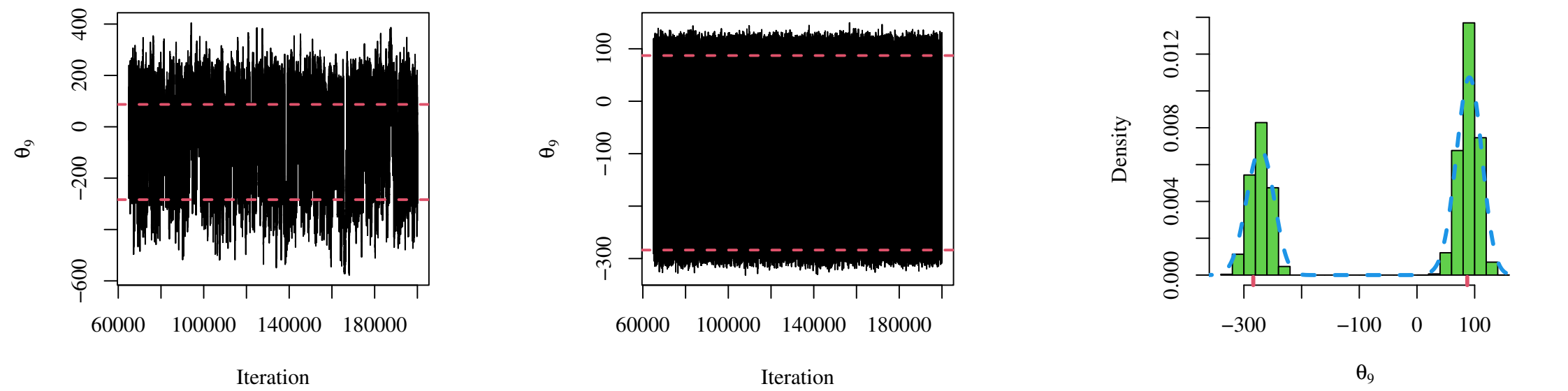

(a) Traceplot, θ_9, $\beta = 1$. (b) Traceplot, θ_9, $\beta_{\max} = 32$. (c) Histogram, θ_9, $\beta_{\max} = 32$.

Figure 11: Results for the marginal θ_9 chain.

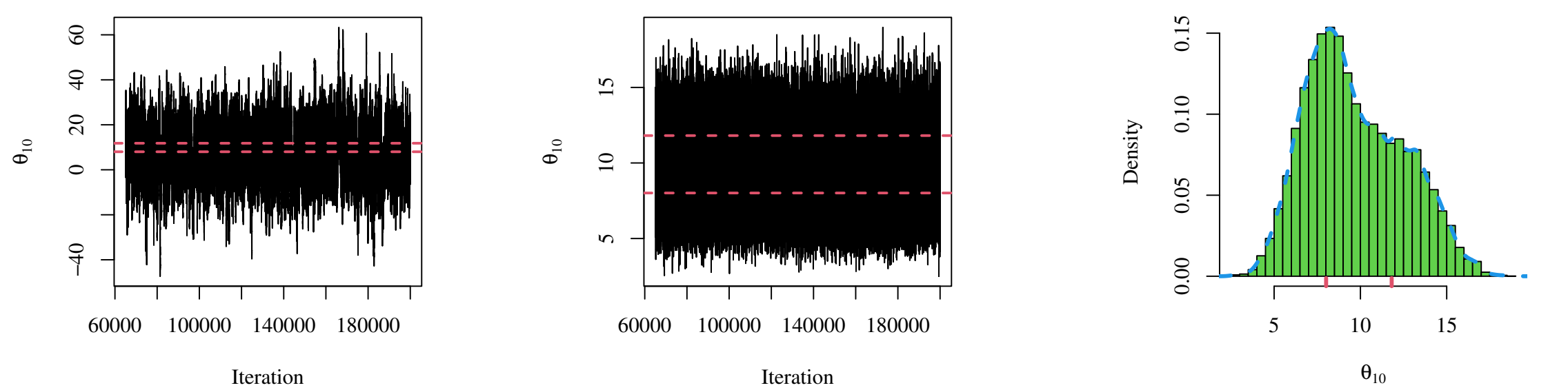

(a) Traceplot, θ_{10}, $\beta = 1$. (b) Traceplot, θ_{10}, $\beta_{\max} = 32$. (c) Histogram, θ_{10}, $\beta_{\max} = 32$.

Figure 12: Results for the marginal θ_{10} chain.

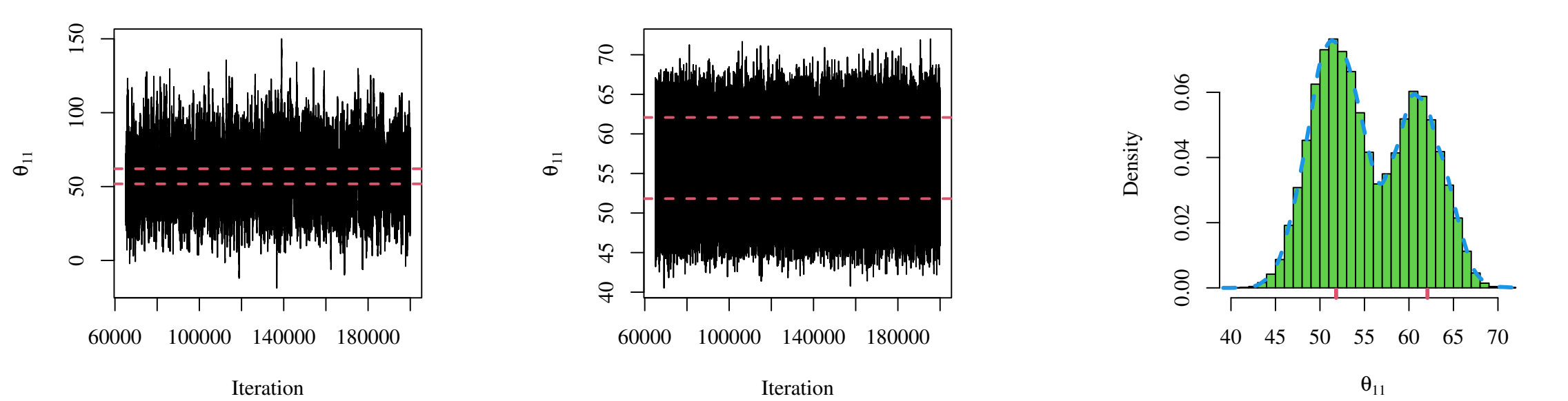

(a) Traceplot, θ_{11}, $\beta = 1$. (b) Traceplot, θ_{11}, $\beta_{\max} = 32$. (c) Histogram, θ_{11}, $\beta_{\max} = 32$.

Figure 13: Results for the marginal θ_{11} chain.

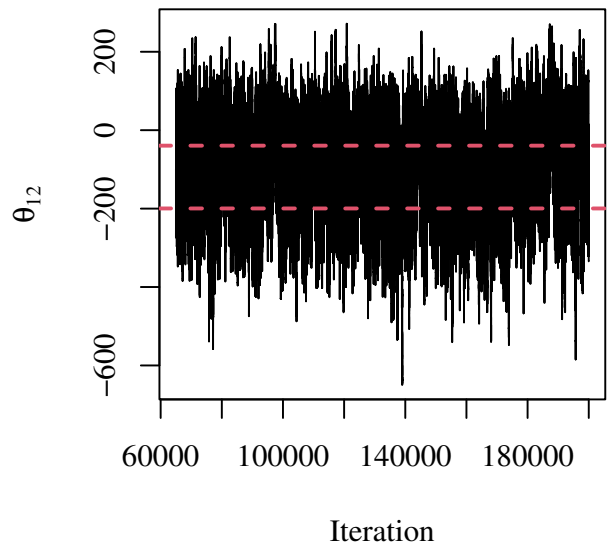

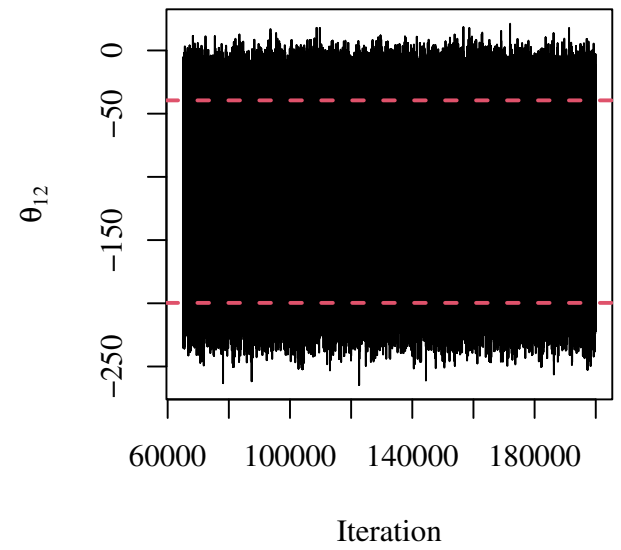

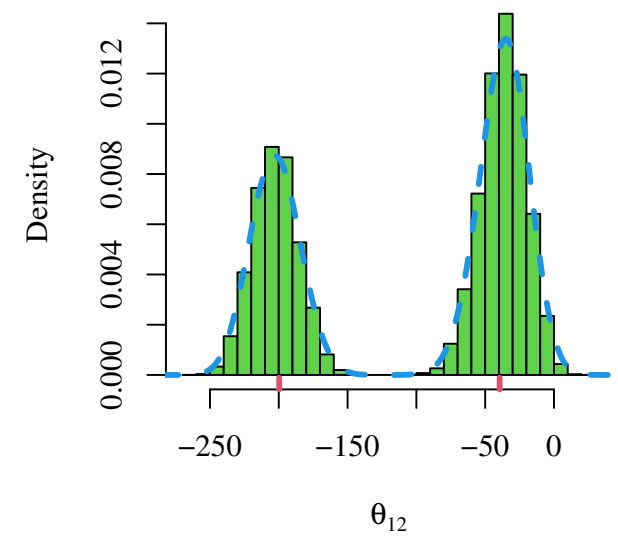

(a) Traceplot, θ_{12}, $\beta = 1$.

(b) Traceplot, θ_{12}, $\beta_{\max} = 32$.

(c) Histogram, θ_{12}, $\beta_{\max} = 32$.

Figure 14: Results for the marginal θ_{12} chain.

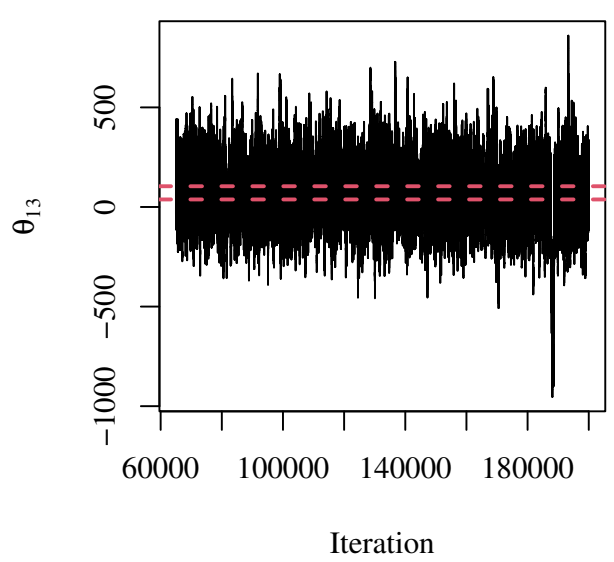

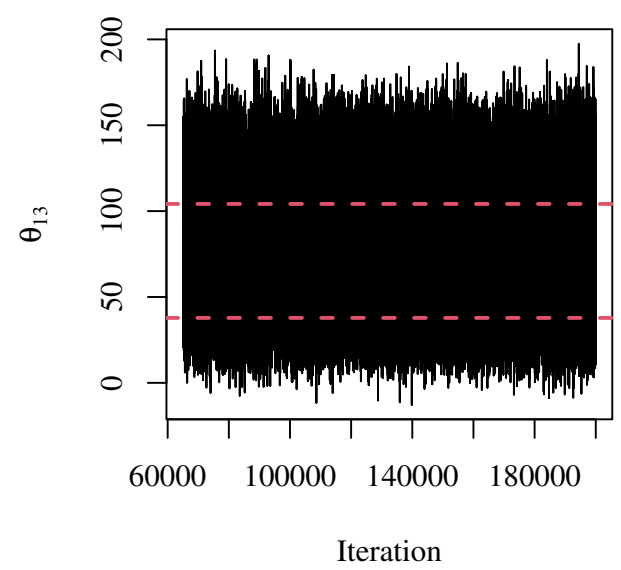

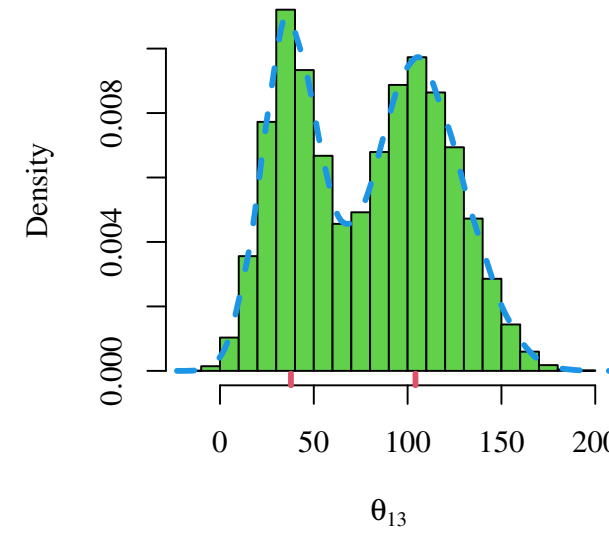

(a) Traceplot, θ_{13}, $\beta = 1$.

(b) Traceplot, θ_{13}, $\beta_{\max} = 32$.

(c) Histogram, θ_{13}, $\beta_{\max} = 32$.

Figure 15: Results for the marginal θ_{13} chain.

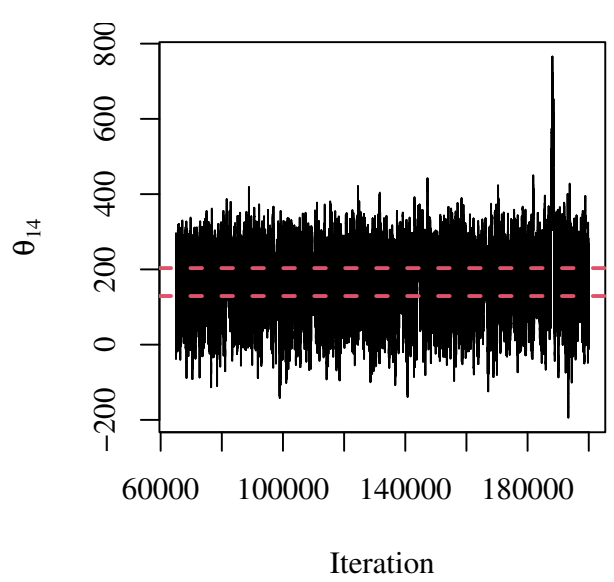

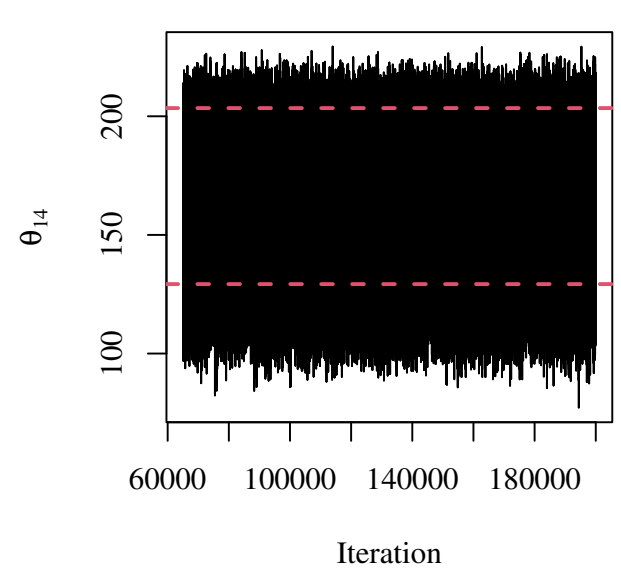

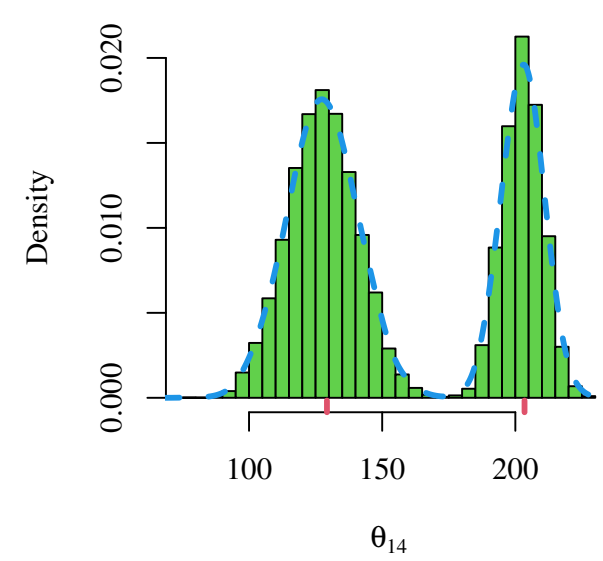

(a) Traceplot, θ_{14}, $\beta = 1$.

(b) Traceplot, θ_{14}, $\beta_{\max} = 32$.

(c) Histogram, θ_{14}, $\beta_{\max} = 32$.

Figure 16: Results for the marginal θ_{14} chain.

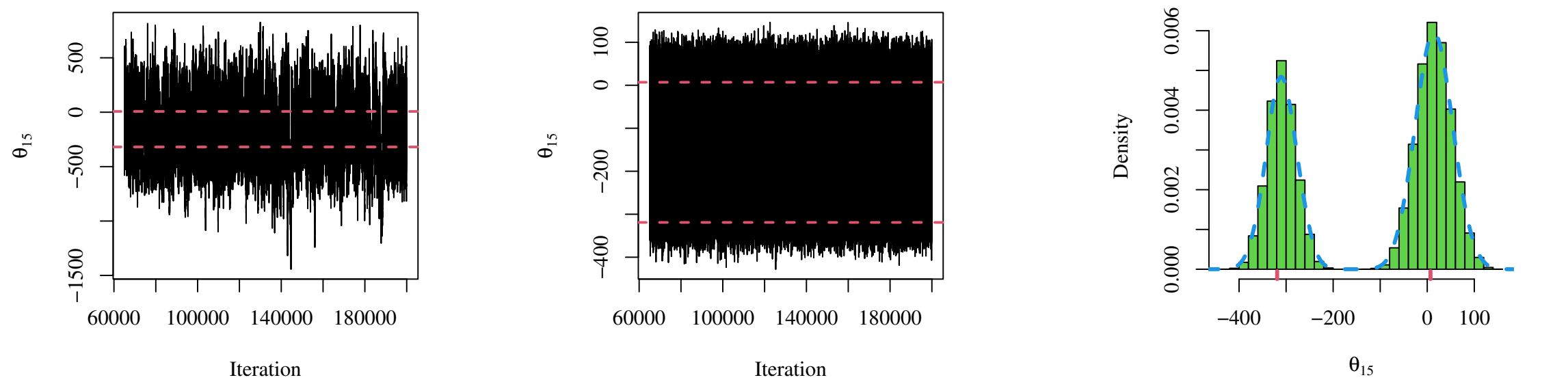

(a) Traceplot, θ_{15}, $\beta = 1$. (b) Traceplot, θ_{15}, $\beta_{\max} = 32$. (c) Histogram, θ_{15}, $\beta_{\max} = 32$.

Figure 17: Results for the marginal θ_{15} chain.
